# Analysis of aquaporins in Brassicaceae species reveals high-level of conservation and dynamic role against biotic and abiotic stress in canola

**DOI:** 10.1038/s41598-017-02877-9

**Published:** 2017-06-05

**Authors:** Humira Sonah, Rupesh K. Deshmukh, Caroline Labbé, Richard R. Bélanger

**Affiliations:** Département de phytologie–Faculté des Sciences de l’agriculture et de l’alimentation, Université Laval, Québec City, QC Canada

## Abstract

Aquaporins (AQPs) are of vital importance in the cellular transport system of all living organisms. In this study, genome-wide identification, distribution, and characterization of AQPs were determined in *Arabidopsis lyrata*, *Capsella grandiflora*, *C*. *rubella*, *Eutrema salsugineum*, *Brassica rapa*, *B*. *oleracea*, and *B*. *napus* (canola). Classification and phylogeny of AQPs revealed the loss of XIPs and NIP-IIIs in all species. Characterization of distinctive AQP features showed a high level of conservation in spacing between NPA-domains, and selectivity filters. Interestingly, TIP3s were found to be highly expressed in developing seeds, suggesting their role in seed desiccation. Analysis of available RNA-seq data obtained under biotic and abiotic stresses led to the identification of AQPs involved in stress tolerance mechanisms in canola. In addition, analysis of the effect of ploidy level, and resulting gene dose effect performed with the different combinations of Brassica A and C genomes revealed that more than 70% of AQPs expression were dose-independent, thereby supporting their role in stress alleviation. This first in-depth characterization of Brassicaceae AQPs highlights transport mechanisms and related physiological processes that could be exploited in breeding programs of stress-tolerant cultivars.

## Introduction

Aquaporins (AQPs) are integral membrane proteins that facilitate the transport of water and many other small molecules like urea, boric acid, silicic acid, ammonia, and carbon dioxide^1–3^. AQPs are found in all living organisms and localized in the plasma membrane, endoplasmic reticulum, vacuoles, plastids and other subcellular compartments^4, 5^. Most AQPs are characterized by six transmembranes (TM) alpha helices connected by five loops and highly conserved NPA (Asn-Pro-Ala) motifs^6^. The NPA motifs are the two half TM helices forming a constrict at the center of the pore that serves as a size selectivity barrier (Törnroth-Horsefield *et al*., 2006). Another constrict that defines the substrate specificity consists of four amino acids known as the aromatic/arginine (ar/R) selectivity filter (SF)^2, 7^. In addition to these two significant selectivity barriers, several other conserved features are known to play a role in AQP solute specificity including Froger’s residues composed of five conserved amino acids known to discriminate glycerol-transporting aquaglyceroporins (GLPs) from water-conducting AQPs^8^. Similarly, several specificity-determining positions (SDPs) have been proposed for urea, boric acid, silicic acid, ammonia, carbon dioxide and hydrogen peroxide^9^.

Plant AQPs constitute a large family of proteins that are categorized, based on phylogenetic distribution, into five major sub-families: plasma membrane intrinsic proteins (PIPs), tonoplast intrinsic proteins (TIPs), NOD26-like intrinsic proteins (NIPs), small basic intrinsic proteins (SIPs), and uncharacterized intrinsic proteins (XIPs)^10–12^. Unique patterns of subcellular localization have been observed in proteins within each AQP sub-family; for instance, PIPs are mostly localized to the plasma membrane, TIPs to the tonoplast, NIPs to the plasma membrane or the endoplasmic reticulum, SIPs to the endoplasmic reticulum, and XIPs mostly to the plasma membrane^4, 5^. Two additional subfamilies, GlpF-like intrinsic proteins (GIPs) and hybrid intrinsic proteins (HIPs), have also been described in primitive plant species, and are thought to have been lost by higher plants during the course of evolution^12, 13^. Similarly, loss of XIPs in *Arabidopsis* and monocots, and NIP2s (categorized as NIP-III) in *Arabidopsis* has been confirmed in a recent study highlighting genome-wide identification and comparison of AQPs in 25 plant species^12^.

To date, more AQP homologs have been identified in plants compared to animals. For instance, 35 AQPs in *Arabidopsis*, 34 in *rice*, 55 in *poplar* and 72 in *soybean* have been reported compared to only 13 in the human genome^12, 14^. This higher and differential number of AQPs in plants is likely the result of gene duplication and higher ploidy levels in plants^12, 15^. The AQP duplications in the plants may have occurred either by whole genome duplication, segmental duplication, or partly by ectopic recombination, replication slippage, or retrotransposon activity. The duplicated AQPs are believed to have undergone diversification and neo-functionalization leading to the evolution of different classes of specialized functions^12, 16^. Recently, Shi, *et al*.^17^ analyzed gene expression dose effect using resynthesized Arabidopsis tetraploids harboring varied copies of chromosomes from model plant species *Arabidopsis thaliana* and *Arabidopsis arenosa* genomes. They observed that the genes mostly involved in cell cycle, photosynthesis, and metabolism are dosage-dependent, whereas genes involved in biotic and abiotic stress tolerance mechanisms are dosage-independent. Similar genetic resources that have a combination of genomes and diverse ploidy levels are available for Brassica species^18^. *Brassica napus* is an important oilseed crop representing the third leading source of vegetable oil in the world, after soybean and palm oil. The *B*. *napus* (an amphidiploid with chromosome n = 19) family belongs to the Cruciferae (Brassicaceae) and was developed by natural interspecific hybridization between the diploid species *B*. *oleracea* (n = 9) and *B*. *rapa* (syn. *campestris*, n = 10). However, very little is known about AQP evolution and gene expression dynamics in these *Brassica species* or other members of Brassicaceae.

Aquaporins play a significant role in plant physiological processes like cell-elongation, seed germination, osmoregulation, phloem-sap movement, stomatal and leaf-water movement, cytoplasmic homeostasis, nutrient transport, and modulation of a plant’s response to biotic and abiotic stresses^19–21^. As a result, AQPs are extensively studied to define their role in the enhancement of tolerance to abiotic stresses such as salinity, flooding, drought, heat, cold and heavy metal toxicity^22–25^. However, fewer efforts have been applied to understand the possible role of AQPs in alleviating biotic stresses. Recently, Tian, *et al*.^26^ evaluated the role of AQPs in the induction of disease immunity pathways in Arabidopsis. They found that Arabidopsis AQP AtPIP1;4 is responsible for transporting pathogen-induced apoplastic hydrogen peroxide (H_2_O_2_) to the cytoplasm. The role of H_2_O_2_ as a regulatory and signaling molecule has been described in many cellular processes, like photosynthesis, photorespiration, stomatal movement, cell cycle, senescence, and stress response. Permeability to H_2_O_2_ has been previously observed in several AQP subfamilies PIPs, TIPs, NIPs and XIPs^27^.

The ever-growing availability of plant genomes and transcriptomes offers an unprecedented access to specific gene families^15^. In the present study, a genome-wide identification of AQPs was performed in *Arabidopsis lyrata*, *Capsella grandiflora*, *C*. *rubella*, *Eutrema salsugineum*, *B*. *rapa*, *B*. *oleracea* and *B*. *napus*. The AQPs were characterized by the presence of ar/R SF, Froger’s residues, NPA motifs, and other conserved domains. Furthermore, the expression profiling of AQPs using transcriptome sequencing data available for *B*. *napus* under biotic and abiotic stress from different tissues were analyzed and compared. Results obtained from this study provide the first comprehensive classification of AQPs in Brassicacea species and highlight their importance and possible roles in stress alleviation.

## Materials and Methods

### Genome-wide identification of AQPs

Genomic sequences and gene annotations for *A*. *lyrata*, *C*. *grandiflora*, *C*. *rubella*, and *E*. *salsugineum* were retrieved from Phytozome (https://phytozome.jgi.doe.gov), for *B*. *rapa*, and *B*. *oleracea* from Ensembl (http://useast.ensembl.org/index.html) and for *B*. *napus* from Genoscope database (http://www.genoscope.cns.fr/brassicanapus). The NCBI command-line BLAST utilities provided in BioEdit (version 7.0.9.0) software tool was used to create local databases of the transcriptome and proteome sequences of these species^28^. The putative AQPs were identified with BLASTp search performed using 141 known AQPs as query sequences against the local database. The query sequences included 34 rice, 35 Arabidopsis, and 72 soybean AQPs^11, 16, 29^. A cut-off e-value of 10^−5^ was applied to identify significant matches. Top hits from the multiple matches were extracted based on the highest bit score. The BLAST hits with less than a 100 bit-score were removed. Similarly, homologs of Si-efflux transporters were identified in the seven species using BLASTp search with known efflux transporters as described by Vivancos, *et al*.^30^

### Identification of NPA motifs and transmembrane domains in aquaporins

The batch mode of NCBI’s Conserved Domain Database (CDD, www.ncbi.nlm.nih.gov/Structure/cdd/cdd.shtml) was used for the identification of known conserved domains including NPAs. AQPs showing a single or missing NPA motif were manually examined and removed from further analysis after re-confirmation with other characteristics like protein length, TM domains, and signal peptides. Transmembrane domains were detected using TMHMM, SOSUI, and TOPCONS software tools (www.cbs.dtu.dk; http://bp.nuap.nagoya-u.ac.jp)^31^. The results were then manually curated for altered and missing transmembrane domains.

### Construction of homology-based tertiary protein structure

Tertiary protein (3D) structures for all the newly identified and previously reported AQPs were constructed using Phyre2 protein modeling server^32^. ProTSAV was used for quality assessment of the 3D structures^33^. (http://www.scfbio-iitd.res.in/software/proteomics/protsavzip.jsp)

### Structural features of AQPs

The various physical and chemical parameters for AQPs were determined using ProtParam (http://web.expasy.org/protparam/). The protein subcellular localization was predicted using CELLO sever (http://cello.life.nctu.edu.tw/). The TM domains were defined using TOPCONS, SOSUI, and TMHMM^31, 34, 35^. Prediction of the solute specificity was performed on the basis of dual NPA motifs, ar/R SF, Froger’s residues and homology with Arabidopsis AQPs.

### Classification and phylogeny of AQPs

CLUSTALW implemented in MEGA7 was used for multiple sequence alignment of AQPs^36^. The multiple sequence alignment was subjected to construct the phylogenetic tree using the maximum likelihood (ML) method. The stability of branch nodes in the phylogenetic tree was measured by performing 1000 bootstraps. Based on the phylogenetic distribution, AQPs were classified into PIP, TIP, NIP, and SIP sub-families and nomenclature of the AQPs were assigned as per the similarity with Arabidopsis AQPs.

### Aquaporin Co-expression network

Aquaporin expression data from the publicly available RNA-seq Bio-projects PRJNA331148, PRJNA311316, PRJNA256233 and PRJNA311067 were used to construct co-expression network. Details of the data are provided in Supplementary information 1 (Supplementary Table 1). The normalized expression data were used to determine the correlation by applying Pearson product-moment correlation coefficient (Pearson r) and correlation thresholds with lower percentile rank 5 and upper percentile rank 95 used to consider significant association. The entire list of canola AQPs was used as bit genes. The co-expression network analysis was performed with Comparative Co-Expression Network Construction and Visualization tool (CoExpNetViz)^37^. The network was visualized using Cytoscape V. 3.3.0^38^.

### Silicon accumulation in canola

To measure Si concentrations in canola, leaf and root samples were collected from four week-old plants growing in hydroponic solutions. Rice plants were used as positive control. For the Si + treatment, liquid potassium silicate (Kasil®, PQ Corporation) was used to obtain a maximum concentration of 1.7 mM Si, and an additional K (0–0–52) was added to the control (Si-) solution to compensate for the addition of K. Leaf and root samples were dried at 60 °C for at least two days and pulverised with a bead mill homogenizer (Omni Bead Ruptor 24, Omni International). Silicon concentrations were measured from five different plants with the X-ray fluorescence spectrometry method (Niton XL3t955 GOLDD + XRF) adapted from Vivancos *et al*.^30^.

### RNA-seq data analysis

Publicly available RNA-seq data for different canola tissues and conditions were used in the present study (Supplementary Table 1). To study expression of BnAQPs under drought conditions, RNA-seq data analysis was performed using CLC Genomic Workbench. The cDNA libraries constructed for the leaf and root tissues of *B*. *napus* grown under controlled and drought conditions were sequenced using Illumina HiSeq 2000^39^. The raw sequenced reads were processed, mapped to *B*. *napus* reference genome and the transcript abundance was normalized by the RPKM (reads per kilobase of exon per million fragments mapped) using CLC Genomic Workbench. All the parameters used for RNAseq data analysis were set to default except some changes like mapping parameters allowing for a maximum of two mismatches and the maximum of 2 hits per read. The normalized expression data for all *B*. *napus* AQPs were retrieved and analysed further in MultiExperiment Viewer (MeV_4-9-0) software tool (http://www.tm4.org/mev.html). The raw reads and analysed expression data for other studies were retrieved from the *Brassica napus* SRA and GEO database (http://www.ncbi.nlm.nih.gov/sra and http://www.ncbi.nlm.nih.gov/gds/). Details of Bioprojects SRA and GEO accession are provided in Supplementary Table 1. Further details of library preparation, experimental design, replication, read processing and statistical analysis are provided in Supplementary information 1.

## Results

### Genome-wide distribution of aquaporins in *Brassicaceae*

**Figure 1:**
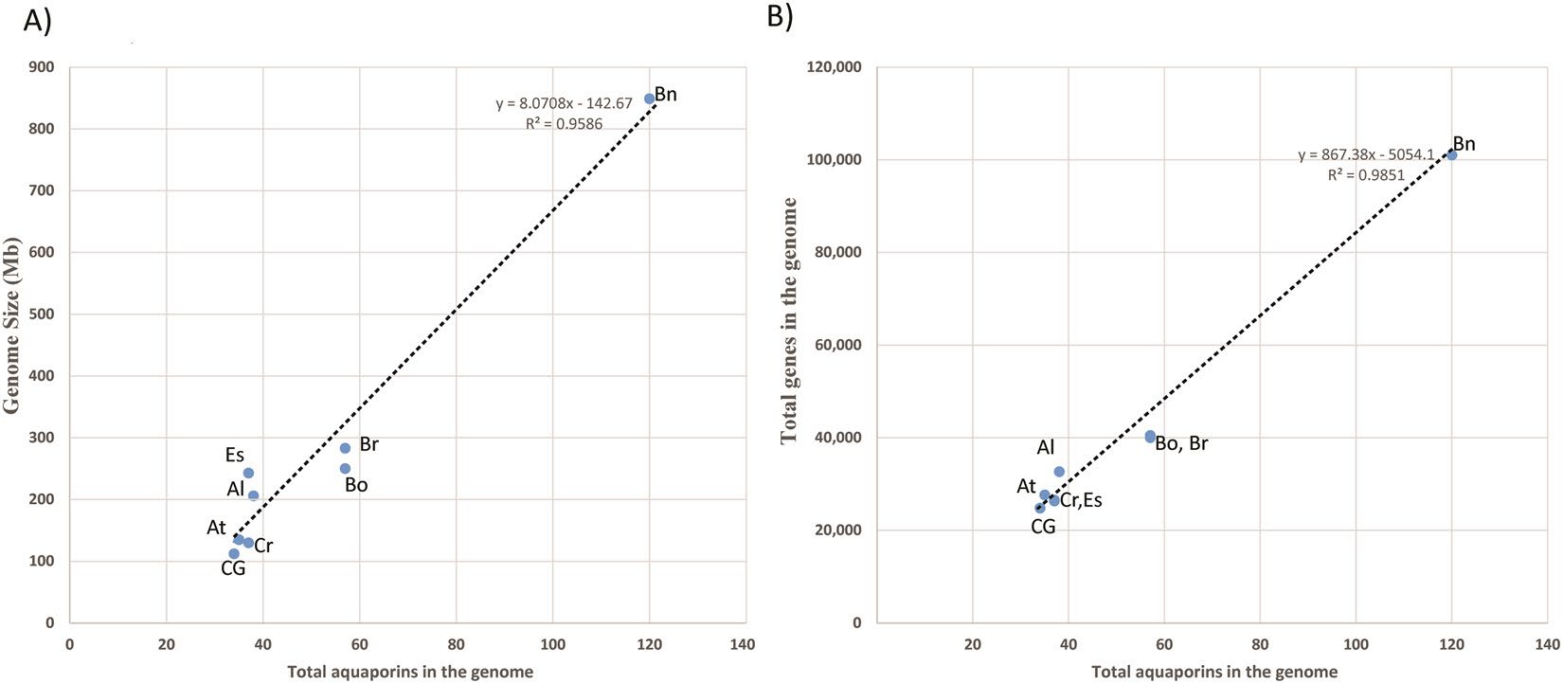
Relationship between number of aquaporins (AQPs), genome size and total number of predicted genes in the genomes. Total AQPs identified in *Arabidopsis thaliana* (At), *A*. *lyrata* (Al), *Capsella grandiflora* (Cg), *C*. *rubella* (Cr), *Eutrema salsugineum* (Es), *Brassica rapa* (Br), *B*. *oleracea* (Bo) and *B*. *napus* (Bn) was plotted against (**A**) the genome size and (**B**) total number of predicted genes.

Genome-wide identification performed in seven Brassicaceae species revealed a total of 380 AQPs. The AQPs were classified following a phylogenetic distribution, and named based on the Arabidopsis nomenclature. The overall pattern of AQPs distribution within the subfamilies was uniform across all Brassicaceae species analyzed (Table 1). As previously described in *Arabidopsis*, PIPs represented the highest number of AQPs followed by TIPs, NIPs, and SIPs, with the exception for *C*. *grandiflora* that has nine AQPs each in TIP and NIP categories. Species within the Arabidopsis clad had roughly the same number of AQPs (Table 1). In contrast, species within the *Brassica* clad had approximately two to four times more AQPs, which correlates precisely with the ploidy level of their respective genomes. The number of AQPs identified in each species was correlated with the genome size and the total number of predicted genes in the genome (Fig. 1).

**Table 1:**
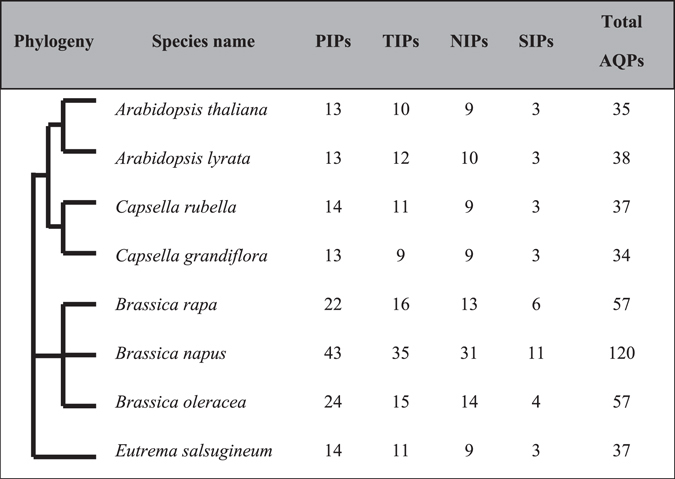
Genome-wide distribution of aquaporins in Brassicaceae species. PIP, Plasma membrane intrinsic protein; TIP, Tonoplast intrinsic protein; NIP, Noduline-26 like intrinsic protein; SIP, Small intrinsic protein; AQP, Aquaporin. Phylogeny of the species was constructed using Phylogenetic tree generator (http://phylot.biobyte.de/).

### Phylogenetic distribution of aquaporins in Brassicaceae

**Figure 2:**
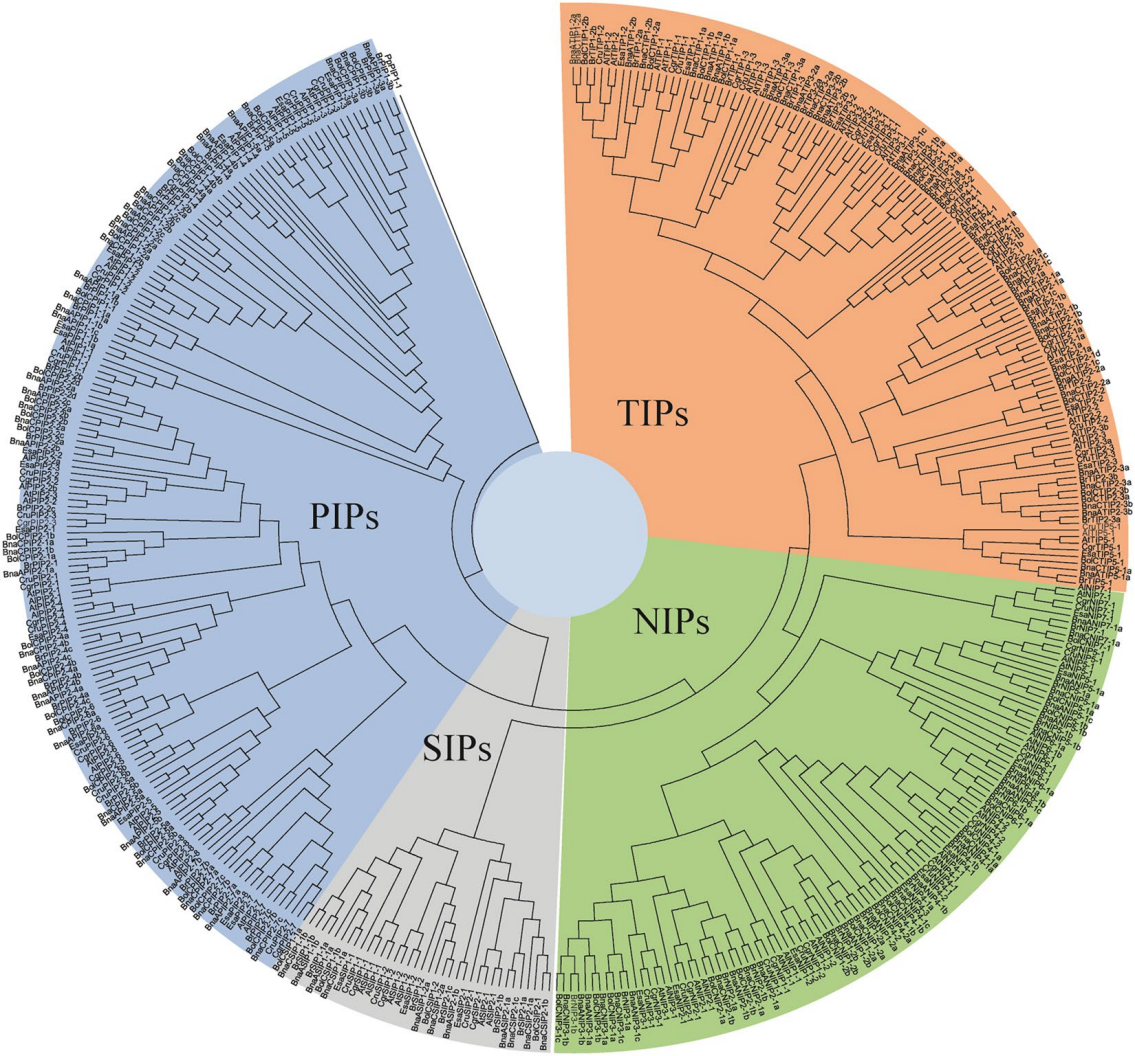
Phylogenetic tree of aquaporins identified in seven Brassicaceae species along with the previously reported aquaporins in *Arabidopsis thaliana*. Aquaporins are denoted with the abbreviation of species name as At -*Arabidopsis thaliana*, Al - *Arabidopsis lyrata*, Cru - *Capsella rubella*, Cgr - *Capsella grandiflora*, Bra- *Brassica rapa*, Bna - *Brassica napus*, Bol - *Brassica oleracea*, Esa - *Eutrema salsugineum*.

Phylogenetic analyses performed on all identified AQPs confirmed the clustering into the four distinct subfamilies PIP, TIP, NIP and SIP (Supplementary Figures S1–S7). Within each subfamily, genes from closely related species tended to cluster together (Fig. 2). The PIP subfamily divided further into two groups, PIP1 and PIP2, with PIP2 having a greater number of genes compared to PIP1 in all seven species (Supplementary Figure 8, Supplementary Table 2). The second largest subfamily, TIP, was divided into five groups. The TIP5 group was initially absent from the results obtained with genome-wide BLASTp search performed in *B*. *oleracea* genome assembly version 2.1. However, when Arabidopsis TIP5 was used as a query to perform a search in NCBI non-redundant database, the results revealed the presence of *B*. *oleracea* TIP5. The NIP subfamily contained seven groups based on the categorization previously reported in *Arabidopsis*. The subfamily SIP clustered into two major groups in all the analyzed genomes (Supplementary Figure 8). The subfamily XIP is entirely missing from the Brassicaceae clad.

### Identification and localization of aquaporins on *B*. *napus* chromosomes

**Figure 3:**
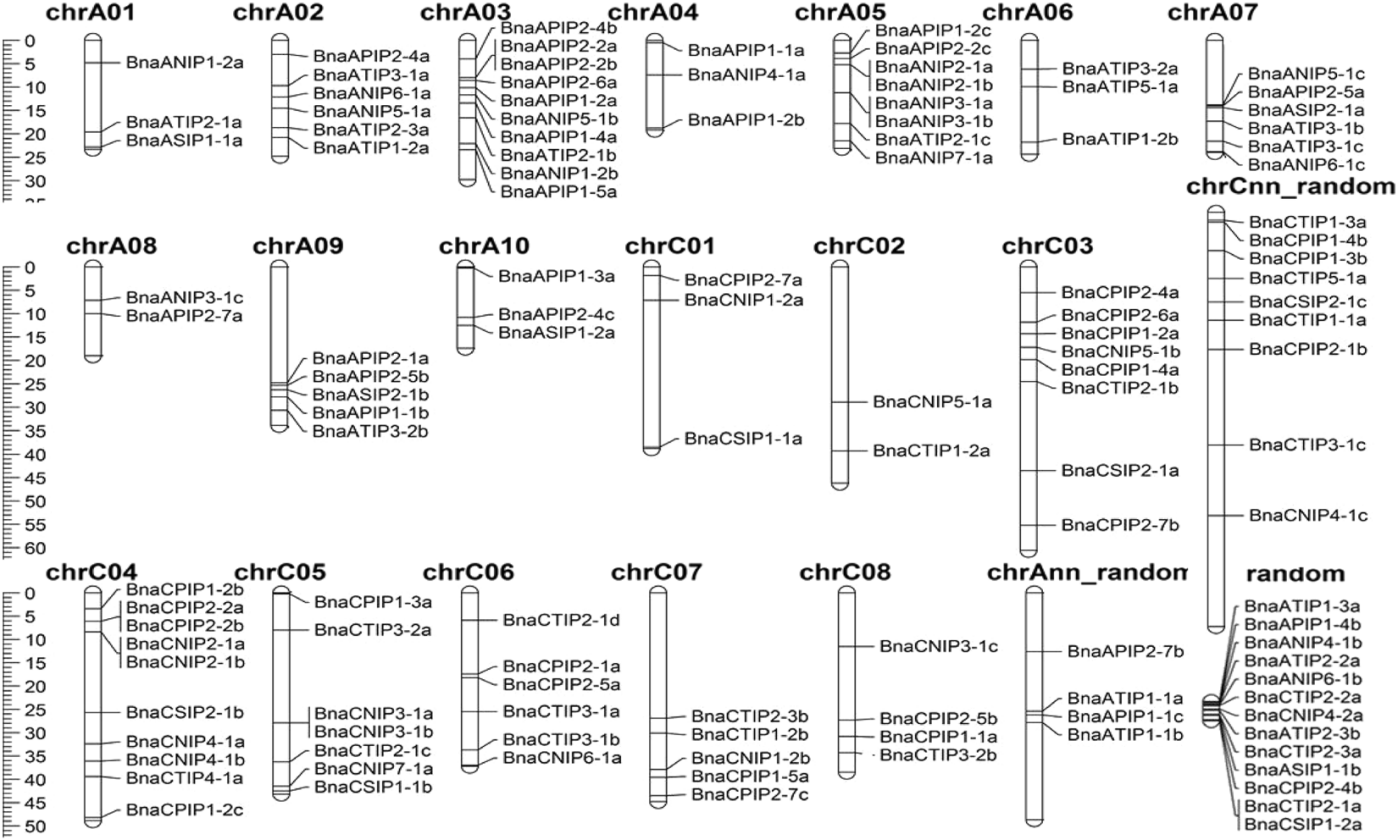
Chromosomal distribution of aquaporin in canola genome (*Brassica napus*). Random denotes the scaffolds unassigned to the chromosome. The scale is in million bases.

Initial results obtained with BLASTp search using a set of known AQPs identified 135 AQPs in the assembly of *B*. *napus* genome. Subsequently, based on protein length and missing transmembrane domains, 15 putative AQP-like proteins were excluded from the list (Supplementary Table 3). Localization of the 120 AQPs to *B*. *napus* chromosomes showed 49 and 45 aquaporins on genome A and C, respectively (Fig. 3). The remaining 26 AQPs were not assigned to any chromosome. However, based on the similarity with the progenitor A and C genomes (*B*. *rapa* and *B*. *oleracea*), unmapped genes were assigned to their respective genomes^40^. In the end, 11 and 15 AQPs were assigned to genome *A random* and *C random*, respectively, for a total of 60 AQPs on each genome. The highest number of AQPs was found on chromosomes A03 and C04, each with ten genes followed by Cnn_random with nine genes, while no AQP was found on chromosomes C9 and C10 (Fig. 3). Details about the chromosomal distribution of AQPs identified in *A*. *lyrata*, *B*. *rapa*, *B*. *oleracea*, *C*. *grandiflora*, *C*. *rubella*, and *E*. *salsugineum* are provided in Supplementary Table 4.

### Gene structure of *B*. *napus* aquaporins

**Figure 4:**
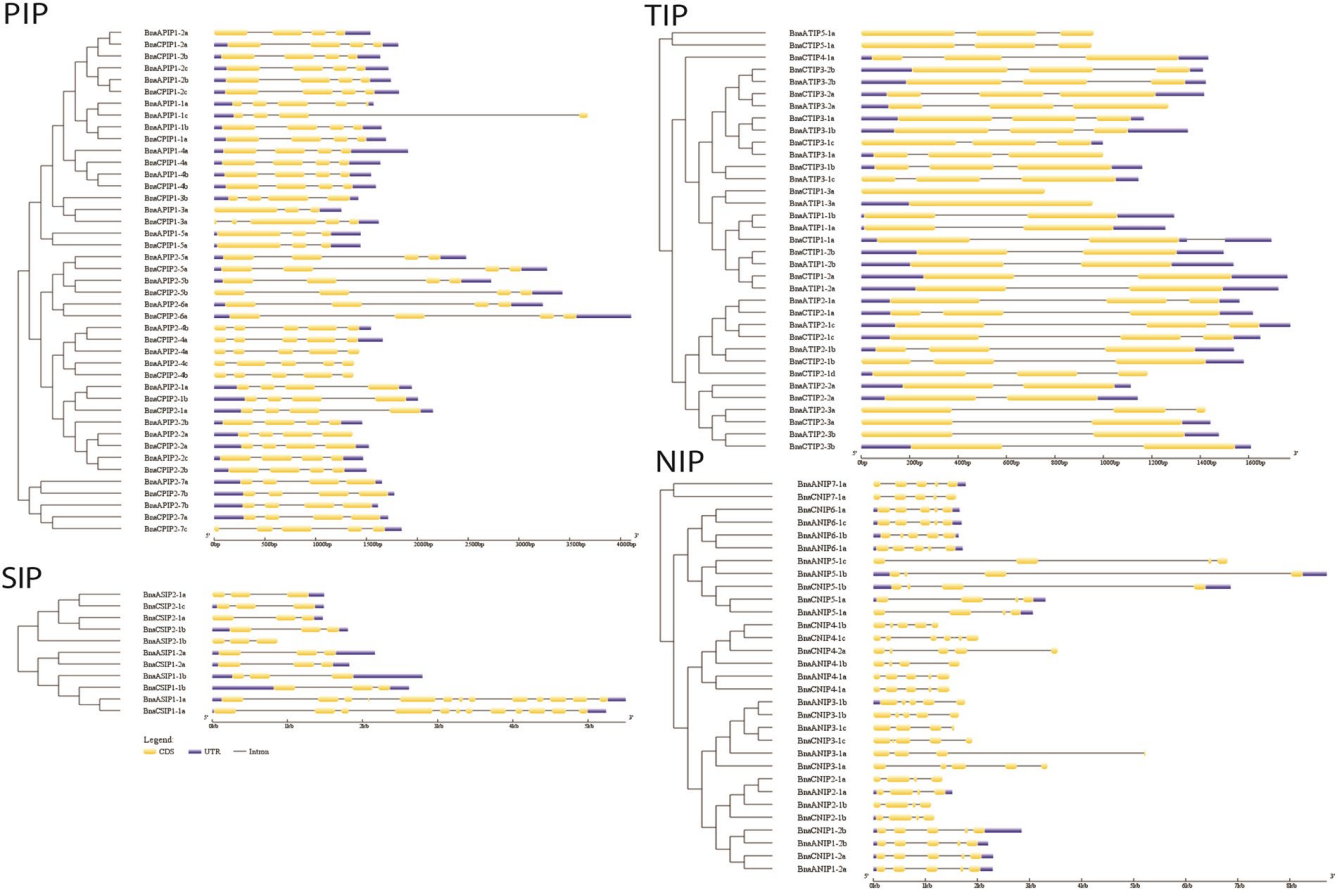
Intron-exon structure and organization of canola aquaporin genes. Aquaporins were categorized into PIP (plasma membrane intrinsic protein), TIP (tonoplast intrinsic proteins), NIP (NOD26-like intrinsic proteins), SIP (small basic intrinsic proteins).

Large variations in intron-exon structure and organization across the four-AQP subfamilies were observed in *B*. *napus* (Fig. 4). In general, the number of introns and the length of introns/exons were found to be conserved within each group of a given AQP subfamily. Overall, the three-intron structure was commonly observed for PIPs with a few exceptions where two or four introns were present. Within the PIP2s, only BnaPIP2–5 and BnaPIP2–6 have longer introns. Another exception was found with BnaAPIP1–1c that has a longer third intron compared to closely related BnaAPIP1–1a and BnaAPIP1–1b. Interestingly, BnaASIP1–1a and BnaCSIP1–1a have 11 and 12 introns per gene respectively, a number much higher than the rest of AQPs in canola (Fig. 4).

### Physical and biochemical properties, predicted transmembrane domains, tertiary structure and subcellular localization of aquaporins

Evaluation of molecular weight (mw) and isoelectric point (pI) revealed differences among the AQP subfamilies in *B*. *napus* (Supplementary Figure 9). Aquaporin sub-families formed distinct clusters when plotted according to their mw and pI. The TIPs, besides having large variation for mw and pI, have relatively lower mw as well as pI compared to other *B*. *napus* AQP subfamilies. The TIPs have an average 26 kD mw and 5.6 pI. Similarly, *B*. *napus* PIPs are also showing a wider range with an average 29.8 kD mw and 7.6 pI. For NIPs, variation is more prominent for mw compared to pI. Most of the NIPs have pI near neutral pH. Compared to other *B*. *napus* AQP, subfamily SIP has much higher pI (average 8.8) and low mw (26.9 kD). Similar trends of variation for mw and pI were observed for AQP subfamilies in the other six Brassicaceae species (Supplementary Table 5). Most of the AQPs showed six transmembrane domains, typical hourglass tertiary structure, and plasma-membrane localization (Supplementary Dataset 1–3).

### Conserved NPA motifs and ar/R selectivity filters in aquaporins

Search performed at CDD database identified two NPA signature motifs in 113 *B*. *napus* AQPs (Table 2). For the other seven AQPs, putative NPA motifs were located based on manual evaluation of protein sequence alignment. Several different amino acid compositions at NPA motifs were observed in *B*. *napus* that included: N-P-V, N-P-S, N-P-L, N-P-C and N-P-T. Amino acid sequence variation at NPA motifs was uniformly observed across AQPs identified in the seven species (Supplementary Tables 6–11). For instance, all the NIP5 homologs identified in seven species have conserved N-P-S at LB and N-P-V at LE. A NPA-NPA spacing of 108 AA was also conserved for all NIP5 proteins. However, there was significant variation in the ar/R SF positions. Another notable example is AtNIP1-1 and AtNIP1-2 both having N-P-A at LB and N-P-G at LE and 116 AA NPA-spacing. AtNIP1-1 has homologs only in *A*. *lyrata*, *C*. *rubella*, and *C*. *grandiflora* but is absent in *Brassica* species and *E*. *salsugineum*, whereas AtNIP1-2 has homologs in all seven species. The entire set of NIP1 homologs has 116 AA NPA-spacing except for BolCNIP1-2b where 111 AA NPA-spacing was observed.

**Table 2:** Details of NPA motifs and ar/R selectivity filters in aquaporins identified in *Brassica napus*.

Gene ID	Aquaporin Name	NPA (LB)	NPA (LE)	H2	H5	LE1	LE2	NPA-NPA Distance
BnaA01g09720D	BnaANIP1-2a	NPA(114)	NPG(233)	W	V	A	R	116
BnaA03g43810D	BnaANIP1-2b	NPA(115)	NPG(234)	W	V	A	R	116
BnaA05g09450D	BnaANIP2-1a	NPA(104)	NPA(223)	V	V	A	R	116
BnaA05g09470D	BnaANIP2-1b	NPA(104)	NPA(223)	V	V	A	R	116
BnaA05g16540D	BnaANIP3-1a	NPA(67)	NPA(186)	W	I	A	R	116
BnaA05g16550D	BnaANIP3-1b	NPA(116)	NPA(211)	W	I	A	R	92
BnaA08g07040D	BnaANIP3-1c	NPA(75)	NPA(194)	W	I	A	R	116
BnaA04g08310D	BnaANIP4-1a	NPA(102)	NPA(214)	W	V	A	R	109
BnaA04g27980D	BnaANIP4-1b	NPA(54)	NPA(166)	W	V	A	R	109
BnaA02g22030D	BnaANIP5-1a	NPS(134)	NPV(245)	A	I	G	R	108
BnaA03g24370D	BnaANIP5-1b	NPS(134)	NPV(245)	A	I	A	R	108
BnaA07g16310D	BnaANIP5-1c	NPS(134)	NPV(245)	A	N	A	R	108
BnaA02g19440D	BnaANIP6-1a	NPA(139)	NPV(250)	A	I	A	R	108
BnaA02g36290D	BnaANIP6-1b	NPA(139)	NPV(250)	A	I	A	R	108
BnaA07g35330D	BnaANIP6-1c	NPA(139)	NPV(250)	A	I	A	R	108
BnaA05g31180D	BnaANIP7-1a	NPS(104)	NPA(216)	A	V	G	R	109
BnaA04g00710D	BnaAPIP1-1a	NPA(53)	NPA(174)	F	H	T	R	118
BnaA09g39170D	BnaAPIP1-1b	NPA(114)	NPA(235)	F	H	T	R	118
BnaAnng23190D	BnaAPIP1-1c	NPA(34)	NPA(155)	F	H	T	R	118
BnaA03g21210D	BnaAPIP1-2a	NPA(114)	NPA(235)	F	H	T	R	118
BnaA04g26560D	BnaAPIP1-2b	NPA(114)	NPA(235)	F	H	T	R	118
BnaA05g05230D	BnaAPIP1-2c	NPA(114)	NPA(235)	F	H	T	R	118
BnaA10g00360D	BnaAPIP1-3a	NPA(114)	NPA(235)	F	H	T	R	118
BnaA03g27130D	BnaAPIP1-4a	NPA(114)	NPA(235)	F	H	T	R	118
BnaA09g51960D	BnaAPIP1-4b	NPA(114)	NPA(235)	F	H	T	R	118
BnaA03g45950D	BnaAPIP1-5a	NPA(115)	NPA(236)	F	H	T	R	118
BnaA09g33720D	BnaAPIP2-1a	NPA(107)	NPA(228)	F	H	T	R	118
BnaA03g17020D	BnaAPIP2-2a	NPA(105)	NPA(226)	F	H	T	R	118
BnaA03g17030D	BnaAPIP2-2b	NPA(105)	NPA(226)	F	H	T	R	118
BnaA05g07300D	BnaAPIP2-2c	NPA(105)	NPA(251)	F	H	T	R	143
BnaA02g06180D	BnaAPIP2-4a	NPA(80)	NPA(201)	F	H	T	R	118
BnaA03g08820D	BnaAPIP2-4b	NPA(82)	NPA(203)	F	H	T	R	118
BnaA10g13480D	BnaAPIP2-4c	NPA(82)	NPA(203)	F	H	T	R	118
BnaA07g16510D	BnaAPIP2-5a	NPA(106)	NPA(227)	F	H	T	R	118
BnaA09g34600D	BnaAPIP2-5b	NPA(106)	NPA(227)	F	H	T	R	118
BnaA03g18300D	BnaAPIP2-6a	NPA(105)	NPA(226)	F	H	T	R	118
BnaA08g10860D	BnaAPIP2-7a	NPA(101)	NPA(222)	F	H	T	R	118
BnaAnng11630D	BnaAPIP2-7b	NPA(101)	NPA(222)	F	H	T	R	118
BnaA07g17050D	BnaASIP2-1a	NPL(69)	NPA(180)	S	H	G	A	108
BnaA09g36250D	BnaASIP2-1b	NPV(69)	NPA(177)	S	H	G	A	105
BnaAnng22640D	BnaATIP1-1a	NPA(55)	NPA(169)	H	I	A	V	111
BnaAnng24130D	BnaATIP1-1b	NPA(55)	NPA(169)	H	I	A	V	111
BnaA02g28130D	BnaATIP1-2a	NPA(86)	NPA(200)	H	I	A	V	111
BnaA06g32840D	BnaATIP1-2b	NPA(86)	NPA(200)	H	I	A	V	111
BnaA09g51590D	BnaATIP1-3a	NPA(85)	NPA(199)	H	I	A	V	111
BnaA01g28120D	BnaATIP2-1a	NPA(83)	NPA(197)	H	I	G	R	111
BnaA03g34110D	BnaATIP2-1b	NPA(83)	NPA(197)	H	I	G	R	111
BnaA05g23460D	BnaATIP2-1c	NPA(83)	NPA(197)	H	I	G	R	111
BnaA01g35340D	BnaATIP2-2a	NPA(83)	NPA(197)	H	I	G	R	111
BnaA02g25440D	BnaATIP2-3a	NPA(43)	NPA(157)	H	I	G	R	111
BnaA06g40020D	BnaATIP2-3b	NPA(83)	NPA(197)	H	I	G	R	111
BnaA02g16380D	BnaATIP3-1a	NPA(93)	NPA(207)	H	I	A	R	111
BnaA07g22790D	BnaATIP3-1b	NPA(93)	NPA(207)	H	I	A	R	111
BnaA07g30640D	BnaATIP3-1c	NPA(93)	NPA(207)	H	I	A	R	111
BnaA06g12030D	BnaATIP3-2a	NPA(93)	NPA(207)	H	M	A	R	111
BnaA09g44820D	BnaATIP3-2b	NPA(93)	NPA(207)	H	M	A	R	111
BnaA06g17390D	BnaATIP5-1a	NPA(87)	NPA(200)	N	V	G	C	110
BnaC01g11410D	BnaCNIP1-2a	NPA(114)	NPG(233)	W	V	A	R	116
BnaC07g35550D	BnaCNIP1-2b	NPA(115)	NPG(234)	W	V	A	R	116
BnaC04g10820D	BnaCNIP2-1a	NPA(104)	NPA(223)	V	V	A	R	116
BnaC04g10830D	BnaCNIP2-1b	NPA(104)	NPA(223)	V	V	A	R	116
BnaC05g29140D	BnaCNIP3-1a	NPA(99)	NPA(218)	W	I	A	R	116
BnaC05g29150D	BnaCNIP3-1b	NPA(116)	NPA(211)	W	I	A	R	92
BnaC08g07910D	BnaCNIP3-1c	NPA(102)	NPA(232)	W	I	A	R	127
BnaC04g30520D	BnaCNIP4-1a	NPA(102)	NPA(214)	W	V	A	R	109
BnaC04g34450D	BnaCNIP4-1b	NPA(102)	NPA(214)	W	V	A	R	109
BnaC06g42210D	BnaCNIP4-2a	NPA(107)	NPA(219)	W	V	A	R	109
BnaC02g29210D	BnaCNIP5-1a	NPS(134)	NPV(245)	A	I	G	R	108
BnaC03g28980D	BnaCNIP5-1b	NPS(134)	NPV(245)	A	I	A	R	108
BnaC06g40240D	BnaCNIP6-1a	NPA(139)	NPV(250)	A	I	A	R	108
BnaC05g45720D	BnaCNIP7-1a	NPS(104)	NPA(216)	A	V	G	R	109
BnaC08g31360D	BnaCPIP1-1a	NPA(114)	NPA(235)	F	H	T	R	118
BnaC03g25510D	BnaCPIP1-2a	NPA(114)	NPA(235)	F	H	T	R	118
BnaC04g04640D	BnaCPIP1-2b	NPA(114)	NPA(235)	F	H	T	R	118
BnaC04g50590D	BnaCPIP1-2c	NPA(114)	NPA(235)	F	H	T	R	118
BnaC05g00440D	BnaCPIP1-3a	NPA(152)	NPA(273)	F	H	T	R	118
BnaCnng08780D	BnaCPIP1-3b	NPA(114)	NPA(235)	F	H	T	R	118
BnaC03g32130D	BnaCPIP1-4a	NPA(114)	NPA(235)	F	H	T	R	118
BnaCnng02360D	BnaCPIP1-4b	NPA(114)	NPA(235)	F	H	T	R	118
BnaC07g38190D	BnaCPIP1-5a	NPA(115)	NPA(236)	F	H	T	R	118
BnaC06g14590D	BnaCPIP2-1a	NPA(107)	NPA(228)	F	H	T	R	118
BnaCnng31040D	BnaCPIP2-1b	NPA(107)	NPA(228)	F	H	T	R	118
BnaC04g08090D	BnaCPIP2-2a	NPA(105)	NPA(226)	F	H	T	R	118
BnaC04g08100D	BnaCPIP2-2b	NPA(105)	NPA(226)	F	H	T	R	118
BnaC03g11160D	BnaCPIP2-4a	NPA(82)	NPA(203)	F	H	T	R	118
BnaC09g53920D	BnaCPIP2-4b	NPA(82)	NPA(203)	F	H	T	R	118
BnaC06g15450D	BnaCPIP2-5a	NPA(106)	NPA(227)	F	H	T	R	118
BnaC08g25570D	BnaCPIP2-5b	NPA(106)	NPA(227)	F	H	T	R	118
BnaC03g21800D	BnaCPIP2-6a	NPA(105)	NPA(226)	F	H	T	R	118
BnaC01g03410D	BnaCPIP2-7a	NPA(101)	NPA(222)	F	H	T	R	118
BnaC03g65520D	BnaCPIP2-7b	NPA(101)	NPA(222)	F	H	T	R	118
BnaC07g45370D	BnaCPIP2-7c	NPA(74)	NPA(195)	F	H	T	R	118
BnaC03g54990D	BnaCSIP2-1a	NPL(69)	NPA(180)	S	H	G	A	108
BnaC04g24760D	BnaCSIP2-1b	NPL(69)	NPA(180)	S	H	G	A	108
BnaCnng20470D	BnaCSIP2-1c	NPL(69)	NPA(180)	S	H	G	A	108
BnaCnng24720D	BnaCTIP1-1a	NPA(85)	NPA(199)	H	I	A	V	111
BnaC02g36210D	BnaCTIP1-2a	NPA(86)	NPA(200)	H	I	A	V	111
BnaC07g23630D	BnaCTIP1-2b	NPA(86)	NPA(200)	H	I	A	V	111
BnaCnng01570D	BnaCTIP1-3a	NPA(85)	NPA(199)	H	I	A	V	111
BnaC01g44580D	BnaCTIP2-1a	NPA(83)	NPA(197)	H	I	G	R	111
BnaC03g39560D	BnaCTIP2-1b	NPA(109)	NPA(223)	H	I	G	R	111
BnaC05g37160D	BnaCTIP2-1c	NPA(83)	NPA(197)	H	I	G	R	111
BnaC06g05270D	BnaCTIP2-1d	NPA(82)	NPA(196)	H	I	G	R	111
BnaC01g41690D	BnaCTIP2-2a	NPA(83)	NPA(197)	H	I	G	R	111
BnaC02g46870D	BnaCTIP2-3a	NPA(83)	NPA(197)	H	I	G	R	111
BnaC07g20220D	BnaCTIP2-3b	NPA(83)	NPA(197)	H	I	G	R	111
BnaC06g23750D	BnaCTIP3-1a	NPA(93)	NPA(207)	H	I	A	R	111
BnaC06g34100D	BnaCTIP3-1b	NPA(93)	NPA(207)	H	I	A	R	111
BnaCnng50290D	BnaCTIP3-1c	NPA(93)	NPA(207)	H	I	A	R	111
BnaC05g13770D	BnaCTIP3-2a	NPA(93)	NPA(207)	H	M	A	R	111
BnaC08g37510D	BnaCTIP3-2b	NPA(93)	NPA(207)	H	M	A	R	111
BnaC04g38040D	BnaCTIP4-1a	NPA(79)	NPA(193)	A	I	A	R	111
BnaCnng15220D	BnaCTIP5-1a	NPA(87)	NPA(200)	N	V	G	C	110
BnaA01g33700D	BnaASIP1-1a	NPT(69)*	NPA(184)*	T	V	P	I	112
BnaA05g37440D	BnaASIP1-1b	NPT(69)*	NPA(184)*	T	V	P	I	112
BnaA10g16540D	BnaASIP1-2a	NPC(72)*	NPA(188)*	V	F	P	I	113
BnaCnng65250D	BnaCNIP4-1c	NP(107)*	—	P	—	—	—	—
BnaC01g40230D	BnaCSIP1-1a	NPT(71)*	NPA(186)*	T	V	P	I	112
BnaC05g47470D	BnaCSIP1-1b	NPT(69)*	NPA(184)*	T	V	P	I	112
BnaC09g54320D	BnaCSIP1-2a	NPC(72)*	NPA(188)*	V	F	P	I	113

NPA motifs were identified using CDD search at NCBI. The NPA motifs with an asterisk (*) initially failed to be identified with CDD search and were then identified with manual evaluation of protein sequence alignments.

The AAs at four ar/R SF positions are highly conserved for *B*. *napus* PIPs and TIPs, while more variations were observed with NIPs and SIPs. The entire set of 43 PIPs identified in *B*. *napus* has a F-H-T-R SF in H2, H5, LE^1^ and LE^2^. In the TIP subfamily, there is variation in ar/R SF composition across the groups. All members of *B*. *napus* TIP1s harbor H-I-A-V, TIP2s H-I-G-R, TIP4 A-I-A-R, and TIP5 N-V-G-C. TIP3s have variations at ar/R SF H5 position and contain an AA composition H-[I/M]-A-R. A distinct or common composition for ar/R SF was not observed within *B*. *napus* NIP groups. The *B*. *napus* SIP1 has two different ar/R SFs: T-V-P-I and V-F-P-I. All the SIP2s have S-H-G-A. Similar to the NPA motifs, the ar/R SFs are also conserved across all *Arabidopsis* AQP homologs.

**Figure 5:**
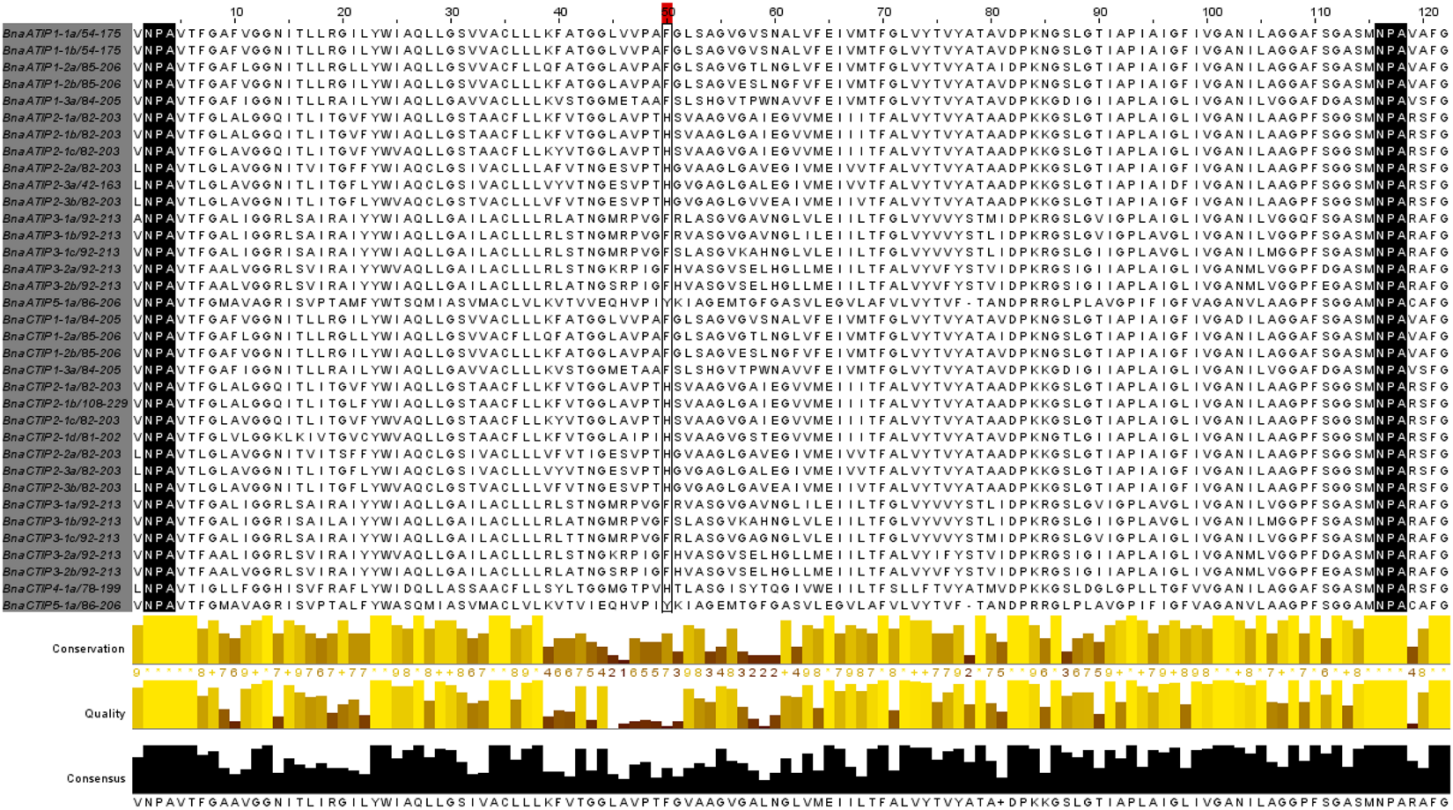
Alignment of *Brassica napus* TIPs showing 5^th^ selectivity filter newly identified in Arabidopsis AtTIP2-1.

A residue in Loop C (Lc) contributing to ar/R selectivity filter newly identified in Arabidopsis AtTIP2-1^41^ was found to be conserved across all the TIPs identified in *B*. *napus* and six other species analyzed in the present study. The protein sequence alignment of TIPs from all the seven species revealed that TIP1s and TIP3s had an F residue whereas TIP2s and TIP4s had H, and TIP5s had Y at Lc ar/R SF position (Fig. 5). Similarly, the Lc ar/R SF was found to be conserved across all other subfamilies.

### Absence of NIP-IIIs in Brassicaceae and Si accumulation in canola

A notable absence of specific AQPs from Brassicaceae was the group NIP2 with G-S-G-R SF pore (NIP-III), known to allow Si permeability, and often referred to as Si influx transporter or Lsi1 (Table 2). On the other hand, our genomic analysis revealed the presence of a putative Si efflux transporters (Lsi2) (Supplementary information 2). When canola plants were tested for their ability to absorb Si, results clearly showed that they did not accumulate Si above the background level (Supplementary Figure 14).

### AQP expression profiling across different conditions and tissues

A total of 74 sequenced RNA-seq libraries representing five independent experiments were used to evaluate the expression profiling of *B*. *napus* AQPs (Supplementary Dataset 4 and 5). Out of 120 AQPs, 104 were found to be expressed with a criterion of >2 RPKM at least in a single library out of 74 used to claim expression of AQPs (Supplementary Dataset 4 and 5). The 16 unexpressed AQPs include 10 NIPs, four TIPs and two SIPs. On the other hand, expression was observed for all PIPs. Overall PIPs and TIPs had much higher levels of expression with an average of 154 and 187 RPKM, respectively. Comparatively, NIPs and SIPs have lower levels of expression with averages of 13 and 35, respectively. However, large variations across tissues and experimental conditions were observed for AQPs in all four subfamilies (Supplementary Dataset 4 and 5).

### Expression of *B*. *napus* aquaporins in seed tissues

**Figure 6:**
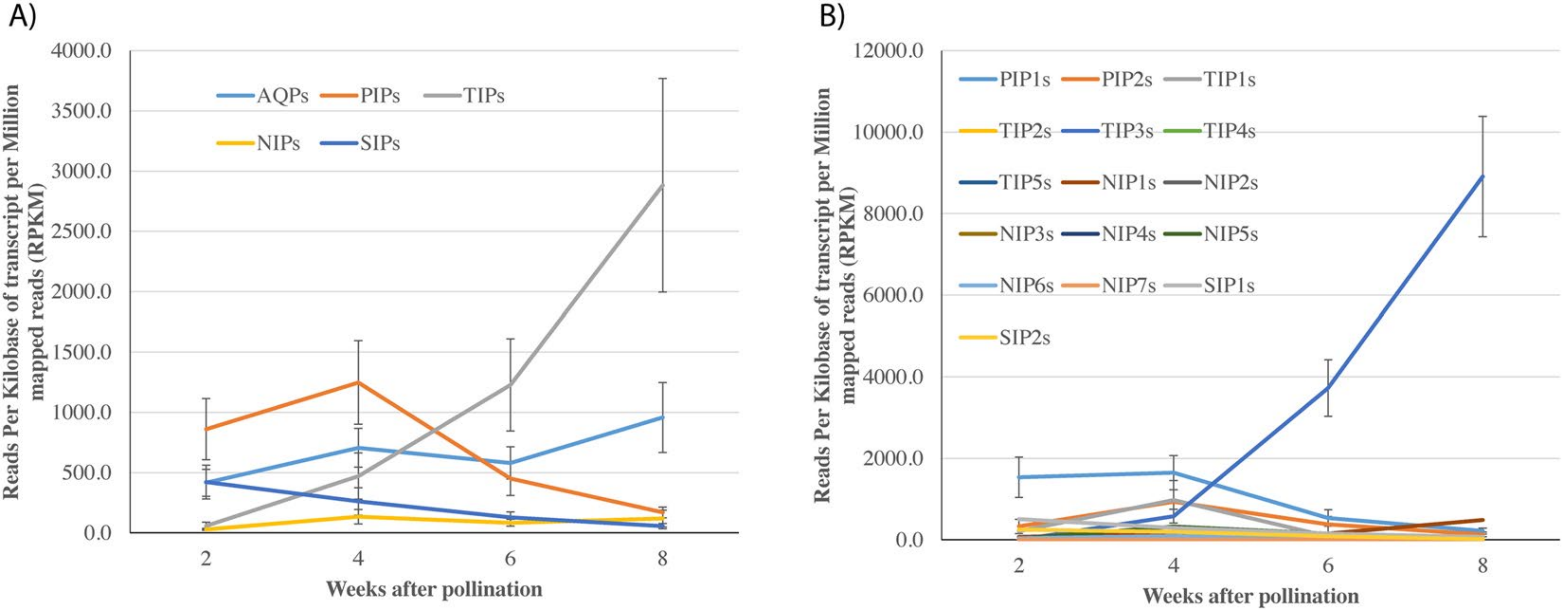
Expression of *Brassica napus* AQP in developing seeds. RNA-seq expression data available (Bioproject PRJNA311067) for *B*. *napus* seed at 2, 4, 6, and 8 weeks after pollination was used for AQP expression profiling. Graphs are showing expression level in term of average reads per kilobases of transcript per million mapped reads (RPKM) for (**A**) four AQP subfamilies namely PIP, Plasma membrane intrinsic protein; TIP, Tonoplast intrinsic protein; NIP, Noduline-26 like intrinsic protein; SIP, Small intrinsic protein; and (**B**) groups in each subfamily. Bars represent standard error from the mean for all values within a subfamily or a group. Detailed data are provided in Supplementary Dataset 4.

The RNA-seq data from Bioproject PRJNA311067 (Supplementary Table 1) were used to study expression of AQPs in seed tissues. The overall AQP activity in developing seed tissues harvested at 2, 4, 6, and 8 weeks after pollination (WAP) increased over two-fold from 415 (2 WAP) to 956 RPKM (8 WAP). The PIP and TIP subfamilies had particularly high levels of expression compared to SIPs and NIPs (Fig. 6A). Expression of PIPs, TIPs and NIPs initially increased to 4 WAP whereas SIPs expression steadily decreased over time. At four WAP, expression of PIPs went down drastically, but the expression of TIPs kept on increasing until 8 WAP (Fig. 6A). However, very high variations were observed within the subfamily, which prompted further analyses into different groups in each subfamily.

Among the different groups, PIP1s and TIP3s have the highest level of expression in seed tissue (Fig. 6B). During early development at 2 WAP, PIP1s had a fairly high level of expression whereas TIP3s had a low level. Over time as seeds matured, expression of PIP1s decreased in contrast to expression of TIP3s that increased several folds during the late stages of seed development. In the developing seeds, 86 out of the 120 AQPs were expressed with average > 2 RPKM. The remaining 34 AQPs with low or no expression in seed tissue mostly included NIPs (15) and TIPs (13). Hierarchical clustering of the AQPs expressed in the seeds showed two major clusters based on the expression pattern (Supplementary Figure 10). All TIP3 genes clustered together and showed a similar expression pattern while the other AQPs formed a separate cluster. Co-expression analysis revealed a negative correlation of TIP3s with most of the other subfamilies (Supplementary Figure 11). Similarly, a positive correlation between the PIP1s and PIP2s, and among TIP2s was observed with co-expression network analysis.

### Expression of *B*. *napus* aquaporins under drought conditions

The RNA-seq data (Bioproject PRJNA256233) analysis revealed an interesting contrast for AQP expression between leaves and roots of *B*. *napus* under drought conditions. The overall expression of AQPs increased over two-fold in leaves under drought conditions while it decreased significantly in roots (Supplementary Figure 12A). This change in expression was more prominent in PIPs and TIPs (Supplementary Figure 12A). In the PIP subfamily, both PIP1s and PIP2s were particularly highly expressed. However, in the TIP subfamily, only members of the TIP1 group had a significantly higher level of expression in both tissues and under both control and drought treatments (Supplementary Figure 12B). With a cut-off >2 log2 fold change and average 2 RPKM, 16 AQPs were identified as differentially expressed genes (DEGs) in leaves. All of these genes were up-regulated under the drought treatment. Interestingly, all 16 DEGs belong to PIP and TIP subfamilies.

### Expression of *B*. *napus* aquaporins under biotic stress

**Figure 7:**
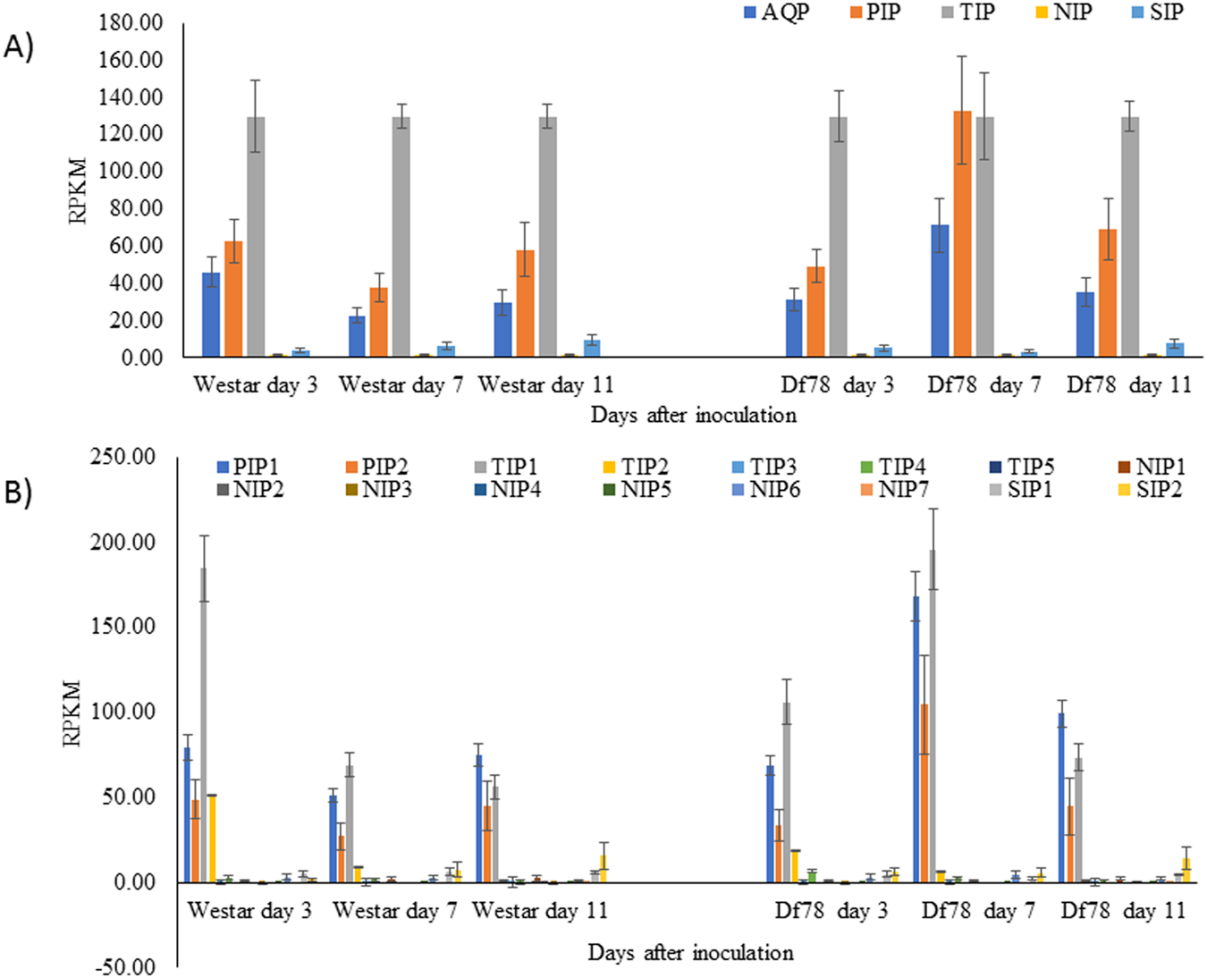
Expression of *Brassica napus* AQP in susceptible and resistant genotypes after inoculation with *Leptosphaeria maculans* retrieved from Bioproject PRJNA311316. Graphs are showing expression level in term of average reads per kilobases of transcript per million mapped reads (RPKM) for (**A**) four AQP subfamilies namely PIP, Plasma membrane intrinsic protein; TIP, Tonoplast intrinsic protein; NIP, Noduline-26 like intrinsic protein; SIP, Small intrinsic protein; and (**B**) groups in the each subfamily. Bars represent standard error from the mean for all values within a subfamily or a group out of three biological replicates. Detailed data are provided in Supplementary Dataset 4.

Expression of *B*. *napus* AQPs under biotic stress was evaluated using the RNA-seq transcriptome data (Bioproject PRJNA311316) available for the hemibiotrophic fungal pathogen *Leptosphaeria maculans* inoculated to resistant (LepR1) genotype DF78 and the susceptible genotype Westar. In the resistant genotype, the number of differential expressed AQPs was much higher compared to the susceptible genotype. More specifically, PIPs were particularly highly expressed in the resistant genotype (Fig. 7A). Most of the PIP1s and PIP2s were up-regulated during the early stage, but expression was reduced drastically during the later stage of disease development in resistant genotype Df78. In the case of TIPs, only TIP1s has significantly higher expression. Interestingly, in susceptible genotype Westar, TIP1 expression was higher at 3 dpi but receded during later stages (Fig. 7B). In contrast, the highest level of TIP1 expression was observed at 7 dpi in Df78 genotype. Among the NIPs and SIPs, NIP6s and SIP2s had higher expression levels.

**Figure 8:**
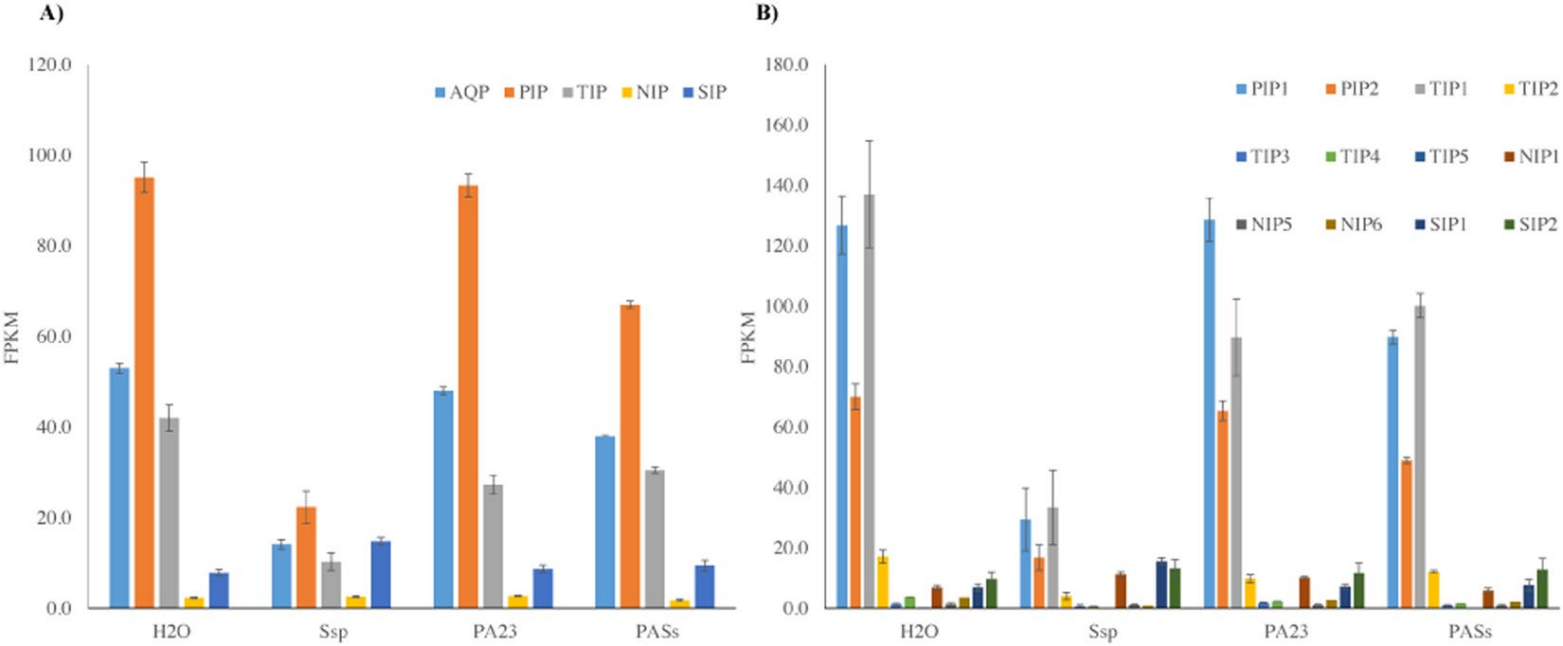
Expression of *Brassica napus* AQP in mature leaf inoculated with *Sclerotinia sclerotiorum* (Ssp), biocontrol agent *Pseudomonas chlororaphis* (PA23) and combined both together retrieved from Bioproject PRJNA331148. Graphs are showing expression level in term of average reads per kilobases of transcript per million mapped reads (RPKM) for (**A**) four AQP subfamilies namely PIP, Plasma membrane intrinsic protein; TIP, Tonoplast intrinsic protein; NIP, Noduline-26 like intrinsic protein; SIP, Small intrinsic protein; and (**B**) groups in each subfamily. Bars represent standard error from the mean for all values within a subfamily or a group out of three biological replicates. Detailed data are provided in Supplementary Dataset 4.

Another set of RNA-seq data (Bioproject PRJNA331148) publicly available for *B*. *napus* infected with the necrotrophic fungus *Sclerotinia sclerotiorum* (Ssp) and the biocontrol agent *Pseudomonas chlororaphis* (PA23) was used to further investigate AQP expression dynamics. Comparison of AQP expression in mature leaf tissue inoculated with water, Ssp ascospores, PA23, or both PA23 and Ssp (PASs), revealed much lower expression levels of AQPs following Ssp infection (Fig. 8A). By contrast, PIPs and TIPs had higher levels of expression in control and PA23-inoculated samples. In the case of samples inoculated with both PA23 and Ssp, expression of PIPs and TIPs was slightly lower compared to controls but still much higher than in the samples infected with Ssp alone (Fig. 8A). Overall, PIP1s, PIP2s, TIP1s and TIP2s were the most expressed AQPs (Fig. 8B). Interestingly, SIPs contrasted with the other groups both in levels and patterns of expression according to treatments (Fig. 8B). A total of three clusters were formed with hierarchical clustering (Supplementary Figure 13). Cluster 1 grouped AQPs with the highest expression in control, followed by PA23, and PASs, and lowest in Ssp; Cluster 2 represented AQPs with high expression in control and PA23, slightly lower in PASs, and much lower in Ssp; finally, Cluster 3 grouped AQPs with low overall expression.

### Aquaporin expression with different levels of gene dosage

**Figure 9:**
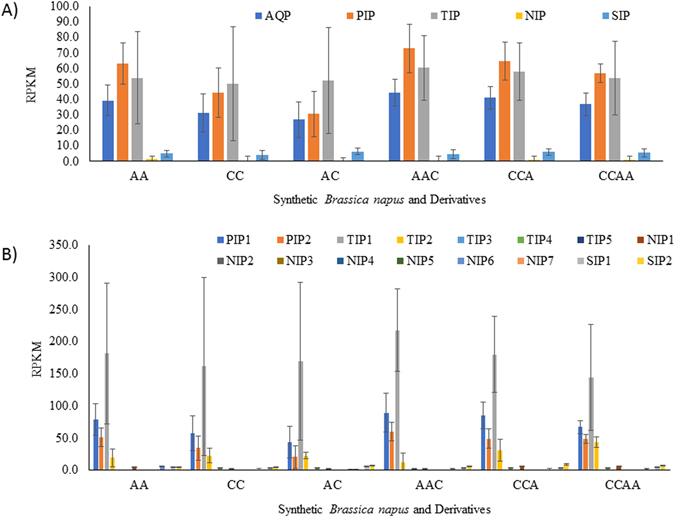
Expression of aquaporins (AQPs) in *B*. *rapa* (A genome, 2n = 20) and *B*. *oleracea* (B genome, 2n = 18) parents and their synthesized progenies with different copies of sub-genomes (AC, AAC, CCA, CCAA) retrieved from Bioproject PRJNA322687. Graphs are showing expression level in term of average reads per kilobases of transcript per million mapped reads (RPKM) for (**A**) four AQP subfamilies namely PIP, Plasma membrane intrinsic protein; TIP, Tonoplast intrinsic protein; NIP, Noduline-26 like intrinsic protein; SIP, Small intrinsic protein; and (**B**) groups in each subfamily. RNA-seq expression data was obtained from Tan *et al*. (2016). Bars represent standard error from the mean for all values within a subfamily or a group out of six samples from two biological replicates. Detailed data are provided in Supplementary Dataset 5.

The transcriptome profiling data (Bioproject PRJNA322687) available for *B*. *rapa* (A genome, 2n = 20) and *B*. *oleracea* (B genome, 2n = 18) parents and their synthesized progenies with different copies of sub-genomes (AC, AAC, CCA, CCAA) were used to evaluate gene dosages effect on AQP expression^18^. Overall triploid hybrid CCA (2n = 28) and AAC (2n = 29) showed significantly higher AQP expression compared to diploid parental lines, *B*. *napus* (AC, 2n = 19) and allotetraploid CCAA (2n = 38) (Fig. 9A). The AA genotype has higher expression than the CC genotype and this was also consistent with the triploids where AAC had greater AQP expression than CCA. Interestingly, the AQP expression in CCAA was much higher than in AC despite having equivalent fractions of A and C genomes. The expression of PIP and TIP subfamily was more affected by ploidy levels and by genome combinations (Fig. 9A).

Comparison within the groups across all the subfamilies showed overall similar trends, with the highest expression in AA followed by CC and then AC (Fig. 9B). Similarly, the highest level of expression was observed in AAC followed by CCA and CCAA. An exception to this common trend was found in TIP2, SIP1 and SIP2. In the case of SIPs, the descending order for expression level was AC, AA and then CC. In triploids and tetraploids, the descending order for expression of SIP1 was CCAA, AAC and CCA but SIP2 had higher expression in CCAA followed by CCA (Fig. 9B).

Dosage-dependent genes were identified by Pearson’s correlation analysis performed between the expression data and genome composition. For instance, AA, AC, AAC, CCA, and CCAA have A genome composition as 1, 0.5, 0.66, 0.33, and 0.5 respectively. About 98% of the AQPs showed a positive correlation with the genome composition (Supplementary Dataset 5). Only 30% of expressed AQPs were significantly correlated with genome composition (p < 0.01, Supplementary Dataset 5). The AQPs assigned to A genome were more dosage dependent (p < 0.01) compared to AQPs on C genome. PIPs, including both PIP1s and PIP2s, showed more dosage dependent patterns of expression. In the TIP subfamily, only TIP1s, and in SIPs only SIP1s showed gene dosage dependency. For their part, NIPs showed mostly dosage-independent expression.

## Discussion

In the present study, genome-wide identification and characterization of AQPs were performed in Brassicaceae species to understand their evolution within this important group of plants and to further analyze their possible molecular role, particularly in *B*. *napus*. The first genome-wide AQP study was carried out in *Arabidopsis* and served as a reference for the identification of homologs in many other crop plants^15, 20, 21^. Being a member of the Brassicaceae family, *Arabidopsis* provides a close phylogenetic resource to infer information for economically important crop species in the family. To perform the comparative analysis, we have exploited genomic information available for species from two closely related tribes: the Camelineae including *A*. *thaliana*, *A*. *lyrata*, *C*. *rubella*, *C*. *grandiflora*, and the Calepineae including *B*. *rapa*, *B*. *napus*, *B*. *oleracea* and *E*. *salsugineum*. These genomes together with available transcriptomic resources have made it possible to understand many aspects including genomic distribution, structural and functional conservation, phylogenetic relations and the expression dynamics across different tissues and environmental conditions.

### Loss of XIPs and silicon transporter NIPs in Brassicaceae

Genomic distribution of AQPs have been studied in several plant species including monocots, dicots and primitive species like *Physcomitrella patens*, *Picea abies*, *Selaginella* moellendorffii^12^. The primitive species have two more AQP subfamilies: GlpF-like intrinsic protein (GIPs) and hybrid intrinsic protein (HIPs), along with the five commonly observed families in higher plants. As in other higher plants, the two subfamilies HIP and GIPs are absent in Brassicaceae. The XIP family, common to most of the dicots, are also missing in all studied species inferring a recent loss during evolution (Supplementary Table 12). The XIPs have been reported to transport several solutes like glycerol, H_2_O_2_, copper, boric acid, and arsenic in tobacco^42^. Similarly, Noronha, *et al*.^43^ have reported a role for XIPs in drought stress and also in the transport of glycerol, hydrogen peroxide, heavy metals in grapevine. The loss of XIPs in Brassicaceae suggest that the functions of XIPs were shared with other AQPs or were of lesser importance.

Another interesting feature in Brassicaceae was the absence of NIPs with G-S-G-R SF (NIP-III) known to be essential for silicon (Si) transport. Recent evidence has linked the presence of those NIPs with, Si accumulation and benefits from Si fertilization in many plant families. Brassicaceae now join Solanaceae among families that are poor Si accumulators on the basis of AQP profile (Supplementary Figure 14)^12^. This is further supported by our phenotyping results showing clearly that canola could not accumulate Si, and previous reports^44, 45^ that showed that Arabidopsis was a poor Si accumulator. Interestingly, putative Si-efflux transporters were identified in all seven species, which would suggest that the presence or absence of Si permeable NIP-IIIs is the critical factor in determining the ability of a plant to absorb Si.

### Recent genome duplications attributed to high number of AQPs in *Brassicaceae* species

In spite of important variations in genome size, a comparable number of AQPs were identified among species in the Camelineae tribe. For instance, the *A*. *lyrata* genome is almost double the size of Arabidopsis and yet has an overall similar number of AQPs^46^. Genome size variation in these closely related species is attributed to large-scale rearrangements, including many small deletions in the non-coding region and transposons^46^. On the other hand, in the four Calepineae species, variations observed in the total number of AQPs are attributed more to variations in genome size resulting from polyploidization. Overall the number of AQPs were well correlated with genome size and the total number of genes, an observation consequent with the relatively recent duplication events in these species^40^. Interestingly, the observed correlation contradicts earlier reports where a negative correlation was observed between the percentage of protein-coding genes and the genome size^47^. For instance, the *B*. *napus* genome, known to be formed by recent allopolyploidy between *B*. *rapa* (AA) and *B*. *oleracea* (CC), has a number of AQPs equal to the total number of AQPs found in both species. The number of AQPs in each subfamily corresponding to AA and CC genome of *B*. *napus* also correlates well with the respective ancestral genomes (*B*. *oleracea* and *B*. *rapa*). Most of the *B*. *napus* AQPs identified here were mapped on assembled chromosomes except for 26 AQPs found in random scaffold regions of perturbed synteny (see Fig. 3). This could lead to a possible change in total number of AQPs with improved genome assembly in the future.

### Gene structure, biochemical and physical properties, and conserved domains in aquaporins

Intron-exon organization is necessary to confirm the polyphyletic origin of true gene homologs and paralogs and to understand the functionality of the gene^43, 48^. The intron-exon organization was found to be conserved within each AQP subfamily and more prominently among the genes paired phylogenetically. Such conserved intron-exon organization of AQP subfamilies has also been observed in several plant genomes including chickpea, soybean, rice, and *Arabidopsis* ^16, 21^. Similarly, biochemical and physical properties were also found to be well aligned with the phylogenetic distribution. Molecular weight and pI are also indicative of the subcellular localization. Earlier, proteomic analysis revealed relatively lower pI (6.69) for vacuolar proteins as compared to all other proteins (pI 7.40) in the *Arabidopsis* genome^49^. This is consistent with the lower pI predicted in the present study for the TIPs (pI 5.6).

The NPA signature motif observed in AQPs are known to have a role in the regulation of solute transport and also in the localization of the protein in the membrane^50, 51^. In the present study, only the third AA position of the NPA motif showed variation in some of the NIPs and SIPs. This suggests a lesser selectivity role for the third amino acid in these subfamilies. A mutagenesis study performed with AtNIP5;1 showed no significant effect on the transport of solutes (Si and arsenate) by changing the original NPS and NPV with the NPA motif ^52^. However, mutagenesis experiments performed with Si transporter AQPs revealed that the conserved NPA-NPA spacing played a crucial factor in substrate selectivity^12^. This might be similarly critical for other AQP subfamilies since the spacing between NPA motifs were found to be conserved for homologs across the seven species. In the case of Ar/R SF, apart from having considerable variation among the subfamilies and groups, it was found to have a high level of conservation among homologs. This suggests a possible diversity of solute transport by the AQPs and the involvement of the conserved Ar/R SFs in the process. In plants, only a few protein crystallographic studies are available and the current understanding of four Ar/R SF is mostly based on AQPs from animal and microbial origin^15^. Interestingly, a newly reported fifth AA, involved in the formation of SF in AtTIP2-1^41^, was observed to be conserved in TIPs and all other AQPs suggesting the possibility of five AA SF involved in solute permeability in plant AQPs.

### Tissue specificity of the AQPs

Very limited efforts have been deployed to understand tissue specificity of genome-wide AQPs in plants. Ubiquitous expression in roots and leaves for PIPs and TIPs, the two subfamilies with consistently higher expression, have been widely reported. However, other crucial stages for crop yield such as seed development have not been well studied. In this context, transcriptomic analysis during seed development provides new insights into AQPs role. In early developmental stages, upregulation of many PIPs and TIPs in seeds suggests an active growth and a higher level of water and nutrient movement in the tissues, whereas downregulation of most PIPs and TIPs (except for one TIP3) was observed as the seed matured, an observation consistent with the fact that water content of the seed reduces drastically during the maturation stage. In the same manner, the only TIP3 which was found continuously upregulated is thought to have a role in seed desiccation and in lipid metabolic pathways^16, 53^. Earlier, a similar type of expression pattern for TIPs and more particularly TIP3s was reported in soybean^16^. Given that both *B*. *napus* and soybean have high seed oil content, this suggests a key role of TIP3s in regulating oil content of oilseed crops. Negative correlation of TIP3s with other subfamilies is expected because of their tissue specificity and higher expression in seeds.

### Expression of AQPs under abiotic stress

Differential expression of AQPs has been studied under several abiotic stress conditions including salinity, drought, and extreme temperature in many plant species^54, 55^. Most of the previous studies were performed with a limited number of AQPs using quantitative PCR expression profiling. As such, expression profiling covering all the canola AQPs provides a better genome-wide understanding of expression dynamics under water limiting environments. In previous efforts, a very conflicting pattern of AQP expression was observed in different plant species (as reviewed in Forrest and Bhave, 2007). Under drought stress, both up and down regulation of AQPs in root and leaves have been reported with no clear delineation of specific roles linked to precise AQPs. In the present study, contrasting AQP expression with notable downregulation in roots and upregulation in leaves was highlighted. It is well established that the pattern of AQPs expression can vary drastically with the magnitude of the stress^56^. For example, in *Vitis* hybrids, a severe stress decreased the overall expression of AQPs initially, but, following continuous stress, the expression of AQPs significantly increased in leaves and remained low in roots^56^. In another report, transgenic *Arabidopsis* plants overexpressing PIP1;4 or PIP2;5 showed a rapid water loss and became susceptible to water stress^57^. A similar case of overexpression of PIP1s has been observed in tobacco^58^. The reduced expression of AQPs is believed to prevent loss of metabolic energy under severe stress situations and/or prevent water loss from the root to the hypertonic surrounding environment. The contrasting response of AQPs in leaves and roots may also be attributed to different roles of AQPs in different tissues^59^.

### Aquaporins expression as an indicator of biotic stress

Plant pathogen development depends on the host for nutrition and water. Therefore, water movement at sites of infection is crucial for disease progress (Nature last week). However, AQPs have rarely been considered as candidate players in biotic stress studies. Most of the transcriptomic studies employing dual RNA-seq focus on pathogenicity-related genes and their host counterpart resistance genes and disease response genes^60–62^. In a recent pioneer study by Tian, *et al*.^26^, the group showed that an Arabidopsis aquaporin (AtPIP1;4) was involved in the transport of H_2_O_2_ and subsequent induction of disease immunity pathways. This report highlights convincingly the potential role of AQPs during biotic stress conditions. In the present study, analysis of dual RNA-seq data available for *L*. *maculans* inoculated in resistant canola genotype DF78 and susceptible genotype Westar showed high expression of PIPs exclusively in the resistant genotype (see Fig. 7). In a previous study, Mysore, *et al*.^63^ showed that overexpression of disease resistance gene *Pto* in line with upregulation of AQP genes, was more prominent after pathogen infection. A similar type of response was observed here with resistant genotype DF78 where expression of AQPs was upregulated in the early stage of infection. Overall it would appear that under disease stress, the expression of AQPs is affected as shown by the lower expression of PIP2s in a susceptible cultivar. However, it is hard to pinpoint the causal gene(s) and associated mechanisms involved in disease resistance. However, the response of AQPs under biotic stress is easier to separate with the analyses performed here with another set of transcriptomic data involving a tripartite interaction host-pathogen and biocontrol agent. As with infection with *L*. *maculans*, the AQP expression was downregulated compared to controls in *B*. *napus* plants infected with *S*. *sclerotiorum* (Ssp), but was less affected when the plants were inoculated with the biocontrol agent *P*. *chlororaphis* (PA23). Accordingly, a reduced AQP expression seems to be an indicator of biotic stress in plants. Further studies are needed to elucidate the direct role of AQPs in biotic stress regulation.

### Dosage-dependency of aquaporin homologs

Understanding of dosage-dependency of gene expression is important to predict several attributes of molecular regulations and functionality. Tan, *et al*.^18^ suggested that dosage-dependent genes were involved in the basic biological processes, such as growth and development, whereas dosage independent ones were likely involved in stress responses. In the present study, by analyzing available RNA-seq data, only 30% of AQPs showed dosage-dependency, suggesting a large proportion of genes involved in stress tolerance mechanisms. Furthermore, Tan, *et al*.^18^ reported that dosage-independent genes were more likely to take part in protein–protein interactions. This would mean that dosage-independent NIPs and most of the TIPs are probably involved in protein-protein interactions. In addition, information provided about dosage-dependency is also helpful to predict involvement of trans or cis regulation of gene expression^18^.

## Conclusions

Genome-wide identification and analysis of AQPs performed here in seven Brassicaceae species highlighted several novel findings explaining their evolution and functional regulation. By taking advantage of the first fully sequenced polyploid genome of *B*. *napus*, we have shown that recent genome-wide duplications resulted into a higher number of AQPs with practically no loss after duplication. This translated into showing that *B*. *napus* has the highest number of AQPs in a plant species to date. Expression analysis showed similar patterns of tissue-specific expression for *B*. *napus* AQPs as previously observed in soybean, rice and *Arabidopsis*. Analysis of AQPs expression in developing seeds suggested the key role of TIP3s in seed maturation. The study of TIP3s is particularly important in oilseed crops since maturation and desiccation greatly influences seed oil content. Furthermore, extensive analysis of AQPs under abiotic and biotic stress highlighted the involvement of AQPs in stress tolerance mechanisms. More particularly, analysis of infected plants treated with a biocontrol agent showed a lowered expression of AQPs, a phenomenon that opens leads to understand plant physiology under disease conditions. RNA-seq transcriptome data also helped to identify dosage-dependent and independent AQPs in canola that has relevance in understanding protein-protein interactions, cis or trans regulation, stress mechanisms and also in the planning of transgenic experiments where effects of overexpression can be predicted. The identification, classification, evolution and functional regulation of AQPs performed in the present study will be helpful for enhancing our understanding of AQPs and for the development of more sustainable stress tolerant crops.

## Electronic supplementary material

Supplementary information 1

Dataset 1

Dataset 2

Dataset 3

Dataset 4

Dataset 5
