# Supplementary material for: Analysis of aquaporins in Brassicaceae species reveals high-level of conservation and dynamic role against biotic and abiotic stress in canola
Source: Sci Rep. 2017 Jun 5;7:2771. doi: 10.1038/s41598-017-02877-9 (PMC5459863; doi:10.1038/s41598-017-02877-9)
Supplement: Supplementary file 1 — Supplementary information 1 [file 41598_2017_2877_MOESM1_ESM.doc]

**Analysis of aquaporins in Brassicaceae species reveals high-level of conservation and dynamic role against biotic and abiotic stress in canola**

## Humira Sonah, Rupesh K. Deshmukh, Caroline Labbé, Richard R. Bélanger

**Supplementary Table 1.** Details of RNA-Seq transcriptome data used for expression analysis of aquaporins in *Brassica napus*

| **S.No** | **Tissue** | **Treatment** | **Number of replications** | **BioProject** | **GEO accession** | **Title** |
| --- | --- | --- | --- | --- | --- | --- |
| 1 | *Sclerotinia sclerotiorum* infected leaf along with biocontrol agent *Pseudomonas chlororaphis* | | | | | |
|  | Leaf | Water | 3 | PRJNA331148 | GSE84798 | The biocontrol agent *Pseudomonas chlororaphis* PA23 primes *Brassica napus* defenses through distinct gene regulatory networks |
|  | PA23, Leaf | Pseudomonas chlororaphis (PA23) | 3 | PRJNA331148 | GSE84798 |
|  | Ss, Leaf | Sclerotinia sclerotiorum (Ss) | 3 | PRJNA331148 | GSE84798 |
|  | PA23+Ss, Leaf | PA23+Ss | 3 | PRJNA331148 | GSE84798 |
| 2 | Disease reaction of Susceptible and Resistant (LepR1) *Brassica napus* to *L. maculans* | | | | | |
|  | Cotyledon | Westar_mock_0dpi | 3 | PRJNA311316 | GSE77723 | Comprehensive RNA profiling of susceptible and resistant (LepR1) *Brassica napus* to *L. maculans* |
|  | Cotyledon | Westar_mock-3dpi | 3 | PRJNA311316 | GSE77723 |
|  | Cotyledon+ *L.maculans* | Westar_3dpi | 3 | PRJNA311316 | GSE77723 |
|  | Cotyledon | Westar_mock-7dpi | 3 | PRJNA311316 | GSE77723 |
|  | Cotyledon+ *L.maculans* | Westar_7dpi | 3 | PRJNA311316 | GSE77723 |
|  | Cotyledon | Westar_mock-11dpi | 3 | PRJNA311316 | GSE77723 |
|  | Cotyledon+ *L.maculans* | Westar_11dpi | 3 | PRJNA311316 | GSE77723 |
|  | Cotyledon | DF78_mock_0dpi | 3 | PRJNA311316 | GSE77723 |
|  | Cotyledon | DF78_mock-3dpi | 3 | PRJNA311316 | GSE77723 |
|  | Cotyledon+ *L.maculans* | DF78_3dpi | 3 | PRJNA311316 | GSE77723 |
|  | Cotyledon | DF78_mock-7dpi | 3 | PRJNA311316 | GSE77723 |
|  | Cotyledon+ *L.maculans* | DF78_7dpi | 3 | PRJNA311316 | GSE77723 |
|  | Cotyledon | DF78_mock-11dpi | 3 | PRJNA311316 | GSE77723 |
|  | Cotyledon+ *L.maculans* | DF78_11dpi | 3 | PRJNA311316 | GSE77723 |
| 3 | Drought stress Transcriptome or Gene expression | | | | | |
|  | Leaf | Control | 1 | PRJNA256233 | NA | *Brassica napus* seedling root and leaf transcriptome analysis exhibiting drought tolerance |
|  | Root | Control | 1 | PRJNA256233 | NA |
|  | Leaf | Drought | 1 | PRJNA256233 | NA |
|  | Root | Drought | 1 | PRJNA256233 | NA |
| 4 | Gene/genome dosage balance | | | | | |
|  | Leaf | AA | 2 (Six samples) | PRJNA322687 | GSE81845 | Genome-wide gene/genome dosage imbalance regulates gene expressions in synthetic *Brassica napus* and derivatives (AC, AAC, CCA, CCAA) |
|  | Leaf | CC | 2 (Six samples) | PRJNA322687 | GSE81845 |
|  | Leaf | AC | 2 (Six samples) | PRJNA322687 | GSE81845 |
|  | Leaf | AAC | 2 (Six samples) | PRJNA322687 | GSE81845 |
|  | Leaf | CCA | 2 (Six samples) | PRJNA322687 | GSE81845 |
|  | Leaf | CCAA | 2 (Six samples) | PRJNA322687 | GSE81845 |
| 5 | *Brassica napus* seed at different developmental stages | | | | | |
|  | Seed | 2 Weeks after pollination(WAP) | 1 | PRJNA311067 | GSE77637 | RNA sequencing of *Brassica napus* seeds at different developmental stages (rape) |
|  | Seed | 4 WAP | 1 | PRJNA311067 | GSE77637 |
|  | Seed | 6 WAP | 1 | PRJNA311067 | GSE77637 |
|  | Seed | 8 WAP | 1 | PRJNA311067 | GSE77637 |

**Detailed materials and methods of corresponding projects provided in Supplementary Table 1. The materials and methods were taken from the corresponding projects**

**Project: PRJNA331148 (https://www.ncbi.nlm.nih.gov/geo/query/acc.cgi?acc=GSM2251576)**

One day prior to fungal pathogen exposure, *B. napus* plants at the 30% flowering stage were sprayed until dripping with a 2x10^8^ cfu/mL solution of PA23 resuspended in sterile water supplemented with 0.02% Tween 20 as a surfactant. Plants not receiving biocontrol treatment (water control and Ss only groups) were sprayed with sterile water (0.02% Tween 20). Plants were sealed in clear bags to preserve relative humidity and returned to the growth chamber for 24 hours. The following day, bags were removed and plants receiving the pathogen treatment were sprayed with an 8x10^4^ spores/mL solution of S*. sclerotiorum* ascospores resuspended in sterile water (0.02% Tween 20). Control plants and plants to be exposed only to PA23 were sprayed with sterile water (0.02% Tween 20). Plants were transferred to a humidity chamber with humidity levels of 70-90% for 72 hours. During this time, plants were gently shaken at the base twice to encourage petals to detach and fall into the plant canopy. Three leaves per plant and three plants per treatment group were used for each biological replicate. Leaves upon which petals had landed were used for collection, as these are sites of potential infection. The petal was removed from the leaf and approximately 1cm2 area of leaf tissue surrounding the site was collected with a scalpel. For S. sclerotiorum-infected leaves, green tissue immediately surrounding the lesion was collected. Cuttings were flash frozen in liquid nitrogen, and stored at -80℃ for no more than 2 days before processing.

Growth protocol *Brassica napus* cv. Westar plants were grown in Sunshine Mix #1 soil in growth chambers at 21ºC with a light/dark photoperiod of 16h/8h and 0% humidity

Extraction protocol: Total RNA was extracted using PureLink® Plant RNA Reagent (Invitrogen). DNA contamination was removed using Turbo DNA-freeTM kit (Ambion), following the manufacturer’s instructions. RNA concentration was verified using a NanoVue spectrophotometer (GE Healthcare), and quality was measured using an Agilent 2100 Bioanalyzer with Agilent RNA 6000 Pico and Nano Chips (Agilent Technologies; Santa Clara, CA, USA).

RNA-seq libraries were prepared according to the alternative HTR protocol (C2) described by Kumar with the exception of PCR enrichment of the libraries, where the number of cycles was adjusted to 11. Libraries were validated using the Agilent Bioanalyzer High Sensitivity DNA Assay with DNA chips (Agilent Technologies). The desired fragment sizes of sheared cDNA with ligated adapters were isolated using the E-Gel® electrophoresis system (Invitrogen). 100 bp single-end RNA sequencing was carried out at Génome Québec (Montreal, Canada) on the Illumina HiSeq 2000 platform.

Sequenced reads were analyzed to remove barcode adapters and low quality reads using the Trimmomatic tool. The parameters for Trimmomatic which maximized mapping efficiency to the B. napus genome [B. napus: v.4.1, Chalhoub et al. was determined using FastQC reports for quality control (http://www.bioinformatics.babraham.ac.uk/projects/fastqc/) followed by alignment using Tophat2 v.2.1.0. Alignment was completed using Tophat2 v.2.1.0. Alignment of reads to these genomes was performed in high-sensitivity mode using B. napus reference annotation v5 from Chalhoub et al. as guides. The cufflinks and cuffmerge tools within the Cufflinks v.2.2.1 suite were used construct a transcriptome from the reads and identify novel transcripts. Transdecoder was used to identify open reading frames (ORFs) within transcript sequences. Genes were identified by aligning translated ORF sequences with proteins in the Arabidopsis TAIR10, NCBI and Uniprot databases using BLAST.

Read counts were normalized to FPKM values using the cuffquant and cuffdiff tools in the Cufflinks package with default settings. Genome_build: B. napus: v.4.1, Chalhoub et al.

**Project: PRJNA311316- (https://www.ncbi.nlm.nih.gov/geo/query/acc.cgi?acc=****GSM2057885)**

RNA was collected from three biological replicates of infected and mock inoculated B. napus cotyledons at 0, 3, 7, and 11 dpi. Total RNA was isolated by using PureLink® Plant RNA Reagent (Ambion) and treated with TURBO DNA-free™ Kit (Ambion) according to the manufacturer’s instructions. RNA quality and integrity was measured using the 2100 Bioanalyzer (Agilent Technologies) with the Agilent 2100 PicoChip. RNA-sequencing libraries were prepared according to alternative HTR protocol (C2) established by R. Kumar et al. (2012) with the exception of library PCR enrichment that was carried out using 11 PCR cycles. RNA sequencing libraries were validated using the Agilent Bioanalyzer DNA chips (Agilent Technologies) and quantified using the Quant-iT dsDNA Assay kit (ThermoFisher Scientific).

Barcode adaptors from the RNA sequence reads were clipped and low quality reads were removed (read quality < 30) using the Trimmomatic software (Bolger et al., 2014). RNA sequence reads passing quality filter were aligned to the B. napus genome (v4.1, Chalhoub et al. 2014) using Tophat2 of the Trapnell cufflinks package (Trapnell et al., 2012). Tophat2 alignment was performed in high-sensitivity mode and used B. napus reference annotation v5.0 as a guide (Chalhoub et al., 2014). Identification of novel transcripts was performed using cufflinks v2.2.1 (Trapnell et al., 2012) and transcript sequences were extracted from this annotation using BedTools. Cuffquant, CuffNorm and Cuffdiff were used to generate normalized counts in FPKM and to identify differentially expressed genes (pooled dispersion method). Genes were considered significantly differentially expressed with a corrected p-value of < 0.05 (false discovery rate = 0.05). Genome_build: B. napus v. 4.1

**Project: PRJNA322687 (https://www.ncbi.nlm.nih.gov/geo/query/acc.cgi?acc=GSM2176571)**

Using TRIzol reagent (Invitrogen, Life Technologies) according the manufacturer’s protocol, total RNA was extracted from two biological replicates (three plants per replicate) for each genotype.

NanoDrop ND 1000 (NanoDrop technologies) was used to initially calculate the quality and quantity of the extracted RNA, and then the RNA Integrity Number (RIN) value was assessed by Agilent Technologies 2100 Bioanalyzer (Agilent). Only when the value of RIN was higher than 8, the RNA was used to prepare the c-DNA library according to the TruSeq RNA Sample Prep v2 protocol.

100bp paired-end reads were generated via Illumina HiSeqTM 2000. The clean reads were directly aligned to the reference genome of Brassica napus using HISAT (HISAT version 0.1.6-beta) with the default parameters. The genes were assembled with Cufflinks according to a reference-guided method. The differentially expressed genes between three different samples were computed on RNA-seq data by using CuffDiff2. Genome_build: Brassica napus.annotation_v5.gff3

**Project: PRJNA311067 (Details from https://www.ncbi.nlm.nih.gov/geo/query/acc.cgi?acc=GSM2055543)**

B. napus seeds were harvested at 2, 4, 6 and 8 WAP. After being dissected from the siliques, these seeds were frozen in liquid nitrogen immediately and then stored at -80°C. The total RNA from the four samples were extracted and purified using the RNAprep pure Plant Kit (DP 432, TIANGEN BIOTECH (BEIJING) CO., LTD) according to the manufacturer’s protocol. RNA purity was checked with NanoPhotometer® spectrophotometer (IMPLEN, CA, USA). The concentration was measured with Qubit® RNA Assay Kit and Qubit® 2.0 Fluorometer (Life Technologies, CA, USA). The integrity was assessed using the Agilent 2100 Bioanalyzer and RNA Nano 6000 kit (Agilent Technologies, CA, USA). cDNA libraries for samples from the four time points of seed development were constructed using NEBNext® Ultra™ RNA Library Prep Kit for Illumina® (NEB, USA) according to the manufacturer’s protocol. Library quality was assessed using the Agilent 2100 Bioanalyzer. The library preparations were sequenced and 100-bp paired-end reads were generated by an Illumina Hiseq 2000 platform. The clean reads were screened from raw sequencing reads by removing reads containing adapter, ploy-N and low quality reads. The clean reads were aligned to the B. napus reference genome using the TopHat program. The expression levels of all genes from the B. napus reference annotation were quantified using htseq-count 0.6.1p2. RNA-Seq raw counts were normalized by the DESeq normalization Time-series differential expression analysis was carried out using the maSigPro package. The p-values were corrected for multiple comparisons by the Benjamini & Hochberg false discovery rate procedure. We set Q = 0.05 and rsq = 0.7 to get significant genes, and we set k = 8 to classify significant genes. Genome_build: Brassica_napus_v4.1.chromosomes.fa

**Supplementary Table 2**. Details of aquaporins categorized into subfamilies and groups across eight Brassicaceae species

| **Subfamily** | **Group** | ***Arabidopsis thaliana*** | ***Arabidopsis Lyrata*** | ***Capsella rubella*** | ***Capsella grandiflora*** | ***Brassica***  ***rapa*** | ***Brassica napus*** | ***Brassica oleracea*** | ***Eutrema salsugineum*** |
| --- | --- | --- | --- | --- | --- | --- | --- | --- | --- |
| PIP | PIP1 | 5 | 5 | 5 | 5 | 8 | 19 | 9 | 6 |
|  | PIP2 | 8 | 8 | 9 | 8 | 14 | 24 | 15 | 8 |
| TIP | TIP1 | 3 | 3 | 3 | 2 | 4 | 9 | 5 | 3 |
|  | TIP2 | 3 | 5 | 4 | 3 | 6 | 13 | 6 | 4 |
|  | TIP3 | 2 | 2 | 2 | 2 | 4 | 10 | 2 | 2 |
|  | TIP4 | 1 | 1 | 1 | 1 | 1 | 1 | 1 | 1 |
|  | TIP5 | 1 | 1 | 1 | 1 | 1 | 2 | 1 | 1 |
| NIP | NIP1 | 2 | 2 | 2 | 2 | 2 | 4 | 2 | 1 |
|  | NIP2 | 1 | 1 | 1 | 1 | 2 | 4 | 2 | 1 |
|  | NIP3 | 1 | 1 | 1 | 1 | 2 | 6 | 3 | 1 |
|  | NIP4 | 2 | 2 | 2 | 2 | 2 | 6 | 3 | 3 |
|  | NIP5 | 1 | 1 | 1 | 1 | 2 | 5 | 2 | 1 |
|  | NIP6 | 1 | 2 | 1 | 1 | 2 | 4 | 1 | 1 |
|  | NIP7 | 1 | 1 | 1 | 1 | 1 | 2 | 1 | 1 |
| SIP | SIP1 | 2 | 2 | 2 | 2 | 3 | 6 | 3 | 2 |
|  | SIP2 | 1 | 1 | 1 | 1 | 3 | 5 | 1 | 1 |
|  | Total | 35 | 38 | 37 | 34 | 57 | 120 | 57 | 37 |

**Supplementary Table 3.** Details of aquaporins identified in *Brassica napus* genome through BLAST search performed using the query sequences of known aquaporins. E-value 10 E-5 and bit-score >100 was used as cutoff to claim significant match.

| **Sr. No.** | **Query Sequence** | **Gene_ID** | **Locus_ID** | **Percent Similarity** | **E-Value** | **Bit-Score** |
| --- | --- | --- | --- | --- | --- | --- |
| 1 | AtNIP1-2 | BnaNIP1-2 | BnaA01g09720D | 89.56 | 3.00E-149 | 525 |
| 2 | AtTIP2-1 | BnaTIP2-1 | BnaA01g28120D | 96.76 | 1.00E-133 | 473 |
| 3 | AtSIP1-1 | BnaSIP1-1 | BnaA01g33700D | 85.78 | 7.00E-108 | 387 |
| 4 | AtTIP2-2 | BnaTIP2-2 | BnaA01g35340D | 96.8 | 4.00E-135 | 478 |
| 5 | AtPIP2-4 | BnaPIP2-4 | BnaA02g06180D | 81.69 | 4.00E-129 | 458 |
| 6 | AtTIP3-1 | BnaTIP3-1 | BnaA02g16380D | 92.91 | 4.00E-143 | 504 |
| 7 | AtNIP6-1 | BnaNIP6-1 | BnaA02g19440D | 95.41 | 3.00E-166 | 582 |
| 8 | AtNIP5-1 | BnaNIP5-1 | BnaA02g22030D | 93.75 | 8.00E-161 | 563 |
| 9 | AtTIP2-3 | BnaTIP2-3 | BnaA02g25440D | 78.4 | 3.00E-103 | 372 |
| 10 | AtTIP1-2 | BnaTIP1-2 | BnaA02g28130D | 91.3 | 2.00E-133 | 472 |
| 11 | AtNIP6-1 | BnaNIP6-1 | BnaA02g36290D | 94.43 | 2.00E-164 | 576 |
| 12 | AtPIP2-4 | BnaPIP2-4 | BnaA03g08820D | 83.39 | 4.00E-134 | 475 |
| 13 | AtPIP2-2 | BnaPIP2-2 | BnaA03g17020D | 95.05 | 6.00E-157 | 550 |
| 14 | AtPIP2-2 | BnaPIP2-2 | BnaA03g17030D | 93.33 | 1.00E-156 | 550 |
| 15 | AtPIP2-6 | BnaPIP2-6 | BnaA03g18300D | 94.74 | 2.00E-156 | 549 |
| 16 | AtPIP1-2 | BnaPIP1-2 | BnaA03g21210D | 98.95 | 1.00E-164 | 576 |
| 17 | AtNIP5-1 | BnaNIP5-1 | BnaA03g24370D | 92.43 | 2.00E-160 | 562 |
| 18 | AtPIP1-4 | BnaPIP1-4 | BnaA03g27130D | 98.26 | 2.00E-162 | 568 |
| 19 | AtTIP2-1 | BnaTIP2-1 | BnaA03g34110D | 96.36 | 3.00E-133 | 471 |
| 20 | AtNIP1-2 | BnaNIP1-2 | BnaA03g43810D | 89.6 | 3.00E-149 | 525 |
| 21 | AtPIP1-5 | BnaPIP1-5 | BnaA03g45950D | 97.56 | 1.00E-163 | 573 |
| 22 | AtPIP1-1 | BnaPIP1-1 | BnaA04g00710D | 98.62 | 1.00E-122 | 437 |
| 23 | AtSIP2-1 | BnaSIP2-1 | BnaA04g02840D | 87.76 | 9.00E-49 | 191 |
| 24 | AtNIP4-1 | BnaNIP4-1 | BnaA04g08310D | 82.33 | 9.00E-138 | 487 |
| 25 | AtTIP4-1 | BnaTIP4-1 | BnaA04g15110D | 90.91 | 5.00E-67 | 251 |
| 26 | AtPIP1-2 | BnaPIP1-2 | BnaA04g26560D | 98.95 | 6.00E-165 | 577 |
| 27 | AtNIP4-1 | BnaNIP4-1 | BnaA04g27980D | 89.38 | 3.00E-117 | 419 |
| 28 | AtPIP1-2 | BnaPIP1-2 | BnaA05g05230D | 99.3 | 3.00E-165 | 578 |
| 29 | AtPIP2-2 | BnaPIP2-2 | BnaA05g07290D | 94.36 | 5.00E-104 | 375 |
| 30 | AtPIP2-2 | BnaPIP2-2 | BnaA05g07300D | 87.1 | 7.00E-154 | 540 |
| 31 | AtNIP2-1 | BnaNIP2-1 | BnaA05g09450D | 85.76 | 2.00E-138 | 489 |
| 32 | AtNIP2-1 | BnaNIP2-1 | BnaA05g09470D | 86.11 | 4.00E-140 | 494 |
| 33 | AtNIP3-1 | BnaNIP3-1 | BnaA05g16540D | 74.91 | 3.00E-121 | 432 |
| 34 | AtNIP3-1 | BnaNIP3-1 | BnaA05g16550D | 72.45 | 2.00E-128 | 456 |
| 35 | AtNIP3-1 | BnaNIP3-1 | BnaA05g16580D | 78.02 | 5.00E-36 | 149 |
| 36 | AtTIP2-1 | BnaTIP2-1 | BnaA05g23460D | 95.95 | 2.00E-132 | 469 |
| 37 | AtNIP7-1 | BnaNIP7-1 | BnaA05g31180D | 86.91 | 5.00E-130 | 461 |
| 38 | AtSIP1-1 | BnaSIP1-1 | BnaA05g37440D | 89.12 | 4.00E-120 | 428 |
| 39 | AtTIP3-2 | BnaTIP3-2 | BnaA06g12030D | 91.76 | 2.00E-142 | 502 |
| 40 | AtTIP5-1 | BnaTIP5-1 | BnaA06g17390D | 91.86 | 2.00E-132 | 469 |
| 41 | AtTIP1-2 | BnaTIP1-2 | BnaA06g32840D | 90.91 | 5.00E-132 | 468 |
| 42 | AtTIP2-3 | BnaTIP2-3 | BnaA06g40020D | 91.24 | 2.00E-128 | 455 |
| 43 | AtPIP2-1 | BnaPIP2-1 | BnaA07g02320D | 95.38 | 4.00E-30 | 129 |
| 44 | AtNIP5-1 | BnaNIP5-1 | BnaA07g16310D | 89.47 | 2.00E-155 | 545 |
| 45 | AtPIP2-5 | BnaPIP2-5 | BnaA07g16510D | 93.71 | 6.00E-158 | 554 |
| 46 | AtSIP2-1 | BnaSIP2-1 | BnaA07g17050D | 90.34 | 6.00E-124 | 441 |
| 47 | AtTIP3-1 | BnaTIP3-1 | BnaA07g22790D | 92.54 | 1.00E-142 | 503 |
| 48 | AtTIP3-1 | BnaTIP3-1 | BnaA07g30640D | 89.93 | 2.00E-138 | 489 |
| 49 | AtNIP6-1 | BnaNIP6-1 | BnaA07g35330D | 96.07 | 2.00E-168 | 588 |
| 50 | AtNIP3-1 | BnaNIP3-1 | BnaA08g07040D | 88.13 | 4.00E-141 | 498 |
| 51 | AtPIP2-7 | BnaPIP2-7 | BnaA08g10860D | 95.37 | 1.00E-154 | 543 |
| 52 | AtPIP2-1 | BnaPIP2-1 | BnaA09g33720D | 94.08 | 4.00E-157 | 551 |
| 53 | AtPIP2-5 | BnaPIP2-5 | BnaA09g34600D | 94.76 | 5.00E-159 | 557 |
| 54 | AtSIP2-1 | BnaSIP2-1 | BnaA09g36250D | 80.17 | 4.00E-107 | 385 |
| 55 | AtPIP1-1 | BnaPIP1-1 | BnaA09g39170D | 97.55 | 3.00E-163 | 572 |
| 56 | AtTIP3-2 | BnaTIP3-2 | BnaA09g44820D | 92.88 | 1.00E-144 | 509 |
| 57 | AtTIP1-3 | BnaTIP1-3 | BnaA09g51590D | 96.83 | 1.00E-137 | 486 |
| 58 | AtPIP1-4 | BnaPIP1-4 | BnaA09g51960D | 97.91 | 5.00E-162 | 567 |
| 59 | AtPIP1-3 | BnaPIP1-3 | BnaA10g00360D | 96.85 | 7.00E-162 | 567 |
| 60 | AtPIP2-4 | BnaPIP2-4 | BnaA10g13480D | 82.33 | 1.00E-132 | 469 |
| 61 | AtSIP1-2 | BnaSIP1-2 | BnaA10g16540D | 84.36 | 2.00E-116 | 416 |
| 62 | AtPIP2-7 | BnaPIP2-7 | BnaAnng11630D | 96.44 | 6.00E-157 | 550 |
| 63 | AtTIP1-1 | BnaTIP1-1 | BnaAnng22640D | 93.67 | 4.00E-119 | 424 |
| 64 | AtPIP1-1 | BnaPIP1-1 | BnaAnng23190D | 97.57 | 3.00E-115 | 412 |
| 65 | AtTIP1-1 | BnaTIP1-1 | BnaAnng24130D | 93.67 | 4.00E-119 | 424 |
| 66 | AtPIP2-7 | BnaPIP2-7 | BnaC01g03410D | 96.8 | 2.00E-157 | 552 |
| 67 | AtNIP1-2 | BnaNIP1-2 | BnaC01g11410D | 88.89 | 7.00E-149 | 524 |
| 68 | AtNIP5-1 | BnaNIP5-1 | BnaC01g23040D | 88.89 | 6.00E-31 | 132 |
| 69 | AtSIP1-1 | BnaSIP1-1 | BnaC01g40230D | 86.67 | 1.00E-108 | 390 |
| 70 | AtTIP2-2 | BnaTIP2-2 | BnaC01g41690D | 96.4 | 8.00E-134 | 473 |
| 71 | AtTIP2-1 | BnaTIP2-1 | BnaC01g44580D | 97.17 | 2.00E-134 | 475 |
| 72 | AtTIP1-3 | BnaTIP1-3 | BnaC02g27930D | 56.25 | 1.00E-30 | 131 |
| 73 | AtNIP5-1 | BnaNIP5-1 | BnaC02g29210D | 92.76 | 3.00E-159 | 558 |
| 74 | AtTIP1-2 | BnaTIP1-2 | BnaC02g36210D | 91.3 | 2.00E-133 | 472 |
| 75 | AtTIP2-3 | BnaTIP2-3 | BnaC02g46870D | 92.8 | 7.00E-130 | 460 |
| 76 | AtPIP2-4 | BnaPIP2-4 | BnaC03g11160D | 83.8 | 1.00E-135 | 479 |
| 77 | AtPIP2-1 | BnaPIP2-1 | BnaC03g20550D | 98.61 | 1.00E-36 | 150 |
| 78 | AtPIP2-6 | BnaPIP2-6 | BnaC03g21800D | 94.39 | 2.00E-156 | 548 |
| 79 | AtPIP1-2 | BnaPIP1-2 | BnaC03g25510D | 98.25 | 4.00E-164 | 574 |
| 80 | AtNIP5-1 | BnaNIP5-1 | BnaC03g28980D | 92.76 | 4.00E-161 | 565 |
| 81 | AtPIP1-4 | BnaPIP1-4 | BnaC03g32130D | 97.91 | 3.00E-162 | 568 |
| 82 | AtTIP2-1 | BnaTIP2-1 | BnaC03g39560D | 95.95 | 7.00E-133 | 470 |
| 83 | AtSIP2-1 | BnaSIP2-1 | BnaC03g54990D | 86.92 | 4.00E-122 | 434 |
| 84 | AtPIP2-7 | BnaPIP2-7 | BnaC03g65520D | 95.37 | 4.00E-155 | 544 |
| 85 | AtPIP2-2 | BnaPIP2-2 | BnaC03g72010D | 98.11 | 2.00E-87 | 320 |
| 86 | AtPIP1-2 | BnaPIP1-2 | BnaC04g04640D | 99.65 | 2.00E-166 | 582 |
| 87 | AtPIP2-2 | BnaPIP2-2 | BnaC04g08090D | 95.09 | 8.00E-158 | 553 |
| 88 | AtPIP2-2 | BnaPIP2-2 | BnaC04g08100D | 94.74 | 4.00E-158 | 554 |
| 89 | AtNIP2-1 | BnaNIP2-1 | BnaC04g10820D | 86.81 | 2.00E-140 | 495 |
| 90 | AtNIP2-1 | BnaNIP2-1 | BnaC04g10830D | 86.46 | 7.00E-140 | 494 |
| 91 | AtSIP2-1 | BnaSIP2-1 | BnaC04g24760D | 86.92 | 4.00E-122 | 434 |
| 92 | AtNIP4-1 | BnaNIP4-1 | BnaC04g30520D | 82.33 | 5.00E-138 | 488 |
| 93 | AtNIP4-1 | BnaNIP4-1 | BnaC04g34450D | 84.81 | 2.00E-138 | 489 |
| 94 | AtNIP4-1 | BnaNIP4-1 | BnaC04g34460D | 88.46 | 2.00E-74 | 276 |
| 95 | AtTIP4-1 | BnaTIP4-1 | BnaC04g38040D | 92.77 | 5.00E-133 | 471 |
| 96 | AtPIP1-2 | BnaPIP1-2 | BnaC04g50590D | 98.95 | 6.00E-165 | 577 |
| 97 | AtPIP1-3 | BnaPIP1-3 | BnaC05g00440D | 97.2 | 1.00E-162 | 569 |
| 98 | AtTIP3-2 | BnaTIP3-2 | BnaC05g13770D | 92.51 | 1.00E-143 | 506 |
| 99 | AtNIP3-1 | BnaNIP3-1 | BnaC05g29140D | 68.73 | 2.00E-103 | 373 |
| 100 | AtNIP3-1 | BnaNIP3-1 | BnaC05g29150D | 72 | 3.00E-128 | 456 |
| 101 | AtTIP2-1 | BnaTIP2-1 | BnaC05g37160D | 96.76 | 7.00E-133 | 470 |
| 102 | AtNIP7-1 | BnaNIP7-1 | BnaC05g45720D | 87.64 | 7.00E-132 | 467 |
| 103 | AtSIP1-1 | BnaSIP1-1 | BnaC05g47470D | 89.12 | 5.00E-120 | 427 |
| 104 | AtTIP2-1 | BnaTIP2-1 | BnaC06g05270D | 80.31 | 2.00E-113 | 406 |
| 105 | AtPIP2-1 | BnaPIP2-1 | BnaC06g14590D | 91.99 | 7.00E-153 | 537 |
| 106 | AtPIP2-5 | BnaPIP2-5 | BnaC06g15450D | 93.36 | 5.00E-157 | 551 |
| 107 | AtTIP3-1 | BnaTIP3-1 | BnaC06g23750D | 92.54 | 1.00E-142 | 503 |
| 108 | AtTIP3-1 | BnaTIP3-1 | BnaC06g34100D | 89.93 | 6.00E-138 | 487 |
| 109 | AtNIP6-1 | BnaNIP6-1 | BnaC06g40240D | 96.07 | 5.00E-168 | 587 |
| 110 | AtNIP4-2 | BnaNIP4-2 | BnaC06g42210D | 86.52 | 2.00E-135 | 479 |
| 111 | AtNIP5-1 | BnaNIP5-1 | BnaC06g42490D | 90.28 | 3.00E-31 | 133 |
| 112 | AtNIP5-1 | BnaNIP5-1 | BnaC06g42500D | 67.68 | 2.00E-32 | 137 |
| 113 | AtTIP2-3 | BnaTIP2-3 | BnaC07g20220D | 91.63 | 8.00E-129 | 457 |
| 114 | AtTIP1-2 | BnaTIP1-2 | BnaC07g23630D | 90.51 | 3.00E-131 | 465 |
| 115 | AtNIP1-2 | BnaNIP1-2 | BnaC07g35550D | 88.93 | 4.00E-148 | 521 |
| 116 | AtPIP1-5 | BnaPIP1-5 | BnaC07g38190D | 96.17 | 5.00E-162 | 567 |
| 117 | AtPIP2-7 | BnaPIP2-7 | BnaC07g45370D | 96.2 | 9.00E-132 | 467 |
| 118 | AtNIP3-1 | BnaNIP3-1 | BnaC08g07910D | 82.88 | 1.00E-153 | 540 |
| 119 | AtPIP2-5 | BnaPIP2-5 | BnaC08g25570D | 94.41 | 2.00E-158 | 556 |
| 120 | AtSIP2-1 | BnaSIP2-1 | BnaC08g27810D | 87.95 | 1.00E-38 | 157 |
| 121 | AtSIP2-1 | BnaSIP2-1 | BnaC08g27820D | 75.96 | 1.00E-38 | 157 |
| 122 | AtPIP1-1 | BnaPIP1-1 | BnaC08g31360D | 97.9 | 1.00E-163 | 573 |
| 123 | AtTIP3-2 | BnaTIP3-2 | BnaC08g37510D | 92.88 | 1.00E-144 | 509 |
| 124 | AtPIP2-4 | BnaPIP2-4 | BnaC09g53920D | 81.98 | 3.00E-129 | 459 |
| 125 | AtSIP1-2 | BnaSIP1-2 | BnaC09g54320D | 84.36 | 6.00E-116 | 414 |
| 126 | AtTIP1-3 | BnaTIP1-3 | BnaCnng01570D | 96.83 | 1.00E-137 | 486 |
| 127 | AtPIP1-4 | BnaPIP1-4 | BnaCnng02360D | 97.21 | 4.00E-161 | 564 |
| 128 | AtPIP1-3 | BnaPIP1-3 | BnaCnng08780D | 95.45 | 2.00E-158 | 556 |
| 129 | AtTIP5-1 | BnaTIP5-1 | BnaCnng15220D | 91.86 | 3.00E-132 | 468 |
| 130 | AtSIP2-1 | BnaSIP2-1 | BnaCnng20470D | 90.76 | 6.00E-124 | 441 |
| 131 | AtTIP1-1 | BnaTIP1-1 | BnaCnng24720D | 93.23 | 7.00E-135 | 477 |
| 132 | AtPIP2-1 | BnaPIP2-1 | BnaCnng31040D | 93.73 | 1.00E-156 | 549 |
| 133 | AtTIP3-1 | BnaTIP3-1 | BnaCnng50290D | 93.28 | 3.00E-143 | 505 |
| 134 | AtNIP4-1 | BnaNIP4-1 | BnaCnng65250D | 62.85 | 2.00E-93 | 339 |
| 135 | AtNIP5-1 | BnaNIP5-1 | BnaCnng75510D | 94.12 | 3.00E-39 | 159 |

**Supplementary Table 4**. Details of genomic distribution of Aquaporins in seven Brassicaceae species

| **Gene ID** | **Locus name** | **Scaffold** | **start** | **end** |
| --- | --- | --- | --- | --- |
| **Arabidopsis lyrata** |  |  |  |  |
| AlPIP1-3 | 470115 | scaffold_1 | 246920 | 248384 |
| AlNIP3-1 | 912995 | scaffold_1 | 14483615 | 14486143 |
| AlTIP2-1a | 914379 | scaffold_1 | 27736112 | 27737613 |
| AlTIP3-2 | 920704 | scaffold_1 | 7484134 | 7485440 |
| AlTIP2-3b | 475524 | scaffold_2 | 8207002 | 8213005 |
| AlTIP3-1 | 476450 | scaffold_2 | 15727537 | 15728955 |
| AlNIP6-1b | 477170 | scaffold_2 | 18894029 | 18895629 |
| AlSIP1-1 | 477669 | scaffold_3 | 1358369 | 1360119 |
| AlNIP7-1 | 477911 | scaffold_3 | 2425798 | 2430202 |
| AlTIP2-1b | 479077 | scaffold_3 | 6825271 | 6826887 |
| AlPIP2-8 | 480563 | scaffold_3 | 21530610 | 21532362 |
| AlTIP5-1 | 905970 | scaffold_3 | 15002645 | 15003415 |
| AlTIP1-1 | 482663 | scaffold_4 | 17505307 | 17506436 |
| AlPIP2-2a | 482707 | scaffold_4 | 17775096 | 17776402 |
| AlPIP2-6 | 482920 | scaffold_4 | 18926016 | 18929461 |
| AlPIP1-2 | 483737 | scaffold_4 | 22352653 | 22354402 |
| AlNIP2-1 | 901267 | scaffold_4 | 16312543 | 16313690 |
| AlTIP4-1 | 932757 | scaffold_4 | 5027300 | 5028658 |
| AlPIP2-2b | 934366 | scaffold_4 | 17779488 | 17780657 |
| AlTIP1-2 | 484417 | scaffold_5 | 2922085 | 2923763 |
| AlPIP2-1 | 485715 | scaffold_5 | 16572971 | 16575549 |
| AlPIP2-5 | 485893 | scaffold_5 | 17185445 | 17187808 |
| AlPIP1-1 | 486618 | scaffold_5 | 20261131 | 20263194 |
| AlSIP2-1 | 938662 | scaffold_5 | 18170051 | 18171253 |
| AlSIP1-2 | 326244 | scaffold_6 | 7601458 | 7602756 |
| AlNIP5-1 | 942850 | scaffold_6 | 20256087 | 20259369 |
| AlTIP1-3 | 943598 | scaffold_6 | 24301625 | 24302424 |
| AlPIP1-4 | 943716 | scaffold_6 | 24843309 | 24844958 |
| AlNIP6-1a | 887390 | scaffold_627 | 4327 | 5429 |
| AlNIP4-1 | 330314 | scaffold_7 | 20814568 | 20818449 |
| AlPIP2-7 | 491143 | scaffold_7 | 2496925 | 2498535 |
| AlPIP1-5 | 492536 | scaffold_7 | 8110083 | 8111701 |
| AlNIP1-1 | 492953 | scaffold_7 | 10448156 | 10450205 |
| AlNIP1-2 | 492969 | scaffold_7 | 10519001 | 10521437 |
| AlTIP2-2 | 493143 | scaffold_7 | 11546623 | 11547772 |
| AlNIP4-2 | 891407 | scaffold_7 | 20820782 | 20822592 |
| AlTIP2-3a | 494233 | scaffold_8 | 196879 | 198332 |
| AlPIP2-4 | 950814 | scaffold_8 | 19605775 | 19607294 |
|  |  |  |  |  |
| **Brassica oleracea** |  |  |  |  |
| BolCTIP1-2b | Bo00285s340.1 | #N/A | #N/A | #N/A |
| BolCTIP3-2a | Bo00796s040.1 | #N/A | #N/A | #N/A |
| BolCPIP2-7a | Bo1g006730.1 | C1 | 2082658 | 2082777 |
| BolCNIP1-2a | Bo1g020050.1 | C1 | 7256594 | 7256761 |
| BolCTIP2-2a | Bo1g051740.1 | C1 | 14900070 | 14900444 |
| BolCSIP1-1b | Bo1g152210.1 | C1 | 42751859 | 42752152 |
| BolCPIP2-4c | Bo2g024760.1 | C2 | 7104346 | 7104646 |
| BolCNIP5-1a | Bo2g110350.1 | C2 | 33180860 | 33181087 |
| BolCTIP2-3b | Bo2g132360.1 | C2 | 41418831 | 41419205 |
| BolCPIP2-4b | Bo3g017270.1 | C3 | 5629567 | 5629873 |
| BolCTIP1-1b | Bo3g031310.1 | C3 | 11504370 | 11504753 |
| BolCPIP2-2d | Bo3g031540.1 | C3 | 11702006 | 11702119 |
| BolCPIP2-2c | Bo3g031550.1 | C3 | 11714582 | 11714882 |
| BolCPIP2-6a | Bo3g034080.1 | C3 | 12780207 | 12780335 |
| BolCPIP1-2c | Bo3g039050.1 | C3 | 15513989 | 15514316 |
| BolCNIP5-1b | Bo3g044690.1 | C3 | 18525836 | 18526031 |
| BolCPIP1-4a | Bo3g054790.1 | C3 | 21597639 | 21597966 |
| BolCTIP2-1c | Bo3g068310.1 | C3 | 26781233 | 26781437 |
| BolCPIP2-7c | Bo3g167200.1 | C3 | 59012310 | 59012429 |
| BolCPIP1-2a | Bo4g025110.1 | C4 | 4158592 | 4158919 |
| BolCPIP2-2b | Bo4g030810.1 | C4 | 6844543 | 6844662 |
| BolCPIP2-2a | Bo4g030820.1 | C4 | 6848652 | 6848952 |
| BolCNIP2-1b | Bo4g039860.1 | C4 | 9070719 | 9070859 |
| BolCNIP2-1a | Bo4g039880.1 | C4 | 9080383 | 9080523 |
| BolCSIP2-1a | Bo4g112640.1 | C4 | 28627056 | 28627343 |
| BolCNIP4-1a | Bo4g136860.1 | C4 | 35830890 | 35831027 |
| BolCNIP4-1e | Bo4g143940.1 | C4 | 39144587 | 39144718 |
| BolCNIP4-1b | Bo4g143950.1 | C4 | 39153274 | 39153372 |
| BolCTIP4-1a | Bo4g158790.1 | C4 | 43249253 | 43249379 |
| BolCTIP1-1a | Bo4g186420.1 | C4 | 49458675 | 49459058 |
| BolCPIP1-2b | Bo4g195940.1 | C4 | 53111030 | 53111357 |
| BolCPIP1-3a | Bo5g001010.1 | C5 | 20409 | 20504 |
| BolCNIP3-1b | Bo5g091760.1 | C5 | 29777595 | 29777836 |
| BolCNIP3-1c | Bo5g091770.1 | C5 | 29817777 | 29818116 |
| BolCTIP2-1a | Bo5g126310.1 | C5 | 38807886 | 38808254 |
| BolCNIP7-1a | Bo5g144770.1 | C5 | 44694668 | 44694805 |
| BolCSIP1-1a | Bo5g148400.1 | C5 | 45757942 | 45758229 |
| BolCTIP2-1d | Bo6g027670.1 | C6 | 6209970 | 6210356 |
| BolCNIP4-2a | Bo6g058700.1 | C6 | 15748413 | 15748544 |
| BolCPIP2-1b | Bo6g067330.1 | C6 | 18723529 | 18723648 |
| BolCPIP2-5b | Bo6g068300.1 | C6 | 19556311 | 19556614 |
| BolCTIP3-1b | Bo6g087980.1 | C6 | 27970770 | 27971159 |
| BolCNIP6-1a | Bo6g124270.1 | C6 | 39560409 | 39560651 |
| BolCTIP2-3a | Bo7g077600.1 | C7 | 29227986 | 29228363 |
| BolCTIP1-2a | Bo7g082700.1 | C7 | 32701080 | 32701454 |
| BolCNIP1-2b | Bo7g106370.1 | C7 | 41248566 | 41248736 |
| BolCPIP1-5a | Bo7g108740.1 | C7 | 42863423 | 42864049 |
| BolCPIP2-7b | Bo7g117200.1 | C7 | 46856650 | 46856938 |
| BolCNIP3-1a | Bo8g030310.1 | C8 | 9370009 | 9370342 |
| BolCPIP2-1a | Bo8g082800.1 | C8 | 28640543 | 28640662 |
| BolCPIP2-5a | Bo8g086930.1 | C8 | 29338055 | 29338358 |
| BolCPIP1-1a | Bo8g095940.1 | C8 | 32880723 | 32881050 |
| BolCPIP1-3b | Bo8g117970.1 | C8 | 41546818 | 41546913 |
| BolCPIP1-4b | Bo9g002310.1 | C9 | 207929 | 208024 |
| BolCTIP1-3a | Bo9g004030.1 | C9 | 592943 | 593701 |
| BolCPIP2-4a | Bo9g144830.1 | C9 | 43533776 | 43533889 |
| BolCSIP1-2a | Bo9g157290.1 | C9 | 46799284 | 46799679 |
|  |  |  |  |  |
| ***Capsella rubella*** |  |  |  |  |
| CruNIP1-1 | Carubv10005365m | scaffold_7 | 8373719 | 8375795 |
| CruNIP1-2 | Carubv10005388m | scaffold_7 | 8432716 | 8435210 |
| CruNIP2-1 | Carubv10025497m | scaffold_4 | 9430420 | 9431586 |
| CruNIP3-1 | Carubv10010843m | scaffold_1 | 11155314 | 11156670 |
| CruNIP4-1 | Carubv10006501m | scaffold_7 | 14990411 | 14992850 |
| CruNIP4-2 | Carubv10007677m | scaffold_7 | 14993735 | 14995150 |
| CruNIP5-1 | Carubv10001558m | scaffold_6 | 13145818 | 13150232 |
| CruNIP6-1 | Carubv10021476m | scaffold_2 | 13751685 | 13753211 |
| CruNIP7-1 | Carubv10015668m | scaffold_3 | 1876435 | 1877813 |
| CruPIP1-1 | Carubv10017571m | scaffold_5 | 12960098 | 12962255 |
| CruPIP1-2 | Carubv10023782m | scaffold_4 | 14200059 | 14201763 |
| CruPIP1-3 | Carubv10009929m | scaffold_1 | 198824 | 200288 |
| CruPIP1-4 | Carubv10001627m | scaffold_6 | 16456550 | 16458865 |
| CruPIP1-5 | Carubv10005414m | scaffold_7 | 6656810 | 6658648 |
| CruPIP2-1 | Carubv10017763m | scaffold_5 | 9946078 | 9948174 |
| CruPIP2-2 | Carubv10023789m | scaffold_4 | 10631316 | 10632933 |
| CruPIP2-3 | Carubv10025207m | scaffold_4 | 10636042 | 10637185 |
| CruPIP2-4 | Carubv10026881m | scaffold_8 | 10628829 | 10630430 |
| CruPIP2-5a | Carubv10017773m | scaffold_5 | 10476180 | 10478164 |
| CruPIP2-5b | Carubv10017830m | scaffold_5 | 10476097 | 10478164 |
| CruPIP2-6 | Carubv10023768m | scaffold_4 | 11371957 | 11374331 |
| CruPIP2-7 | Carubv10005443m | scaffold_7 | 1949120 | 1950873 |
| CruPIP2-8 | Carubv10014353m | scaffold_3 | 12988513 | 12990232 |
| CruSIP1-1 | Carubv10014512m | scaffold_3 | 1099849 | 1102301 |
| CruSIP1-2 | Carubv10001770m | scaffold_6 | 6107617 | 6109139 |
| CruSIP2-1 | Carubv10018383m | scaffold_5 | 11325227 | 11326368 |
| CruTIP1-1 | Carubv10023912m | scaffold_4 | 10444341 | 10445629 |
| CruTIP1-2 | Carubv10017730m | scaffold_5 | 1807270 | 1808718 |
| CruTIP1-3 | Carubv10003767m | scaffold_6 | 16044706 | 16045464 |
| CruTIP2-1a | Carubv10011466m | scaffold_1 | 18172533 | 18173726 |
| CruTIP2-1b | Carubv10014472m | scaffold_3 | 5636018 | 5637878 |
| CruTIP2-2 | Carubv10005581m | scaffold_7 | 9120086 | 9121273 |
| CruTIP2-3 | Carubv10028328m | scaffold_8 | 101729 | 102692 |
| CruTIP3-1 | Carubv10022350m | scaffold_2 | 11094500 | 11095520 |
| CruTIP3-2 | Carubv10012552m | scaffold_1 | 6166981 | 6168059 |
| CruTIP4-1 | Carubv10023921m | scaffold_4 | 2666248 | 2668013 |
| CruTIP5-1 | Carubv10017883m | scaffold_5 | 7529378 | 7530360 |
| ***Capsella grandiflora*** |  |  |  |  |
| Cagra-NIP1-1 | Cagra.9536s0015.1 | Scaffold9536 | 50343 | 52404 |
| Cagra-NIP1-2 | Cagra.1164s0001.1 | Scaffold1164 | 87 | 2631 |
| Cagra-NIP2-1 | Cagra.7352s0012.1 | Scaffold7352 | 27126 | 28292 |
| Cagra-NIP3-1 | Cagra.1508s0012.1 | Scaffold1508 | 57150 | 58517 |
| Cagra-NIP4-1 | Cagra.8123s0002.1 | Scaffold8123 | 6883 | 9659 |
| Cagra-NIP4-2 | Cagra.8123s0003.1 | Scaffold8123 | 10590 | 11947 |
| Cagra-NIP5-1 | Cagra.2941s0001.1 | Scaffold2941 | 7542 | 11827 |
| Cagra-NIP6-1 | Cagra.1725s0064.1 | Scaffold1725 | 252758 | 254399 |
| Cagra-NIP7-1 | Cagra.1772s0034.1 | Scaffold1772 | 102430 | 103808 |
| Cagra-PIP1-1 | Cagra.3373s0010.1 | Scaffold3373 | 32290 | 33953 |
| Cagra-PIP1-2 | Cagra.0050s0101.1 | Scaffold50 | 398720 | 400266 |
| Cagra-PIP1-3 | Cagra.1968s0102.1 | Scaffold1968 | 345153 | 346531 |
| Cagra-PIP1-4 | Cagra.4456s0018.1 | Scaffold4456 | 85656 | 87252 |
| Cagra-PIP1-5 | Cagra.6664s0002.1 | Scaffold6664 | 3916 | 5411 |
| Cagra-PIP2-1 | Cagra.0612s0068.1 | Scaffold612 | 238676 | 240199 |
| Cagra-PIP2-2 | Cagra.0666s0028.1 | Scaffold666 | 107139 | 108546 |
| Cagra-PIP2-3 | Cagra.0666s0027.1 | Scaffold666 | 102745 | 104131 |
| Cagra-PIP2-4 | Cagra.2519s0037.1 | Scaffold2519 | 114223 | 115795 |
| Cagra-PIP2-5 | Cagra.1742s0040.1 | Scaffold1742 | 164983 | 165944 |
| Cagra-PIP2-6 | Cagra.0716s0018.1 | Scaffold716 | 68329 | 70587 |
| Cagra-PIP2-7 | Cagra.2350s0056.1 | Scaffold2350 | 206971 | 208596 |
| Cagra-PIP2-8 | Cagra.10374s0003.1 | Scaffold10374 | 10923 | 12151 |
| Cagra-SIP1-1 | Cagra.4031s0058.1 | Scaffold4031 | 179243 | 181567 |
| Cagra-SIP1-2 | Cagra.0906s0024.1 | Scaffold906 | 80745 | 82214 |
| Cagra-SIP2-1 | Cagra.0578s0025.1 | Scaffold578 | 78353 | 79614 |
| Cagra-TIP1-1 | Cagra.2145s0005.1 | Scaffold2145 | 24011 | 25103 |
| Cagra-TIP1-3 | Cagra.23502s0005.1 | Scaffold23502 | 14681 | 15439 |
| Cagra-TIP2-1a | Cagra.4122s0005.1 | Scaffold4122 | 18545 | 19714 |
| Cagra-TIP2-1b | Cagra.6624s0017.1 | Scaffold6624 | 67568 | 69002 |
| Cagra-TIP2-2 | Cagra.3356s0025.1 | Scaffold3356 | 99055 | 99900 |
| Cagra-TIP2-3 | Cagra.1415s0008.1 | Scaffold1415 | 27751 | 28714 |
| Cagra-TIP3-1 | Cagra.4894s0024.1 | Scaffold4894 | 112050 | 113105 |
| Cagra-TIP3-2 | Cagra.2175s0014.1 | Scaffold2175 | 57707 | 58936 |
| Cagra-TIP4-1 | Cagra.8309s0001.1 | Scaffold8309 | 469 | 2007 |
| Cagra-TIP5-1 | Cagra.18459s0002.1 | Scaffold18459 | 7160 | 8122 |
| ***Eutrema salsugineum*** |  |  |  |  |
| EsaNIP1-2 | Thhalv10025855m | scaffold_1 | 9110383 | 9112814 |
| EsaNIP2-1 | Thhalv10017039m | scaffold_10 | 7725918 | 7727077 |
| EsaNIP3-1 | Thhalv10008205m | scaffold_5 | 3238826 | 3240751 |
| EsaNIP4-1 | Thhalv10027876m | scaffold_14 | 6390951 | 6392527 |
| EsaNIP4-2 | Thhalv10028080m | scaffold_14 | 6419094 | 6420916 |
| EsaNIP4-3 | Thhalv10028303m | scaffold_14 | 6387710 | 6389411 |
| EsaNIP5-1 | Thhalv10028842m | scaffold_3 | 6965239 | 6968971 |
| EsaNIP6-1 | Thhalv10018924m | scaffold_9 | 377973 | 380029 |
| EsaNIP7-1 | Thhalv10021311m | scaffold_13 | 7524138 | 7526227 |
| EsaPIP1-1a | Thhalv10006141m | scaffold_19 | 744384 | 746111 |
| EsaPIP1-1b | Thhalv10006184m | scaffold_19 | 744384 | 746111 |
| EsaPIP1-2 | Thhalv10001573m | scaffold_22 | 1050382 | 1052181 |
| EsaPIP1-3 | Thhalv10008397m | scaffold_5 | 15430227 | 15431877 |
| EsaPIP1-4 | Thhalv10028860m | scaffold_3 | 178276 | 180388 |
| EsaPIP1-5 | Thhalv10025910m | scaffold_1 | 7168845 | 7170395 |
| EsaPIP2-1 | Thhalv10010598m | scaffold_16 | 1094038 | 1096126 |
| EsaPIP2-2 | Thhalv10017041m | scaffold_10 | 9086411 | 9087865 |
| EsaPIP2-3 | Thhalv10017874m | scaffold_10 | 9081920 | 9083065 |
| EsaPIP2-4 | Thhalv10014299m | scaffold_2 | 7963987 | 7965583 |
| EsaPIP2-5 | Thhalv10010603m | scaffold_16 | 517287 | 519747 |
| EsaPIP2-6 | Thhalv10017026m | scaffold_10 | 9992094 | 9995122 |
| EsaPIP2-7 | Thhalv10025940m | scaffold_1 | 2095242 | 2097067 |
| EsaPIP2-8 | Thhalv10026091m | scaffold_1 | 2095242 | 2097067 |
| EsaSIP1-1 | Thhalv10021302m | scaffold_13 | 8346662 | 8349497 |
| EsaSIP1-2 | Thhalv10014528m | scaffold_2 | 6226535 | 6228035 |
| EsaSIP2-1 | Thhalv10006214m | scaffold_19 | 2378143 | 2380165 |
| EsaTIP1-1 | Thhalv10017134m | scaffold_10 | 8855693 | 8856961 |
| EsaTIP1-2 | Thhalv10004806m | scaffold_6 | 6376523 | 6378262 |
| EsaTIP1-3 | Thhalv10028907m | scaffold_3 | 662302 | 663272 |
| EsaTIP2-1a | Thhalv10011730m | scaffold_7 | 1775535 | 1777275 |
| EsaTIP2-1b | Thhalv10021303m | scaffold_13 | 3845309 | 3847303 |
| EsaTIP2-2 | Thhalv10026069m | scaffold_1 | 10105540 | 10106668 |
| EsaTIP2-3 | Thhalv10001007m | scaffold_20 | 2374013 | 2375106 |
| EsaTIP3-1 | Thhalv10019492m | scaffold_9 | 3258249 | 3259349 |
| EsaTIP3-2 | Thhalv10008480m | scaffold_5 | 9379862 | 9381166 |
| EsaTIP4-1 | Thhalv10002084m | scaffold_12 | 7771337 | 7773079 |
| EsaTIP5-1 | Thhalv10011032m | scaffold_16 | 4433343 | 4434299 |

**Supplementary Table 5**. Details of isoelectric point and molecular weight of aquaporins identified in eight Brassicaceae species

| **Sr. No.** | **Gene ID** | **Isoelectric point of protein** | **Protein mass (KDa)** |
| --- | --- | --- | --- |
| 1 | AlNIP1-1 | 6.7 | 31.46 |
| 2 | AlNIP1-2 | 7.46 | 31.72 |
| 3 | AlNIP2-1 | 6.13 | 27.33 |
| 4 | AlNIP3-1 | 7.09 | 28.66 |
| 5 | AlNIP4-1 | 7.63 | 30.29 |
| 6 | AlNIP4-2 | 7.88 | 30.21 |
| 7 | AlNIP5-1 | 7.66 | 31.43 |
| 8 | AlNIP6-1a | 6.28 | 25.01 |
| 9 | AlNIP6-1b | 7.6 | 31.85 |
| 10 | AlNIP7-1 | 5.74 | 28.78 |
| 11 | AlPIP1-1 | 8.26 | 30.67 |
| 12 | AlPIP1-2 | 8.42 | 30.60 |
| 13 | AlPIP1-3 | 8.26 | 30.61 |
| 14 | AlPIP1-4 | 8.23 | 30.73 |
| 15 | AlPIP1-5 | 8.39 | 30.59 |
| 16 | AlPIP2-1 | 7.77 | 30.45 |
| 17 | AlPIP2-2a | 6.66 | 30.42 |
| 18 | AlPIP2-2b | 8.45 | 28.24 |
| 19 | AlPIP2-4 | 7.01 | 30.20 |
| 20 | AlPIP2-5 | 8.26 | 30.98 |
| 21 | AlPIP2-6 | 7.46 | 31.10 |
| 22 | AlPIP2-7 | 8.2 | 29.78 |
| 23 | AlPIP2-8 | 7.78 | 29.64 |
| 24 | AlSIP1-1 | 9.42 | 25.65 |
| 25 | AlSIP1-2 | 9.71 | 26.01 |
| 26 | AlSIP2-1 | 9 | 25.97 |
| 27 | AlTIP1-1 | 5.76 | 25.62 |
| 28 | AlTIP1-2 | 4.79 | 25.85 |
| 29 | AlTIP1-3 | 5.36 | 25.83 |
| 30 | AlTIP2-1a | 5.06 | 25.35 |
| 31 | AlTIP2-1b | 5.09 | 25.04 |
| 32 | AlTIP2-2 | 4.61 | 25.11 |
| 33 | AlTIP2-3a | 4.87 | 25.33 |
| 34 | AlTIP2-3b | 7.24 | 38.27 |
| 35 | AlTIP3-1 | 7.17 | 28.28 |
| 36 | AlTIP3-2 | 6.26 | 28.24 |
| 37 | AlTIP4-1 | 5.48 | 26.01 |
| 38 | AlTIP5-1 | 7.24 | 26.60 |
| 39 | AtNIP1-1 | 6.01 | 31.71 |
| 40 | AtNIP1-2 | 7.76 | 31.27 |
| 41 | AtNIP2-1 | 6.46 | 30.58 |
| 42 | AtNIP3-1 | 5.41 | 34.58 |
| 43 | AtNIP4-1 | 7.87 | 30.22 |
| 44 | AtNIP4-2 | 7.34 | 30.28 |
| 45 | AtNIP5-1 | 7.66 | 31.49 |
| 46 | AtNIP6-1 | 7.47 | 31.84 |
| 47 | AtNIP7-1 | 5.74 | 28.75 |
| 48 | AtPIP1-1 | 8.42 | 30.69 |
| 49 | AtPIP1-2 | 8.42 | 30.60 |
| 50 | AtPIP1-3 | 8.26 | 30.63 |
| 51 | AtPIP1-4 | 8.23 | 30.69 |
| 52 | AtPIP1-5 | 8.2 | 30.65 |
| 53 | AtPIP2-1 | 7.77 | 30.47 |
| 54 | AtPIP2-2 | 7.11 | 30.45 |
| 55 | AtPIP2-3 | 7.12 | 30.43 |
| 56 | AtPIP2-4 | 7.34 | 30.95 |
| 57 | AtPIP2-5 | 8.26 | 30.59 |
| 58 | AtPIP2-6 | 7.46 | 31.05 |
| 59 | AtPIP2-7 | 8.2 | 29.74 |
| 60 | AtPIP2-8 | 8.29 | 29.50 |
| 61 | AtSIP1-1 | 9.42 | 25.71 |
| 62 | AtSIP1-2 | 9.68 | 26.04 |
| 63 | AtSIP2-1 | 8.72 | 28.52 |
| 64 | AtTIP1-1 | 5.76 | 25.62 |
| 65 | AtTIP1-2 | 4.79 | 25.85 |
| 66 | AtTIP1-3 | 5.19 | 25.91 |
| 67 | AtTIP2-1 | 5.09 | 25.03 |
| 68 | AtTIP2-2 | 4.61 | 25.08 |
| 69 | AtTIP2-3 | 5.07 | 25.25 |
| 70 | AtTIP3-1 | 7.17 | 28.31 |
| 71 | AtTIP3-2 | 6.26 | 28.18 |
| 72 | AtTIP4-1 | 5.48 | 26.07 |
| 73 | AtTIP5-1 | 7.71 | 26.64 |
| 74 | BnaANIP1-2a | 7.26 | 31.56 |
| 75 | BnaANIP1-2b | 7.74 | 31.67 |
| 76 | BnaANIP2-1a | 7.35 | 30.25 |
| 77 | BnaANIP2-1b | 8.04 | 30.35 |
| 78 | BnaANIP3-1a | 7.5 | 30.21 |
| 79 | BnaANIP3-1b | 7.58 | 33.56 |
| 80 | BnaANIP3-1c | 8.14 | 32.10 |
| 81 | BnaANIP4-1a | 7.34 | 30.24 |
| 82 | BnaANIP4-1b | 9.29 | 24.39 |
| 83 | BnaANIP5-1a | 7.66 | 31.14 |
| 84 | BnaANIP5-1b | 7.94 | 31.21 |
| 85 | BnaANIP5-1c | 8.19 | 31.28 |
| 86 | BnaANIP6-1a | 7.34 | 31.86 |
| 87 | BnaANIP6-1b | 7.6 | 31.98 |
| 88 | BnaANIP6-1c | 7.59 | 31.80 |
| 89 | BnaANIP7-1a | 6.29 | 28.60 |
| 90 | BnaAPIP1-1a | 8.89 | 23.81 |
| 91 | BnaAPIP1-1b | 8.05 | 30.64 |
| 92 | BnaAPIP1-1c | 9.06 | 21.75 |
| 93 | BnaAPIP1-2a | 8.42 | 30.52 |
| 94 | BnaAPIP1-2b | 8.42 | 30.53 |
| 95 | BnaAPIP1-2c | 8.42 | 30.54 |
| 96 | BnaAPIP1-3a | 8.26 | 30.54 |
| 97 | BnaAPIP1-4a | 8.39 | 30.75 |
| 98 | BnaAPIP1-4b | 8.23 | 30.56 |
| 99 | BnaAPIP1-5a | 8.39 | 30.72 |
| 100 | BnaAPIP2-1a | 7.12 | 30.40 |
| 101 | BnaAPIP2-2a | 6.23 | 30.03 |
| 102 | BnaAPIP2-2b | 6.66 | 30.23 |
| 103 | BnaAPIP2-2c | 7.06 | 33.06 |
| 104 | BnaAPIP2-4a | 7.35 | 27.43 |
| 105 | BnaAPIP2-4b | 7.01 | 27.36 |
| 106 | BnaAPIP2-4c | 6.56 | 27.33 |
| 107 | BnaAPIP2-5a | 8.07 | 30.57 |
| 108 | BnaAPIP2-5b | 8.29 | 30.47 |
| 109 | BnaAPIP2-6a | 7.77 | 30.87 |
| 110 | BnaAPIP2-7a | 8.23 | 29.81 |
| 111 | BnaAPIP2-7b | 7.76 | 29.74 |
| 112 | BnaASIP1-1a | 7.85 | 31.58 |
| 113 | BnaASIP1-1b | 9.04 | 25.58 |
| 114 | BnaASIP1-2a | 10.08 | 26.21 |
| 115 | BnaASIP2-1a | 8.88 | 26.03 |
| 116 | BnaASIP2-1b | 8.66 | 25.57 |
| 117 | BnaATIP1-1a | 5.72 | 22.42 |
| 118 | BnaATIP1-1b | 5.72 | 22.42 |
| 119 | BnaATIP1-2a | 5.13 | 25.79 |
| 120 | BnaATIP1-2b | 5.38 | 25.83 |
| 121 | BnaATIP1-3a | 5.14 | 25.86 |
| 122 | BnaATIP2-1a | 5.11 | 24.99 |
| 123 | BnaATIP2-1b | 5.09 | 24.94 |
| 124 | BnaATIP2-1c | 5.09 | 24.87 |
| 125 | BnaATIP2-2a | 4.95 | 24.99 |
| 126 | BnaATIP2-3a | 4.65 | 21.31 |
| 127 | BnaATIP2-3b | 4.86 | 25.25 |
| 128 | BnaATIP3-1a | 6.53 | 28.00 |
| 129 | BnaATIP3-1b | 6.53 | 27.85 |
| 130 | BnaATIP3-1c | 6.26 | 27.98 |
| 131 | BnaATIP3-2a | 5.99 | 28.55 |
| 132 | BnaATIP3-2b | 6.27 | 28.46 |
| 133 | BnaATIP5-1a | 7.71 | 26.82 |
| 134 | BnaCNIP1-2a | 7.77 | 31.67 |
| 135 | BnaCNIP1-2b | 7.99 | 31.66 |
| 136 | BnaCNIP2-1a | 7.61 | 30.28 |
| 137 | BnaCNIP2-1b | 8.04 | 30.32 |
| 138 | BnaCNIP3-1a | 9.25 | 35.41 |
| 139 | BnaCNIP3-1b | 7.04 | 33.76 |
| 140 | BnaCNIP3-1c | 6.74 | 36.10 |
| 141 | BnaCNIP4-1a | 7.34 | 30.21 |
| 142 | BnaCNIP4-1b | 7.24 | 30.03 |
| 143 | BnaCNIP4-1c | 7.24 | 29.27 |
| 144 | BnaCNIP4-2a | 7.9 | 30.34 |
| 145 | BnaCNIP5-1a | 7.94 | 31.09 |
| 146 | BnaCNIP5-1b | 7.66 | 31.12 |
| 147 | BnaCNIP6-1a | 7.59 | 31.83 |
| 148 | BnaCNIP7-1a | 6.29 | 28.61 |
| 149 | BnaCPIP1-1a | 8.05 | 30.63 |
| 150 | BnaCPIP1-2a | 8.59 | 30.53 |
| 151 | BnaCPIP1-2b | 8.42 | 30.58 |
| 152 | BnaCPIP1-2c | 8.42 | 30.53 |
| 153 | BnaCPIP1-3a | 8.91 | 35.19 |
| 154 | BnaCPIP1-3b | 8.05 | 30.62 |
| 155 | BnaCPIP1-4a | 8.39 | 30.73 |
| 156 | BnaCPIP1-4b | 8.23 | 30.56 |
| 157 | BnaCPIP1-5a | 8.39 | 30.52 |
| 158 | BnaCPIP2-1a | 6.21 | 30.42 |
| 159 | BnaCPIP2-1b | 6.66 | 30.46 |
| 160 | BnaCPIP2-2a | 6.66 | 30.29 |
| 161 | BnaCPIP2-2b | 6.24 | 30.30 |
| 162 | BnaCPIP2-4a | 7.02 | 27.41 |
| 163 | BnaCPIP2-4b | 6.57 | 27.16 |
| 164 | BnaCPIP2-5a | 8.07 | 30.62 |
| 165 | BnaCPIP2-5b | 8.29 | 30.50 |
| 166 | BnaCPIP2-6a | 7.77 | 30.91 |
| 167 | BnaCPIP2-7a | 7.76 | 29.77 |
| 168 | BnaCPIP2-7b | 8.23 | 29.77 |
| 169 | BnaCPIP2-7c | 8.92 | 26.99 |
| 170 | BnaCSIP1-1a | 7.39 | 31.66 |
| 171 | BnaCSIP1-1b | 9.12 | 25.60 |
| 172 | BnaCSIP1-2a | 9.82 | 26.21 |
| 173 | BnaCSIP2-1a | 8.8 | 25.92 |
| 174 | BnaCSIP2-1b | 8.8 | 25.92 |
| 175 | BnaCSIP2-1c | 8.88 | 26.01 |
| 176 | BnaCTIP1-1a | 5.47 | 25.59 |
| 177 | BnaCTIP1-2a | 5.13 | 25.79 |
| 178 | BnaCTIP1-2b | 5.6 | 25.76 |
| 179 | BnaCTIP1-3a | 5.14 | 25.86 |
| 180 | BnaCTIP2-1a | 5.11 | 24.98 |
| 181 | BnaCTIP2-1b | 6.31 | 27.97 |
| 182 | BnaCTIP2-1c | 5.11 | 24.88 |
| 183 | BnaCTIP2-1d | 5.33 | 25.62 |
| 184 | BnaCTIP2-2a | 4.95 | 25.05 |
| 185 | BnaCTIP2-3a | 4.58 | 25.20 |
| 186 | BnaCTIP2-3b | 4.86 | 25.22 |
| 187 | BnaCTIP3-1a | 6.53 | 27.85 |
| 188 | BnaCTIP3-1b | 6.04 | 27.94 |
| 189 | BnaCTIP3-1c | 6.48 | 28.03 |
| 190 | BnaCTIP3-2a | 6 | 28.63 |
| 191 | BnaCTIP3-2b | 6.27 | 28.50 |
| 192 | BnaCTIP4-1a | 5.1 | 26.12 |
| 193 | BnaCTIP5-1a | 7.71 | 26.76 |
| 194 | BolCNIP1-2a | 7.77 | 31.67 |
| 195 | BolCNIP1-2b | 7.99 | 31.24 |
| 196 | BolCNIP2-1a | 8.22 | 30.41 |
| 197 | BolCNIP2-1b | 7.61 | 30.29 |
| 198 | BolCNIP3-1a | 6.41 | 34.59 |
| 199 | BolCNIP3-1b | 9.25 | 35.41 |
| 200 | BolCNIP3-1c | 6.79 | 32.21 |
| 201 | BolCNIP4-1a | 7.6 | 30.41 |
| 202 | BolCNIP4-1b | 7.24 | 30.17 |
| 203 | BolCNIP4-2 | 7.63 | 29.68 |
| 204 | BolCNIP5-1a | 7.94 | 31.09 |
| 205 | BolCNIP5-1b | 8.65 | 23.46 |
| 206 | BolCNIP6-1 | 7.59 | 31.85 |
| 207 | BolCNIP7-1 | 6.29 | 28.64 |
| 208 | BolCPIP1-1 | 8.05 | 30.63 |
| 209 | BolCPIP1-2a | 8.42 | 30.58 |
| 210 | BolCPIP1-2b | 8.42 | 30.53 |
| 211 | BolCPIP1-2c | 8.42 | 30.50 |
| 212 | BolCPIP1-3a | 8.26 | 30.62 |
| 213 | BolCPIP1-3b | 8.95 | 22.13 |
| 214 | BolCPIP1-4a | 8.52 | 30.95 |
| 215 | BolCPIP1-4b | 8.23 | 30.56 |
| 216 | BolCPIP1-5 | 8.39 | 30.51 |
| 217 | BolCPIP2-1a | 6.66 | 30.46 |
| 218 | BolCPIP2-1b | 6.66 | 30.38 |
| 219 | BolCPIP2-2a | 6.24 | 30.30 |
| 220 | BolCPIP2-2b | 6.66 | 30.29 |
| 221 | BolCPIP2-2c | 6.66 | 30.21 |
| 222 | BolCPIP2-2d | 6.23 | 30.00 |
| 223 | BolCPIP2-4a | 6.57 | 30.05 |
| 224 | BolCPIP2-4b | 7 | 30.03 |
| 225 | BolCPIP2-4c | 7.34 | 30.04 |
| 226 | BolCPIP2-5a | 8.29 | 30.50 |
| 227 | BolCPIP2-5b | 8.15 | 27.37 |
| 228 | BolCPIP2-6 | 7.77 | 30.91 |
| 229 | BolCPIP2-7a | 7.76 | 29.77 |
| 230 | BolCPIP2-7b | 8.23 | 29.83 |
| 231 | BolCPIP2-7c | 8.23 | 29.77 |
| 232 | BolCSIP1-1a | 9.12 | 25.60 |
| 233 | BolCSIP1-1b | 6.8 | 31.23 |
| 234 | BolCSIP1-2 | 10.78 | 30.39 |
| 235 | BolCSIP2-1 | 8.8 | 25.92 |
| 236 | BolCTIP1-1a | 5.76 | 25.59 |
| 237 | BolCTIP1-1b | 5.76 | 25.60 |
| 238 | BolCTIP1-2a | 5.6 | 25.76 |
| 239 | BolCTIP1-2b | 5.13 | 25.79 |
| 240 | BolCTIP1-3 | 5.14 | 25.86 |
| 241 | BolCTIP2-1a | 7.43 | 27.78 |
| 242 | BolCTIP2-1b | 6.31 | 27.97 |
| 243 | BolCTIP2-1c | 5.33 | 25.62 |
| 244 | BolCTIP2-2 | 4.95 | 25.05 |
| 245 | BolCTIP2-3a | 8.56 | 28.47 |
| 246 | BolCTIP2-3b | 4.58 | 25.20 |
| 247 | BolCTIP3-1 | 6.53 | 27.85 |
| 248 | BolCTIP3-2 | 6.21 | 28.06 |
| 249 | BolCTIP4-1 | 5.1 | 26.15 |
| 250 | BolCTIP5-1 | 7.71 | 26.81 |
| 251 | BrNIP1-2a | 7.26 | 31.64 |
| 252 | BrNIP1-2b | 7.74 | 31.69 |
| 253 | BrNIP2-1a | 7.84 | 30.38 |
| 254 | BrNIP2-1b | 7.61 | 30.32 |
| 255 | BrNIP3-1a | 6.01 | 34.55 |
| 256 | BrNIP3-1b | 7.42 | 36.08 |
| 257 | BrNIP4-1a | 7 | 29.60 |
| 258 | BrNIP4-1b | 7.34 | 30.24 |
| 259 | BrNIP5-1a | 7.66 | 31.14 |
| 260 | BrNIP5-1b | 7.94 | 31.21 |
| 261 | BrNIP6-1a | 7.05 | 31.83 |
| 262 | BrNIP6-1b | 7.59 | 31.82 |
| 263 | BrNIP7-1 | 6.29 | 28.60 |
| 264 | BrPIP1-1a | 8.05 | 30.72 |
| 265 | BrPIP1-1b | 8.46 | 30.71 |
| 266 | BrPIP1-2a | 8.02 | 30.15 |
| 267 | BrPIP1-2b | 8.26 | 30.26 |
| 268 | BrPIP1-3a | 8.26 | 30.54 |
| 269 | BrPIP1-3b | 8.26 | 30.52 |
| 270 | BrPIP1-4 | 8.39 | 30.75 |
| 271 | BrPIP1-5 | 8.2 | 30.76 |
| 272 | BrPIP2-1 | 7.12 | 30.40 |
| 273 | BrPIP2-2a | 6.66 | 30.27 |
| 274 | BrPIP2-2b | 6.23 | 30.03 |
| 275 | BrPIP2-2c | 8.46 | 24.27 |
| 276 | BrPIP2-2d | 6.56 | 25.29 |
| 277 | BrPIP2-4a | 7.34 | 29.97 |
| 278 | BrPIP2-4b | 7 | 30.12 |
| 279 | BrPIP2-4c | 6.57 | 27.29 |
| 280 | BrPIP2-5a | 8.29 | 30.47 |
| 281 | BrPIP2-5b | 7.88 | 30.49 |
| 282 | BrPIP2-6 | 7.77 | 30.87 |
| 283 | BrPIP2-7a | 8.42 | 29.71 |
| 284 | BrPIP2-7b | 7.76 | 29.74 |
| 285 | BrPIP2-7c | 7.77 | 29.80 |
| 286 | BrSIP1-1a | 9.33 | 27.24 |
| 287 | BrSIP1-1b | 9.04 | 25.61 |
| 288 | BrSIP1-2 | 9.82 | 26.18 |
| 289 | BrSIP2-1a | 8.76 | 25.90 |
| 290 | BrSIP2-1b | 8.88 | 26.03 |
| 291 | BrSIP2-1c | 8.76 | 25.49 |
| 292 | BrTIP1-1 | 5.76 | 25.55 |
| 293 | BrTIP1-2a | 5.38 | 25.77 |
| 294 | BrTIP1-2b | 5.13 | 25.79 |
| 295 | BrTIP1-3 | 5.14 | 25.86 |
| 296 | BrTIP2-1a | 5.09 | 24.86 |
| 297 | BrTIP2-1b | 5.32 | 24.89 |
| 298 | BrTIP2-1c | 5.4 | 20.98 |
| 299 | BrTIP2-2 | 4.95 | 24.99 |
| 300 | BrTIP2-3a | 4.86 | 25.25 |
| 301 | BrTIP2-3b | 4.78 | 20.68 |
| 302 | BrTIP3-1a | 6.53 | 28.00 |
| 303 | BrTIP3-1b | 6.26 | 28.00 |
| 304 | BrTIP3-2a | 6.21 | 28.58 |
| 305 | BrTIP3-2b | 6.27 | 28.46 |
| 306 | BrTIP4-1 | 5.08 | 26.09 |
| 307 | BrTIP5-1 | 5.82 | 26.30 |
| 308 | CgrNIP1-1 | 6.66 | 31.92 |
| 309 | CgrNIP1-2 | 7.78 | 30.96 |
| 310 | CgrNIP2-1 | 6.42 | 30.24 |
| 311 | CgrNIP3-1 | 5.8 | 34.88 |
| 312 | CgrNIP4-1 | 7.64 | 30.43 |
| 313 | CgrNIP4-2 | 7.5 | 30.49 |
| 314 | CgrNIP5-1 | 7.38 | 31.52 |
| 315 | CgrNIP6-1 | 7.83 | 31.96 |
| 316 | CgrNIP7-1 | 5.74 | 28.79 |
| 317 | CgrPIP1-1 | 8.26 | 30.65 |
| 318 | CgrPIP1-2 | 8.42 | 30.57 |
| 319 | CgrPIP1-3 | 8.26 | 30.65 |
| 320 | CgrPIP1-4 | 8.2 | 30.64 |
| 321 | CgrPIP1-5 | 8.2 | 30.59 |
| 322 | CgrPIP2-1 | 7.1 | 26.02 |
| 323 | CgrPIP2-2 | 6.22 | 30.35 |
| 324 | CgrPIP2-3 | 6.16 | 24.59 |
| 325 | CgrPIP2-4 | 7.33 | 30.05 |
| 326 | CgrPIP2-5 | 7.1 | 22.23 |
| 327 | CgrPIP2-6 | 7.46 | 31.25 |
| 328 | CgrPIP2-7 | 8.01 | 29.78 |
| 329 | CgrPIP2-8 | 7.98 | 25.42 |
| 330 | CgrSIP1-1 | 9.42 | 25.55 |
| 331 | CgrSIP1-2 | 9.33 | 26.08 |
| 332 | CgrSIP2-1 | 8.88 | 25.74 |
| 333 | CgrTIP1-1 | 5.76 | 25.62 |
| 334 | CgrTIP1-3 | 5.19 | 25.92 |
| 335 | CgrTIP2-1a | 5.2 | 25.93 |
| 336 | CgrTIP2-1b | 5.09 | 24.99 |
| 337 | CgrTIP2-3 | 4.87 | 25.31 |
| 338 | CgrTIP3-1 | 7.15 | 27.96 |
| 339 | CgrTIP3-2 | 6.26 | 28.29 |
| 340 | CgrTIP4-1 | 5.34 | 26.16 |
| 341 | CgrTIP5-1 | 7.71 | 26.61 |
| 342 | CruNIP1-1 | 6.66 | 31.79 |
| 343 | CruNIP1-2 | 7.78 | 31.03 |
| 344 | CruNIP2-1 | 7.07 | 30.11 |
| 345 | CruNIP3-1 | 5.66 | 34.80 |
| 346 | CruNIP4-1 | 7.64 | 30.43 |
| 347 | CruNIP4-2 | 7.25 | 29.09 |
| 348 | CruNIP5-1 | 7.38 | 31.55 |
| 349 | CruNIP6-1 | 7.81 | 31.93 |
| 350 | CruNIP7-1 | 5.74 | 28.79 |
| 351 | CruPIP1-1 | 8.87 | 37.21 |
| 352 | CruPIP1-2 | 8.42 | 30.57 |
| 353 | CruPIP1-3 | 8.26 | 30.65 |
| 354 | CruPIP1-4 | 8.2 | 30.64 |
| 355 | CruPIP1-5 | 8.2 | 30.59 |
| 356 | CruPIP2-1 | 7.76 | 30.48 |
| 357 | CruPIP2-2 | 6.22 | 30.35 |
| 358 | CruPIP2-3 | 6.66 | 30.22 |
| 359 | CruPIP2-4 | 7.59 | 31.20 |
| 360 | CruPIP2-5a | 8.26 | 30.65 |
| 361 | CruPIP2-5b | 7.76 | 29.20 |
| 362 | CruPIP2-6 | 7.46 | 31.25 |
| 363 | CruPIP2-7 | 8.01 | 29.78 |
| 364 | CruPIP2-8 | 8.46 | 29.48 |
| 365 | CruSIP1-1 | 9.42 | 25.55 |
| 366 | CruSIP1-2 | 9.85 | 27.78 |
| 367 | CruSIP2-1 | 8.88 | 25.76 |
| 368 | CruTIP1-1 | 5.76 | 25.62 |
| 369 | CruTIP1-2 | 4.96 | 25.88 |
| 370 | CruTIP1-3 | 5.19 | 25.92 |
| 371 | CruTIP2-1a | 5.2 | 25.93 |
| 372 | CruTIP2-1b | 5.09 | 25.03 |
| 373 | CruTIP2-2 | 4.61 | 25.10 |
| 374 | CruTIP2-3 | 4.87 | 25.30 |
| 375 | CruTIP3-1 | 7.15 | 27.97 |
| 376 | CruTIP3-2 | 6.26 | 28.30 |
| 377 | CruTIP4-1 | 5.24 | 26.14 |
| 378 | CruTIP5-1 | 7.71 | 26.61 |
| 379 | EsaNIP1-2 | 7.99 | 31.80 |
| 380 | EsaNIP2-1 | 6.46 | 30.56 |
| 381 | EsaNIP3-1 | 5.7 | 34.46 |
| 382 | EsaNIP4-1 | 8.1 | 30.30 |
| 383 | EsaNIP4-2 | 7.72 | 30.49 |
| 384 | EsaNIP4-3 | 7.87 | 30.34 |
| 385 | EsaNIP5-1 | 7.38 | 31.20 |
| 386 | EsaNIP6-1 | 7.59 | 31.78 |
| 387 | EsaNIP7-1 | 5.82 | 28.62 |
| 388 | EsaPIP1-1a | 8.42 | 30.77 |
| 389 | EsaPIP1-1b | 7.76 | 27.76 |
| 390 | EsaPIP1-2 | 8.42 | 30.60 |
| 391 | EsaPIP1-3 | 8.26 | 30.62 |
| 392 | EsaPIP1-4 | 8.23 | 30.56 |
| 393 | EsaPIP1-5 | 8.23 | 30.61 |
| 394 | EsaPIP2-1 | 6.66 | 30.48 |
| 395 | EsaPIP2-2 | 6.23 | 30.31 |
| 396 | EsaPIP2-3 | 6.23 | 30.21 |
| 397 | EsaPIP2-4 | 7.01 | 30.12 |
| 398 | EsaPIP2-5 | 8.05 | 30.57 |
| 399 | EsaPIP2-6 | 7.13 | 31.11 |
| 400 | EsaPIP2-7 | 8.39 | 29.82 |
| 401 | EsaPIP2-8 | 8.01 | 26.10 |
| 402 | EsaSIP1-1 | 10.56 | 30.22 |
| 403 | EsaSIP1-2 | 9.24 | 25.98 |
| 404 | EsaSIP2-1 | 8.93 | 25.85 |
| 405 | EsaTIP1-1 | 5.77 | 25.62 |
| 406 | EsaTIP1-2 | 5.13 | 25.70 |
| 407 | EsaTIP1-3 | 4.93 | 25.85 |
| 408 | EsaTIP2-1a | 5.24 | 25.85 |
| 409 | EsaTIP2-1b | 7.42 | 28.32 |
| 410 | EsaTIP2-2 | 4.74 | 25.02 |
| 411 | EsaTIP2-3 | 4.61 | 24.31 |
| 412 | EsaTIP3-1 | 7.15 | 27.94 |
| 413 | EsaTIP3-2 | 6.31 | 28.29 |
| 414 | EsaTIP4-1 | 5.24 | 26.16 |
| 415 | EsaTIP5-1 | 7.25 | 26.70 |
| 416 | PpPIP1-1 | 8.05 | 31.24 |

**Supplementary Table 6**. Details of NPA motifs, Ar/R selectivity filters, spacing between NPA domains (NPA-NPA), and number of transmembrane domains in aquaporins identified in *Arabidopsis lyrata* and *Arabidopsis thaliana*. NPA motifs were located using CDD search formed at NCBI database and transmembrane domains were identified using TMHMM.

|  |  |  |  | **Ar/R filters** | | | |  |  |  |  |
| --- | --- | --- | --- | --- | --- | --- | --- | --- | --- | --- | --- |
| **Gene_name** | **Gene_Id** | **NPA**  **(LB)** | **NPA**  **(LE)** | **H2** | **H5** | **LE1** | **LE2** | **NPA-NPA Distance** | **Specificity** | **Motif features**  **in target** | **Motif features**  **in query** |
| ***Arabidopsis lyrata*** |  |  |  |  |  |  |  |  |  |  |  |
| 492953 | AlNIP1-1 | NPA(113) | NPG(232) | W | V | A | R | 116 | specific | 6 | 6 |
| 492969 | AlNIP1-2 | NPA(115) | NPG(234) | W | V | A | R | 116 | specific | 6 | 6 |
| 869754 | AlNIP2-1 | NPA(101) | NPA(195) | V | V | A | R | 91 | generic | 6 | 6 |
| 881482 | AlNIP3-1 | NPA(48) | NPA(167) | W | I | A | R | 116 | specific | 6 | 6 |
| 330314 | AlNIP4-1 | NPA(102) | NPA(214) | W | V | A | R | 109 | specific | 6 | 6 |
| 859894 | AlNIP4-2 | NPA(102) | NPA(214) | W | V | A | R | 109 | specific | 6 | 6 |
| 911337 | AlNIP5-1 | NPS(137) | NPV(248) | A | I | G | R | 108 | specific | 6 | 6 |
| 477170 | AlNIP6-1b | NPA(139) | NPV(250) | A | I | A | R | 108 | specific | 6 | 6 |
| 477911 | AlNIP7-1 | NPS(105) | NPA(217) | A | V | G | R | 109 | specific | 6 | 6 |
| 486618 | AlPIP1-1 | NPA(114) | NPA(235) | F | H | T | R | 118 | specific | 6 | 6 |
| 483737 | AlPIP1-2 | NPA(114) | NPA(235) | F | H | T | R | 118 | specific | 6 | 6 |
| 470115 | AlPIP1-3 | NPA(114) | NPA(235) | F | H | T | R | 118 | specific | 6 | 6 |
| 912203 | AlPIP1-4 | NPA(115) | NPA(236) | F | H | T | R | 118 | specific | 6 | 6 |
| 492536 | AlPIP1-5 | NPA(115) | NPA(236) | F | H | T | R | 118 | specific | 6 | 6 |
| 485715 | AlPIP2-1 | NPA(107) | NPA(228) | F | H | T | R | 118 | specific | 6 | 6 |
| 482707 | AlPIP2-2a | NPA(105) | NPA(226) | F | H | T | R | 118 | specific | 6 | 6 |
| 902853 | AlPIP2-2b | NPA(81) | NPA(202) | K | H | T | R | 118 | specific | 6 | 6 |
| 919301 | AlPIP2-4 | NPA(107) | NPA(228) | F | H | T | R | 118 | specific | 6 | 6 |
| 485893 | AlPIP2-5 | NPA(106) | NPA(230) | F | H | T | R | 121 | specific | 6 | 6 |
| 482920 | AlPIP2-6 | NPA(106) | NPA(227) | F | H | T | R | 118 | specific | 6 | 6 |
| 491143 | AlPIP2-7 | NPA(100) | NPA(221) | F | H | T | R | 118 | specific | 6 | 6 |
| 480563 | AlPIP2-8 | NPA(98) | NPA(219) | F | H | T | R | 118 | specific | 6 | 6 |
| 907149 | AlSIP2-1 | NPL(69) | NPA(180) | S | H | G | A | 108 | generic | 6 | 6 |
| 482663 | AlTIP1-1 | NPA(85) | NPA(199) | H | I | A | V | 111 | specific | 6 | 6 |
| 484417 | AlTIP1-2 | NPA(86) | NPA(200) | H | I | A | V | 111 | specific | 6 | 6 |
| 912085 | AlTIP1-3 | NPA(85) | NPA(199) | H | I | A | V | 111 | specific | 6 | 6 |
| 882866 | AlTIP2-1a | NPA(72) | NPA(190) | L | I | G | R | 115 | specific | 6 | 6 |
| 479077 | AlTIP2-1b | NPA(84) | NPA(198) | H | I | G | R | 111 | specific | 6 | 6 |
| 493143 | AlTIP2-2 | NPA(83) | NPA(197) | H | I | G | R | 111 | specific | 6 | 6 |
| 494233 | AlTIP2-3a | NPA(83) | NPA(197) | H | I | G | R | 111 | specific | 6 | 6 |
| 476450 | AlTIP3-1 | NPA(93) | NPA(207) | H | I | A | R | 111 | specific | 6 | 6 |
| 889191 | AlTIP3-2 | NPA(93) | NPA(207) | H | T | A | R | 111 | specific | 6 | 6 |
| 901244 | AlTIP4-1 | NPA(79) | NPA(193) | A | I | A | R | 111 | specific | 6 | 6 |
| 905970 | AlTIP5-1 | NPA(87) | NPA(200) | N | V | G | C | 110 | generic | 6 | 6 |
| ***Arabidopsis thaliana*** |  |  |  |  |  |  |  |  |  |  |  |
|  | AtNIP1-1 | NPA(114) | NPG(233) | W | V | A | R | 116 | specific | 6 | 6 |
|  | AtNIP1-2 | NPA(111) | NPG(230) | W | V | A | R | 116 | specific | 6 | 6 |
|  | AtNIP2-1 | NPA(106) | NPA(225) | V | V | A | R | 116 | specific | 6 | 6 |
|  | AtNIP3-1 | NPA(102) | NPA(221) | W | I | A | R | 116 | specific | 6 | 6 |
|  | AtNIP4-1 | NPA(102) | NPA(214) | W | V | A | R | 109 | specific | 6 | 6 |
|  | AtNIP4-2 | NPA(102) | NPA(214) | W | V | A | R | 109 | specific | 6 | 6 |
|  | AtNIP5-1 | NPS(137) | NPV(248) | A | I | G | R | 108 | generic | 6 | 6 |
|  | AtNIP6-1 | NPA(139) | NPV(250) | A | I | A | R | 108 | specific | 6 | 6 |
|  | AtNIP7-1 | NPS(105) | NPA(217) | A | V | G | R | 109 | specific | 6 | 6 |
|  | AtPIP1-1 | NPA(114) | NPA(235) | F | H | T | R | 118 | specific | 6 | 6 |
|  | AtPIP1-2 | NPA(114) | NPA(235) | F | H | T | R | 118 | specific | 6 | 6 |
|  | AtPIP1-3 | NPA(114) | NPA(235) | F | H | T | R | 118 | specific | 6 | 6 |
|  | AtPIP1-4 | NPA(115) | NPA(236) | F | H | T | R | 118 | specific | 6 | 6 |
|  | AtPIP1-5 | NPA(115) | NPA(236) | F | H | T | R | 118 | specific | 6 | 6 |
|  | AtPIP2-1 | NPA(107) | NPA(228) | F | H | T | R | 118 | specific | 6 | 6 |
|  | AtPIP2-2 | NPA(105) | NPA(226) | F | H | T | R | 118 | specific | 6 | 6 |
|  | AtPIP2-3 | NPA(105) | NPA(226) | F | H | T | R | 118 | specific | 6 | 6 |
|  | AtPIP2-4 | NPA(107) | NPA(228) | F | H | T | R | 118 | specific | 6 | 6 |
|  | AtPIP2-5 | NPA(106) | NPA(227) | F | H | T | R | 118 | specific | 6 | 6 |
|  | AtPIP2-6 | NPA(106) | NPA(227) | F | H | T | R | 118 | specific | 6 | 6 |
|  | AtPIP2-7 | NPA(100) | NPA(221) | F | H | T | R | 118 | specific | 6 | 6 |
|  | AtPIP2-8 | NPA(98) | NPA(219) | F | H | T | R | 118 | specific | 6 | 6 |
|  | AtSIP2-1 | NPL(69) | NPA(180) | S | H | G | A | 108 | generic | 6 | 6 |
|  | AtTIP1-1 | NPA(85) | NPA(199) | H | I | A | V | 111 | specific | 6 | 6 |
|  | AtTIP1-2 | NPA(86) | NPA(200) | H | I | A | V | 111 | specific | 6 | 6 |
|  | AtTIP1-3 | NPA(85) | NPA(199) | H | I | A | V | 111 | specific | 6 | 6 |
|  | AtTIP2-1 | NPA(83) | NPA(197) | H | I | G | R | 111 | specific | 6 | 6 |
|  | AtTIP2-2 | NPA(83) | NPA(197) | H | I | G | R | 111 | specific | 6 | 6 |
|  | AtTIP2-3 | NPA(83) | NPA(197) | H | I | G | R | 111 | specific | 6 | 6 |
|  | AtTIP3-1 | NPA(93) | NPA(207) | H | I | A | R | 111 | specific | 6 | 6 |
|  | AtTIP3-2 | NPA(93) | NPA(207) | H | I | A | R | 111 | specific | 6 | 6 |
|  | AtTIP4-1 | NPA(79) | NPA(193) | A | I | A | R | 111 | specific | 6 | 6 |
|  | AtTIP5-1 | NPA(87) | NPA(200) | N | V | G | C | 110 | generic | 6 | 6 |

**Supplementary Table 7**. Details of NPA motifs, Ar/R selectivity filters, spacing between NPA domains (NPA-NPA), and number of transmembrane domains in aquaporins identified in *Brassica* *oleracea*. NPA motifs were located using CDD search formed at NCBI database and transmembrane domains were identified using TMHMM.

|  |  |  | **Ar/R filters** | | | |  |  |  |  |
| --- | --- | --- | --- | --- | --- | --- | --- | --- | --- | --- |
| **Gene_Id** | **NPA (LB)** | **NPA (LE)** | **H2** | **H5** | **LE1** | **LE2** | **NPA-NPA**  **Distance** | **Specificity** | **Motif features**  **in target** | **Motif features**  **in query** |
| BolCNIP1-2a | NPA(114) | NPG(233) | W | V | A | R | 116 | specific | 6 | 6 |
| BolCNIP1-2b | NPA(115) | NPG(229) | W | V | A | R | 111 | specific | 6 | 6 |
| BolCNIP2-1a | NPA(104) | NPA(223) | V | V | A | R | 116 | specific | 6 | 6 |
| BolCNIP2-1b | NPA(104) | NPA(223) | V | V | A | R | 116 | specific | 6 | 6 |
| BolCNIP3-1a | NPA(102) | NPA(221) | W | I | A | R | 116 | specific | 6 | 6 |
| BolCNIP3-1b | NPA(99) | NPA(218) | W | I | A | R | 116 | specific | 6 | 6 |
| BolCNIP3-1c | NPA(102) | NPA(197) | W | I | A | R | 92 | specific | 6 | 6 |
| BolCNIP4-1a | NPA(104) | NPA(216) | W | V | A | R | 109 | specific | 6 | 6 |
| BolCNIP4-1b | NPA(102) | NPA(214) | W | V | A | R | 109 | specific | 6 | 6 |
| BolCNIP4-2 | NPA(102) | NPA(214) | W | V | A | R | 109 | specific | 6 | 6 |
| BolCNIP5-1a | NPS(134) | NPV(245) | A | I | G | R | 108 | generic | 6 | 6 |
| BolCNIP5-1b | NPS(60) | NPV(171) | A | I | A | R | 108 | generic | 6 | 6 |
| BolCNIP6-1 | NPA(139) | NPV(250) | A | I | A | R | 108 | specific | 6 | 6 |
| BolCNIP7-1 | NPS(104) | NPA(216) | A | V | G | R | 109 | generic | 6 | 6 |
| BolCPIP1-1 | NPA(114) | NPA(235) | F | H | T | R | 118 | specific | 6 | 6 |
| BolCPIP1-2a | NPA(114) | NPA(235) | F | H | T | R | 118 | specific | 6 | 6 |
| BolCPIP1-2b | NPA(114) | NPA(235) | F | H | T | R | 118 | specific | 6 | 6 |
| BolCPIP1-2c | NPA(114) | NPA(235) | F | H | T | R | 118 | specific | 6 | 6 |
| BolCPIP1-3a | NPA(114) | NPA(235) | F | H | T | R | 118 | specific | 6 | 6 |
| BolCPIP1-3b | NPA(39) | NPA(160) | F | H | T | R | 118 | specific | 6 | 6 |
| BolCPIP1-4a | NPA(114) | NPA(235) | F | H | T | R | 118 | specific | 6 | 6 |
| BolCPIP1-4b | NPA(114) | NPA(235) | F | H | T | R | 118 | specific | 6 | 6 |
| BolCPIP1-5 | NPA(115) | NPA(236) | F | H | T | R | 118 | specific | 6 | 6 |
| BolCPIP2-1a | NPA(107) | NPA(228) | F | H | T | R | 118 | specific | 6 | 6 |
| BolCPIP2-1b | NPA(107) | NPA(228) | F | H | T | R | 118 | specific | 6 | 6 |
| BolCPIP2-2a | NPA(105) | NPA(226) | F | H | T | R | 118 | specific | 6 | 6 |
| BolCPIP2-2b | NPA(105) | NPA(226) | F | H | T | R | 118 | specific | 6 | 6 |
| BolCPIP2-2c | NPA(105) | NPA(226) | F | H | T | R | 118 | specific | 6 | 6 |
| BolCPIP2-2d | NPA(105) | NPA(226) | F | H | T | R | 118 | specific | 6 | 6 |
| BolCPIP2-4a | NPA(107) | NPA(228) | F | H | T | R | 118 | specific | 6 | 6 |
| BolCPIP2-4b | NPA(107) | NPA(228) | F | H | T | R | 118 | specific | 6 | 6 |
| BolCPIP2-4c | NPA(105) | NPA(226) | F | H | T | R | 118 | specific | 6 | 6 |
| BolCPIP2-5a | NPA(106) | NPA(227) | F | H | T | R | 118 | specific | 6 | 6 |
| BolCPIP2-6 | NPA(105) | NPA(226) | F | H | T | R | 118 | specific | 6 | 6 |
| BolCPIP2-7a | NPA(101) | NPA(222) | F | H | T | R | 118 | specific | 6 | 6 |
| BolCPIP2-7b | NPA(101) | NPA(222) | F | H | T | R | 118 | specific | 6 | 6 |
| BolCPIP2-7c | NPA(101) | NPA(222) | F | H | T | R | 118 | specific | 6 | 6 |
| BolCSIP2-1 | NPL(69) | NPA(180) | S | H | G | A | 108 | generic | 6 | 6 |
| BolCTIP1-1a | NPA(85) | NPA(199) | H | I | A | V | 111 | specific | 6 | 6 |
| BolCTIP1-1b | NPA(85) | NPA(199) | H | I | A | V | 111 | specific | 6 | 6 |
| BolCTIP1-2a | NPA(86) | NPA(200) | H | I | A | V | 111 | specific | 6 | 6 |
| BolCTIP1-2b | NPA(86) | NPA(200) | H | I | A | V | 111 | specific | 6 | 6 |
| BolCTIP1-3 | NPA(85) | NPA(199) | H | I | A | V | 111 | specific | 6 | 6 |
| BolCTIP2-1a | NPA(110) | NPA(224) | H | I | G | R | 111 | specific | 6 | 6 |
| BolCTIP2-1b | NPA(109) | NPA(223) | H | I | G | R | 111 | specific | 6 | 6 |
| BolCTIP2-1c | NPA(82) | NPA(196) | H | I | G | R | 111 | specific | 6 | 6 |
| BolCTIP2-2 | NPA(83) | NPA(197) | H | I | G | R | 111 | specific | 6 | 6 |
| BolCTIP2-3a | NPA(110) | NPA(224) | H | I | G | R | 111 | specific | 6 | 6 |
| BolCTIP2-3b | NPA(83) | NPA(197) | H | I | G | R | 111 | specific | 6 | 6 |
| BolCTIP3-1 | NPA(93) | NPA(207) | H | I | A | R | 111 | specific | 6 | 6 |
| BolCTIP3-2 | NPA(93) | NPA(207) | H | I | A | R | 111 | specific | 6 | 6 |
| BolCTIP4-1 | NPA(79) | NPA(193) | A | I | A | R | 111 | specific | 6 | 6 |
| BolCTIP5-1 | NPA(87) | NPA(200) | N | V | G | C | 110 | generic | 6 | 6 |

**Supplementary Table 8**. Details of NPA motifs, Ar/R selectivity filters, spacing between NPA domains (NPA-NPA), and number of transmembrane domains in aquaporins identified in *Brassica rapa*. NPA motifs were located using CDD search formed at NCBI database and transmembrane domains were identified using TMHMM.

|  |  |  | **Ar/R filters** | | | |  |  |  |  |
| --- | --- | --- | --- | --- | --- | --- | --- | --- | --- | --- |
| **Gene_Id** | **NPA (LB)** | **NPA (LE)** | **H2** | **H5** | **LE1** | **LE2** | **NPA-NPA**  **Distance** | **Specificity** | **Motif features**  **in target** | **Motif features**  **in query** |
| BrNIP1-2a | NPA(114) | NPG(233) | W | V | A | R | 116 | specific | 6 | 6 |
| BrNIP1-2b | NPA(115) | NPG(234) | W | V | A | R | 116 | specific | 6 | 6 |
| BrNIP2-1a | NPA(105) | NPA(224) | V | V | A | R | 116 | specific | 6 | 6 |
| BrNIP2-1b | NPA(104) | NPA(223) | V | V | A | R | 116 | specific | 6 | 6 |
| BrNIP3-1a | NPA(102) | NPA(221) | W | I | A | R | 116 | specific | 6 | 6 |
| BrNIP3-1b | NPA(116) | NPA(235) | W | I | A | R | 116 | specific | 6 | 6 |
| BrNIP4-1a | NPA(102) | NPA(214) | W | V | A | R | 109 | specific | 6 | 6 |
| BrNIP4-1b | NPA(102) | NPA(214) | W | V | A | R | 109 | specific | 6 | 6 |
| BrNIP5-1a | NPS(134) | NPV(245) | A | I | G | R | 108 | generic | 6 | 6 |
| BrNIP5-1b | NPS(134) | NPV(245) | A | I | A | R | 108 | generic | 6 | 6 |
| BrNIP6-1a | NPA(139) | NPV(250) | A | I | A | R | 108 | specific | 6 | 6 |
| BrNIP6-1b | NPA(139) | NPV(250) | A | I | A | R | 108 | specific | 6 | 6 |
| BrNIP7-1 | NPS(104) | NPA(216) | A | V | G | R | 109 | generic | 6 | 6 |
| BrPIP1-1a | NPA(114) | NPA(235) | F | H | T | R | 118 | specific | 6 | 6 |
| BrPIP1-1b | NPA(114) | NPA(235) | F | H | T | R | 118 | specific | 6 | 6 |
| BrPIP1-2a | NPA(114) | NPA(229) | F | H | T | R | 112 | specific | 6 | 6 |
| BrPIP1-2b | NPA(114) | NPA(235) | F | H | T | R | 118 | specific | 6 | 6 |
| BrPIP1-3a | NPA(114) | NPA(235) | F | H | T | R | 118 | specific | 6 | 6 |
| BrPIP1-3b | NPA(114) | NPA(235) | F | H | T | R | 118 | specific | 6 | 6 |
| BrPIP1-4 | NPA(114) | NPA(235) | F | H | T | R | 118 | specific | 6 | 6 |
| BrPIP1-5 | NPA(115) | NPA(236) | F | H | T | R | 118 | specific | 6 | 6 |
| BrPIP2-1 | NPA(107) | NPA(228) | F | H | T | R | 118 | specific | 6 | 6 |
| BrPIP2-2a | NPA(105) | NPA(226) | F | H | T | R | 118 | specific | 6 | 6 |
| BrPIP2-2b | NPA(105) | NPA(226) | F | H | T | R | 118 | specific | 6 | 6 |
| BrPIP2-2c | NPA(51) | NPA(172) | F | H | T | R | 118 | specific | 6 | 6 |
| BrPIP2-4a | NPA(105) | NPA(226) | F | H | T | R | 118 | specific | 6 | 6 |
| BrPIP2-4b | NPA(107) | NPA(228) | F | H | T | R | 118 | specific | 6 | 6 |
| BrPIP2-4c | NPA(82) | NPA(203) | F | H | T | R | 118 | specific | 6 | 6 |
| BrPIP2-5a | NPA(106) | NPA(227) | F | H | T | R | 118 | specific | 6 | 6 |
| BrPIP2-5b | NPA(106) | NPA(227) | F | H | T | R | 118 | specific | 6 | 6 |
| BrPIP2-6 | NPA(105) | NPA(226) | F | H | T | R | 118 | specific | 6 | 6 |
| BrPIP2-7a | NPA(101) | NPA(222) | F | H | T | R | 118 | specific | 6 | 6 |
| BrPIP2-7b | NPA(101) | NPA(222) | F | H | T | R | 118 | specific | 6 | 6 |
| BrPIP2-7c | NPA(101) | NPA(222) | F | H | T | R | 118 | specific | 6 | 6 |
| BrSIP2-1a | NPL(69) | NPA(180) | S | H | G | A | 108 | generic | 6 | 6 |
| BrSIP2-1b | NPL(69) | NPA(180) | S | H | G | A | 108 | generic | 6 | 6 |
| BrSIP2-1c | NPV(69) | NPA(177) | S | H | G | A | 105 | generic | 6 | 6 |
| BrTIP1-1 | NPA(85) | NPA(199) | H | I | A | V | 111 | specific | 6 | 6 |
| BrTIP1-2a | NPA(86) | NPA(200) | H | I | A | V | 111 | specific | 6 | 6 |
| BrTIP1-2b | NPA(86) | NPA(200) | H | I | A | V | 111 | specific | 6 | 6 |
| BrTIP1-3 | NPA(85) | NPA(199) | H | I | A | V | 111 | specific | 6 | 6 |
| BrTIP2-1a | NPA(83) | NPA(197) | H | I | G | R | 111 | specific | 6 | 6 |
| BrTIP2-1b | NPA(83) | NPA(197) | H | I | G | R | 111 | specific | 6 | 6 |
| BrTIP2-1c | NPA(44) | NPA(158) | H | I | G | R | 111 | specific | 6 | 6 |
| BrTIP2-2 | NPA(83) | NPA(197) | H | I | G | R | 111 | specific | 6 | 6 |
| BrTIP2-3a | NPA(83) | NPA(197) | H | I | G | R | 111 | specific | 6 | 6 |
| BrTIP2-3b | NPA(37) | NPA(151) | H | I | G | R | 111 | specific | 6 | 6 |
| BrTIP3-1a | NPA(93) | NPA(207) | H | I | A | R | 111 | specific | 6 | 6 |
| BrTIP3-1b | NPA(93) | NPA(207) | H | I | A | R | 111 | specific | 6 | 6 |
| BrTIP3-2a | NPA(93) | NPA(207) | H | M | A | R | 111 | specific | 6 | 6 |
| BrTIP3-2b | NPA(93) | NPA(207) | H | M | A | R | 111 | specific | 6 | 6 |
| BrTIP4-1 | NPA(79) | NPA(193) | A | I | A | R | 111 | specific | 6 | 6 |
| BrTIP5-1 | NPA(84) | NPA(197) | N | V | G | C | 110 | generic | 6 | 6 |

**Supplementary Table 9**. Details of NPA motifs, Ar/R selectivity filters, spacing between NPA domains (NPA-NPA), and number of transmembrane domains in aquaporins identified in *Capsella grandiflora*. NPA motifs were located using CDD search formed at NCBI database and transmembrane domains were identified using TMHMM.

|  |  |  | **Ar/R filters** | | | |  |  |  |  |
| --- | --- | --- | --- | --- | --- | --- | --- | --- | --- | --- |
| **Gene_Id** | **NPA (LB)** | **NPA (LE)** | **H2** | **H5** | **LE1** | **LE2** | **NPA-NPA**  **Distance** | **Specificity** | **Motif features**  **in target** | **Motif features**  **in query** |
| CgrNIP1-1 | NPA(118) | NPG(237) | W | V | A | R | 116 | specific | 6 | 6 |
| CgrNIP1-2 | NPA(110) | NPG(229) | W | V | A | R | 116 | specific | 6 | 6 |
| CgrNIP2-1 | NPA(101) | NPA(220) | V | V | A | R | 116 | specific | 6 | 6 |
| CgrNIP3-1 | NPA(102) | NPA(221) | W | I | A | R | 116 | specific | 6 | 6 |
| CgrNIP4-1 | NPA(104) | NPA(216) | W | V | A | R | 109 | specific | 6 | 6 |
| CgrNIP4-2 | NPA(102) | NPA(214) | W | V | A | R | 109 | specific | 6 | 6 |
| CgrNIP5-1 | NPS(137) | NPV(248) | A | I | A | R | 108 | generic | 6 | 6 |
| CgrNIP6-1 | NPA(140) | NPV(251) | A | I | A | R | 108 | specific | 6 | 6 |
| CgrNIP7-1 | NPS(105) | NPA(217) | A | V | G | R | 109 | specific | 6 | 6 |
| CgrPIP1-1 | NPA(114) | NPA(235) | F | H | T | R | 118 | specific | 6 | 6 |
| CgrPIP1-2 | NPA(114) | NPA(235) | F | H | T | R | 118 | specific | 6 | 6 |
| CgrPIP1-3 | NPA(114) | NPA(235) | F | H | T | R | 118 | specific | 6 | 6 |
| CgrPIP1-4 | NPA(115) | NPA(236) | F | H | T | R | 118 | specific | 6 | 6 |
| CgrPIP1-5 | NPA(115) | NPA(236) | F | H | T | R | 118 | specific | 6 | 6 |
| CgrPIP2-1 | NPA(107) | NPA(228) | F | H | T | R | 118 | specific | 6 | 6 |
| CgrPIP2-2 | NPA(105) | NPA(226) | F | H | T | R | 118 | specific | 6 | 6 |
| CgrPIP2-4 | NPA(107) | NPA(228) | F | H | T | R | 118 | specific | 6 | 6 |
| CgrPIP2-6 | NPA(107) | NPA(228) | F | H | T | R | 118 | specific | 6 | 6 |
| CgrPIP2-7 | NPA(100) | NPA(221) | F | H | T | R | 118 | specific | 6 | 6 |
| CgrPIP2-8 | NPA(98) | NPA(219) | F | H | T | R | 118 | specific | 6 | 6 |
| CgrSIP2-1 | NPL(69) | NPA(180) | S | H | G | A | 108 | generic | 6 | 6 |
| CgrTIP1-1 | NPA(85) | NPA(199) | H | I | A | V | 111 | specific | 6 | 6 |
| CgrTIP1-3 | NPA(85) | NPA(199) | H | I | A | V | 111 | specific | 6 | 6 |
| CgrTIP2-1a | NPA(83) | NPA(197) | H | I | G | R | 111 | specific | 6 | 6 |
| CgrTIP2-1b | NPA(83) | NPA(197) | H | I | G | R | 111 | specific | 6 | 6 |
| CgrTIP2-3 | NPA(83) | NPA(197) | H | I | G | R | 111 | specific | 6 | 6 |
| CgrTIP3-1 | NPA(93) | NPA(207) | H | I | A | R | 111 | specific | 6 | 6 |
| CgrTIP3-2 | NPA(93) | NPA(207) | H | I | A | R | 111 | specific | 6 | 6 |
| CgrTIP4-1 | NPA(79) | NPA(193) | A | I | A | R | 111 | specific | 6 | 6 |
| CgrTIP5-1 | NPA(87) | NPA(200) | N | V | G | C | 110 | generic | 6 | 6 |

**Supplementary Table 10.** Details of NPA motifs, Ar/R selectivity filters, spacing between NPA domains (NPA-NPA), and number of transmembrane domains in aquaporins identified *in Capsella rubella*. NPA motifs were located using CDD search formed at NCBI database and transmembrane domains were identified using TMHMM.

|  |  |  | **Ar/R filters** | | | |  |  |  |  |
| --- | --- | --- | --- | --- | --- | --- | --- | --- | --- | --- |
| **Gene_Id** | **NPA (LB)** | **NPA (LE)** | **H2** | **H5** | **LE1** | **LE2** | **NPA-NPA**  **Distance** | **Specificity** | **Motif features**  **in target** | **Motif features**  **in query** |
| CruNIP1-1 | NPA(117) | NPG(236) | W | V | A | R | 116 | specific | 6 | 6 |
| CruNIP1-2 | NPA(110) | NPG(229) | W | V | A | R | 116 | specific | 6 | 6 |
| CruNIP2-1 | NPA(101) | NPA(220) | V | V | A | R | 116 | specific | 6 | 6 |
| CruNIP3-1 | NPA(102) | NPA(221) | W | I | A | R | 116 | specific | 6 | 6 |
| CruNIP4-1 | NPA(104) | NPA(216) | W | V | A | R | 109 | specific | 6 | 6 |
| CruNIP4-2 | NPA(102) | NPA(214) | W | V | A | R | 109 | specific | 6 | 6 |
| CruNIP5-1 | NPS(137) | NPV(248) | A | I | A | R | 108 | generic | 6 | 6 |
| CruNIP6-1 | NPA(140) | NPV(251) | A | I | A | R | 108 | specific | 6 | 6 |
| CruNIP7-1 | NPS(105) | NPA(217) | A | V | G | R | 109 | specific | 6 | 6 |
| CruPIP1-1 | NPA(171) | NPA(292) | F | H | T | R | 118 | specific | 6 | 6 |
| CruPIP1-2 | NPA(114) | NPA(235) | F | H | T | R | 118 | specific | 6 | 6 |
| CruPIP1-3 | NPA(114) | NPA(235) | F | H | T | R | 118 | specific | 6 | 6 |
| CruPIP1-4 | NPA(115) | NPA(236) | F | H | T | R | 118 | specific | 6 | 6 |
| CruPIP1-5 | NPA(115) | NPA(236) | F | H | T | R | 118 | specific | 6 | 6 |
| CruPIP2-1 | NPA(107) | NPA(228) | F | H | T | R | 118 | specific | 6 | 6 |
| CruPIP2-2 | NPA(105) | NPA(226) | F | H | T | R | 118 | specific | 6 | 6 |
| CruPIP2-3 | NPA(105) | NPA(226) | F | H | T | R | 118 | specific | 6 | 6 |
| CruPIP2-4 | NPA(119) | NPA(240) | F | H | T | R | 118 | specific | 6 | 6 |
| CruPIP2-5a | NPA(106) | NPA(227) | F | H | T | R | 118 | specific | 6 | 6 |
| CruPIP2-5b | NPA(106) | NPA(227) | F | H | T | R | 118 | specific | 6 | 6 |
| CruPIP2-6 | NPA(107) | NPA(228) | F | H | T | R | 118 | specific | 6 | 6 |
| CruPIP2-7 | NPA(100) | NPA(221) | F | H | T | R | 118 | specific | 6 | 6 |
| CruPIP2-8 | NPA(98) | NPA(219) | F | H | T | R | 118 | specific | 6 | 6 |
| CruSIP2-1 | NPL(69) | NPA(180) | S | H | G | T | 108 | generic | 6 | 6 |
| CruTIP1-1 | NPA(85) | NPA(199) | H | I | A | V | 111 | specific | 6 | 6 |
| CruTIP1-2 | NPA(86) | NPA(200) | H | I | A | V | 111 | specific | 6 | 6 |
| CruTIP1-3 | NPA(85) | NPA(199) | H | I | A | V | 111 | specific | 6 | 6 |
| CruTIP2-1a | NPA(83) | NPA(197) | H | I | G | R | 111 | specific | 6 | 6 |
| CruTIP2-1b | NPA(83) | NPA(197) | H | I | G | R | 111 | specific | 6 | 6 |
| CruTIP2-2 | NPA(83) | NPA(197) | H | I | G | R | 111 | specific | 6 | 6 |
| CruTIP2-3 | NPA(83) | NPA(197) | H | I | G | R | 111 | specific | 6 | 6 |
| CruTIP3-1 | NPA(93) | NPA(207) | H | I | A | R | 111 | specific | 6 | 6 |
| CruTIP3-2 | NPA(93) | NPA(207) | H | I | A | R | 111 | specific | 6 | 6 |
| CruTIP4-1 | NPA(79) | NPA(193) | A | I | A | R | 111 | specific | 6 | 6 |
| CruTIP5-1 | NPA(87) | NPA(200) | N | V | G | C | 110 | generic | 6 | 6 |

**Supplementary Table 11**. Details of NPA motifs, Ar/R selectivity filters, spacing between NPA domains (NPA-NPA), and number of transmembrane domains in aquaporins identified in *Eutrema salsugineum*. NPA motifs were located using CDD search formed at NCBI database and transmembrane domains were identified using TMHMM.

|  |  |  | **Ar/R filters** | | | |  |  |  |  |
| --- | --- | --- | --- | --- | --- | --- | --- | --- | --- | --- |
| **Gene_Id** | **NPA (LB)** | **NPA (LE)** | **H2** | **H5** | **LE1** | **LE2** | **NPA-NPA**  **Distance** | **Specificity** | **Motif features**  **in target** | **Motif features**  **in query** |
| EsaNIP1-2 | NPA(114) | NPG(233) | W | V | A | R | 116 | specific | 6 | 6 |
| EsaNIP2-1 | NPA(104) | NPA(223) | V | V | A | R | 116 | specific | 6 | 6 |
| EsaNIP3-1 | NPA(102) | NPA(221) | W | I | A | R | 116 | specific | 6 | 6 |
| EsaNIP4-1 | NPA(102) | NPA(214) | W | V | A | R | 109 | specific | 6 | 6 |
| EsaNIP4-2 | NPA(102) | NPA(214) | W | V | A | R | 109 | specific | 6 | 6 |
| EsaNIP4-3 | NPA(102) | NPA(214) | W | V | A | R | 109 | specific | 6 | 6 |
| EsaNIP5-1 | NPS(134) | NPV(245) | A | I | G | R | 108 | generic | 6 | 6 |
| EsaNIP6-1 | NPA(139) | NPV(250) | A | I | A | R | 108 | specific | 6 | 6 |
| EsaNIP7-1 | NPS(105) | NPA(217) | A | V | G | R | 109 | generic | 6 | 6 |
| EsaPIP1-1a | NPA(114) | NPA(235) | F | H | T | R | 118 | specific | 6 | 6 |
| EsaPIP1-2 | NPA(114) | NPA(235) | F | H | T | R | 118 | specific | 6 | 6 |
| EsaPIP1-3 | NPA(114) | NPA(235) | F | H | T | R | 118 | specific | 6 | 6 |
| EsaPIP1-4 | NPA(114) | NPA(235) | F | H | T | R | 118 | specific | 6 | 6 |
| EsaPIP1-5 | NPA(115) | NPA(236) | F | H | T | R | 118 | specific | 6 | 6 |
| EsaPIP2-1 | NPA(107) | NPA(228) | F | H | T | R | 118 | specific | 6 | 6 |
| EsaPIP2-2 | NPA(105) | NPA(226) | F | H | T | R | 118 | specific | 6 | 6 |
| EsaPIP2-3 | NPA(104) | NPA(225) | F | H | T | R | 118 | specific | 6 | 6 |
| EsaPIP2-4 | NPA(107) | NPA(228) | F | H | T | R | 118 | specific | 6 | 6 |
| EsaPIP2-5 | NPA(106) | NPA(227) | F | H | T | R | 118 | specific | 6 | 6 |
| EsaPIP2-6 | NPA(107) | NPA(228) | F | H | T | R | 118 | specific | 6 | 6 |
| EsaPIP2-7 | NPA(101) | NPA(222) | F | H | T | R | 118 | specific | 6 | 6 |
| EsaPIP2-8 | NPA(101) | NPA(222) | F | H | T | R | 118 | specific | 6 | 6 |
| EsaSIP2-1 | NPL(69) | NPA(180) | S | H | G | A | 108 | generic | 6 | 6 |
| EsaTIP1-1 | NPA(85) | NPA(199) | H | I | A | V | 111 | specific | 6 | 6 |
| EsaTIP1-2 | NPA(86) | NPA(200) | H | I | A | V | 111 | specific | 6 | 6 |
| EsaTIP1-3 | NPA(85) | NPA(199) | H | I | A | V | 111 | specific | 6 | 6 |
| EsaTIP2-1a | NPA(82) | NPA(196) | H | I | G | R | 111 | specific | 6 | 6 |
| EsaTIP2-1b | NPA(111) | NPA(225) | H | I | G | R | 111 | specific | 6 | 6 |
| EsaTIP2-2 | NPA(83) | NPA(197) | H | I | G | R | 111 | specific | 6 | 6 |
| EsaTIP2-3 | NPA(83) | NPA(197) | H | I | G | R | 111 | specific | 6 | 6 |
| EsaTIP3-1 | NPA(93) | NPA(207) | H | T | A | R | 111 | specific | 6 | 6 |
| EsaTIP3-2 | NPA(93) | NPA(207) | H | M | A | R | 111 | specific | 6 | 6 |
| EsaTIP4-1 | NPA(79) | NPA(193) | A | I | A | R | 111 | specific | 6 | 6 |
| EsaTIP5-1 | NPA(87) | NPA(200) | N | V | G | C | 110 | generic | 6 | 6 |

**Supplementary Table 12**. Details of aquaporins identified in *Brassica* and other plant species belonging to different orders.

| **Plant Species** | **Order** | **PIP** | **TIP** | **NIP** | **SIP** | **XIP** | **AQP** |
| --- | --- | --- | --- | --- | --- | --- | --- |
| *Arabidopsis thaliana* | Brassicales | 13 | 10 | 9 | 3 | 0 | 35 |
| *Arabidopsis lyrata* | Brassicales | 13 | 12 | 10 | 3 | 0 | 38 |
| *Capsella rubella* | Brassicales | 14 | 11 | 9 | 3 | 0 | 37 |
| *Capsella grandiflora* | Brassicales | 13 | 9 | 9 | 3 | 0 | 34 |
| *Brassica rapa* | Brassicales | 22 | 16 | 13 | 6 | 0 | 57 |
| *Brassica napus* | Brassicales | 43 | 35 | 31 | 11 | 0 | 120 |
| *Brassica oleracea* | Brassicales | 24 | 15 | 14 | 4 | 0 | 57 |
| *Eutrema salsugineum* | Brassicales | 14 | 11 | 9 | 3 | 0 | 37 |
| *Selaginella moellendorfﬁi #* | Selaginellales | 3 | 3 | 8 | 1 | 3 | 19 |
| *Picea abies #* | Pinales | 18 | 6 | 13 | 2 | 0 | 39 |
| *Musa acuminata #* | Zingiberales | 21 | 18 | 9 | 3 | 0 | 51 |
| *Brachypodium distachyon #* | Poales | 11 | 10 | 9 | 2 | 0 | 32 |
| *Sorghum bicolor #* | Poales | 14 | 13 | 10 | 3 | 0 | 40 |
| *Zea mays #* | Poales | 10 | 13 | 13 | 7 | 0 | 43 |
| *Setaria italica #* | Poales | 16 | 16 | 15 | 3 | 0 | 50 |
| *Elaeis guineensis #* | Arecales | 9 | 10 | 9 | 2 | 0 | 30 |
| *Carica papaya #* | Brassicales | 10 | 7 | 7 | 2 | 2 | 28 |
| *Citrus sinensis #* | Sapindales | 11 | 9 | 8 | 3 | 3 | 34 |
| *Citrus clementina #* | Sapindales | 14 | 10 | 9 | 3 | 1 | 37 |
| *Vitis vinifera #* | Vitales | 9 | 9 | 9 | 1 | 2 | 30 |
| *Glycine max #* | Fabales | 22 | 23 | 17 | 8 | 2 | 72 |
| *Cajanus cajan #* | Fabales | 12 | 13 | 10 | 4 | 1 | 40 |
| *Fragaria vesca #* | Rosales | 10 | 9 | 14 | 4 | 2 | 39 |
| *Prunus persica #* | Rosales | 7 | 8 | 9 | 3 | 2 | 29 |
| *Ricinus communis #* | Malpighiales | 10 | 9 | 8 | 4 | 5 | 36 |
| *Solanum tuberosum #* | Solanales | 15 | 11 | 11 | 2 | 5 | 44 |
| *Solanum lycopersicum #* | Solanales | 14 | 10 | 11 | 3 | 6 | 44 |

# - Data obtained from Deshmukh et al. 2015.


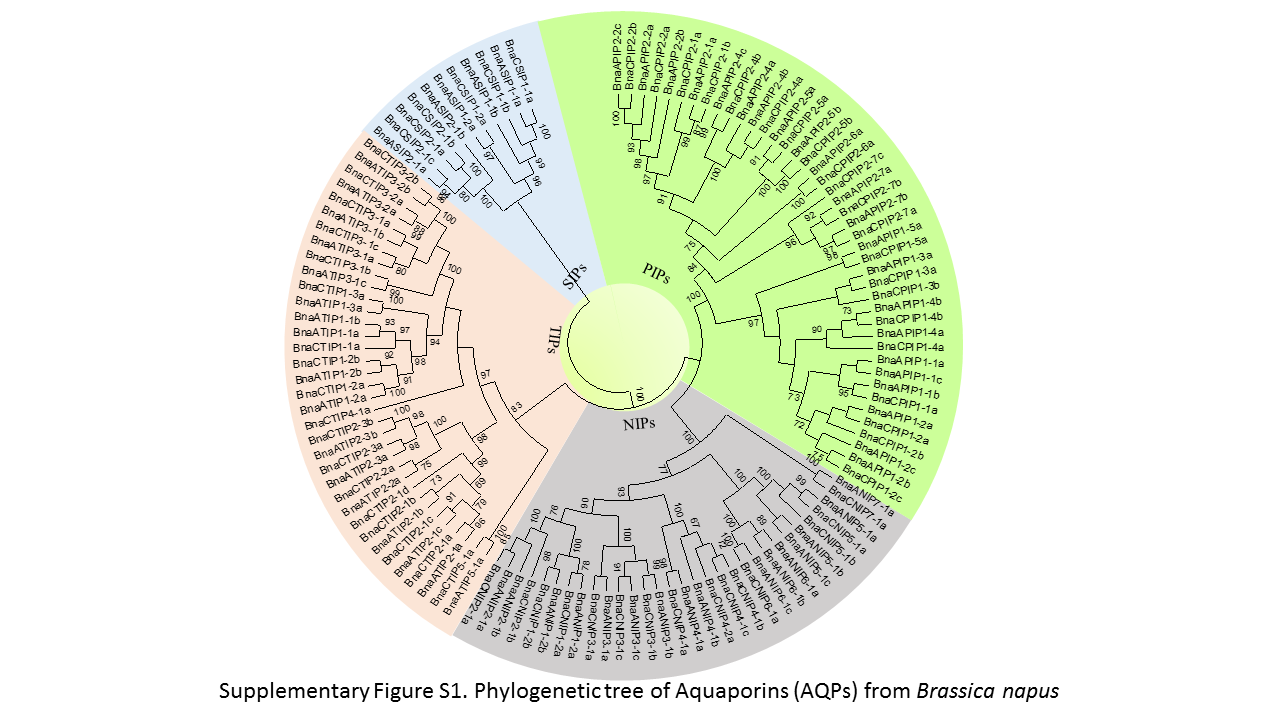


**Supplementary Figure 1** Phylogenetic tree of *Brassica* *napus* aquaporins (AQPs) showing four different sub-families


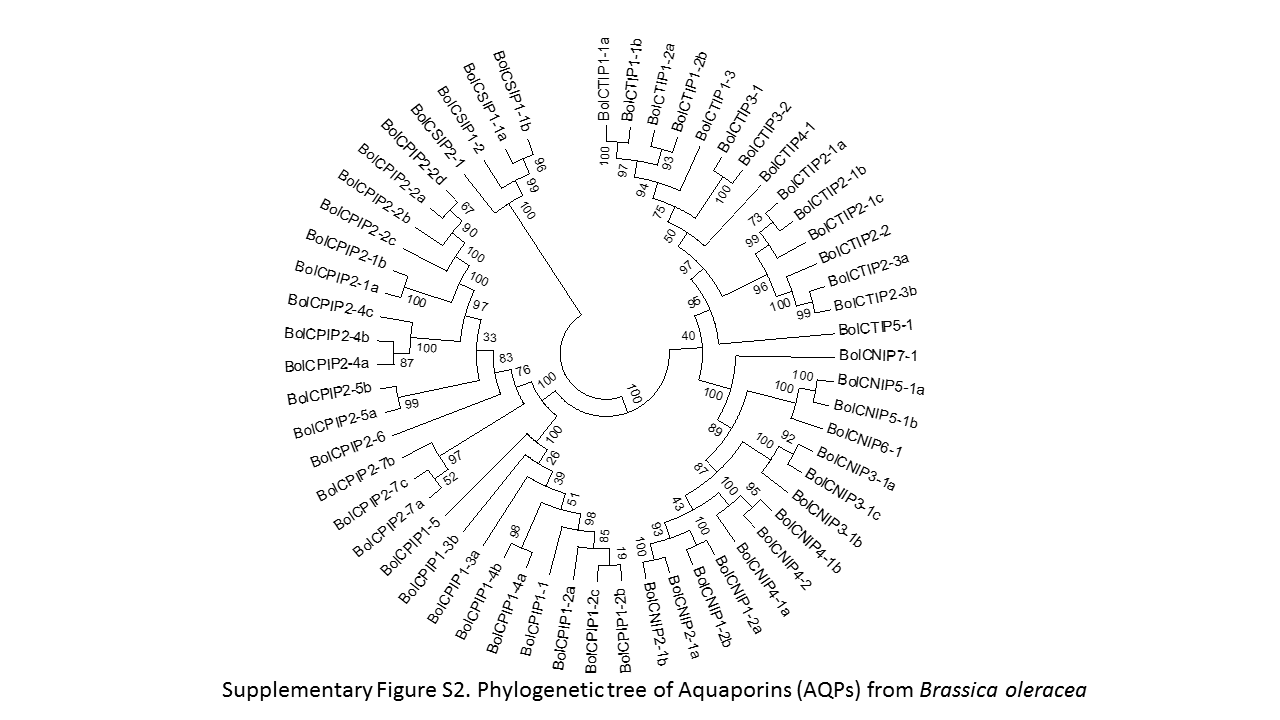


**Supplementary Figure 2** Phylogenetic tree of *Brassica* *oleracea* aquaporins (AQPs)


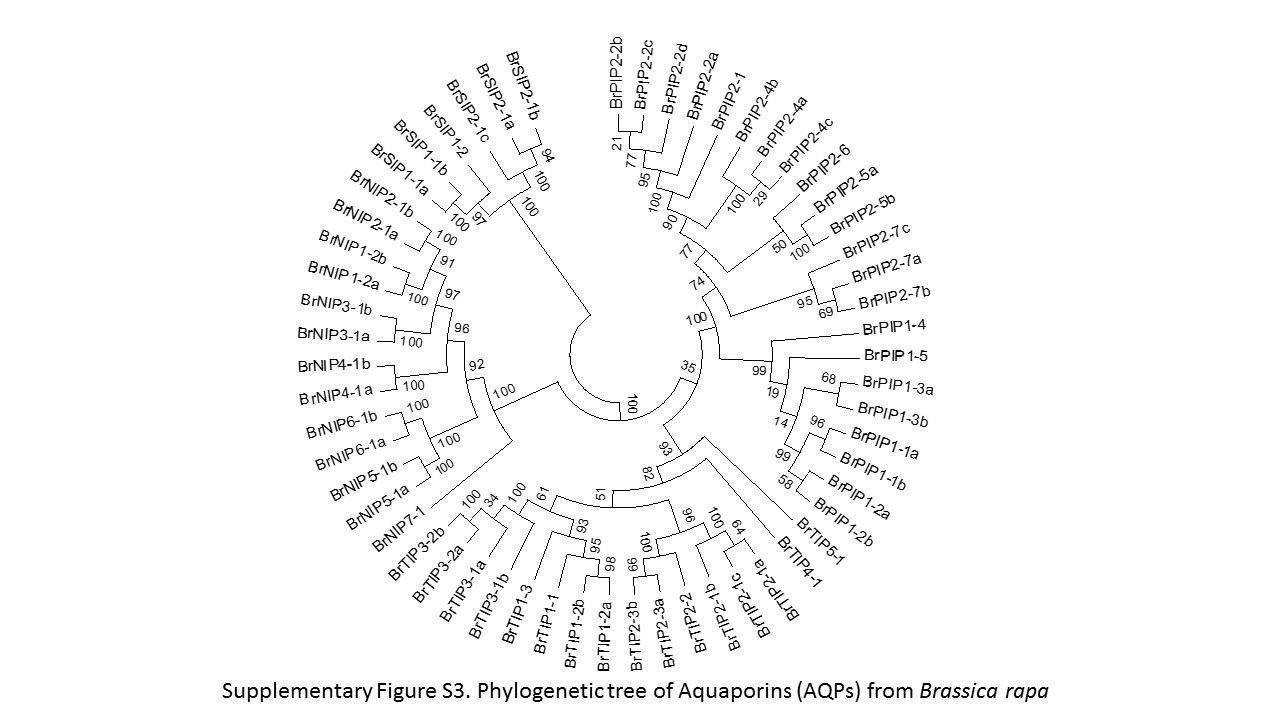


**Supplementary Figure 3** Phylogenetic tree of *Brassica* *rapa* aquaporins (AQPs)


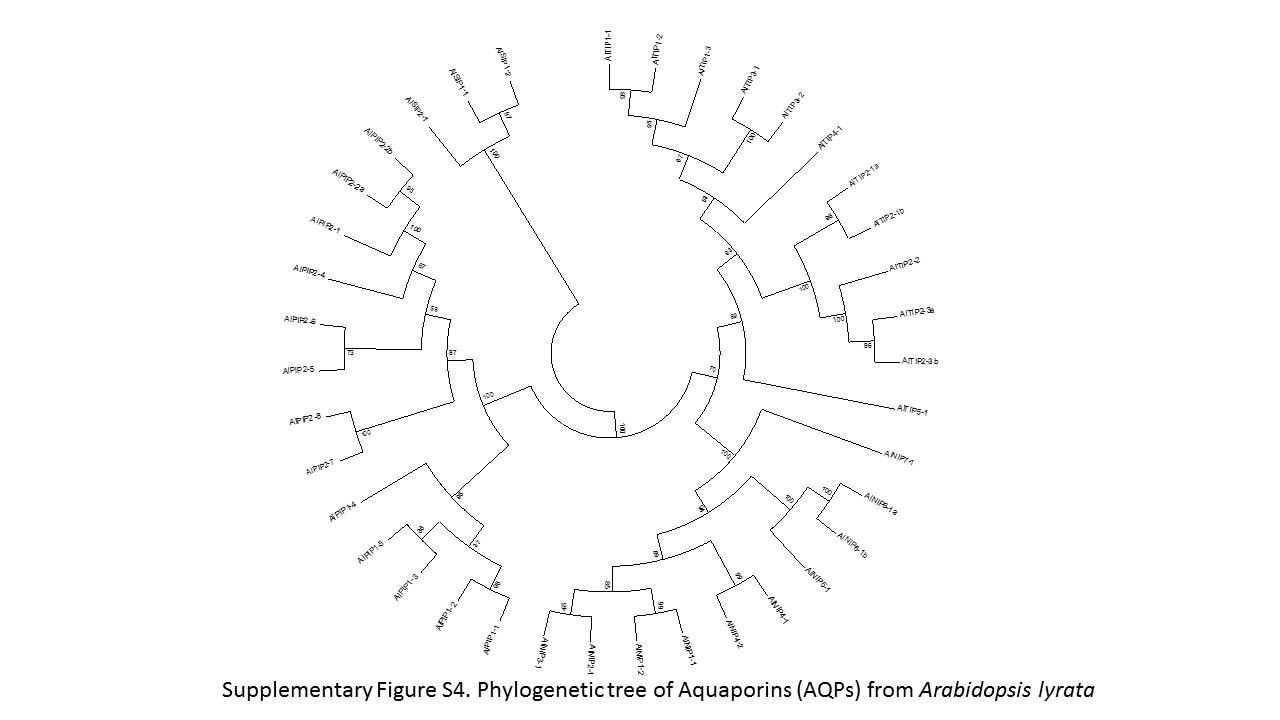


**Supplementary Figure 4** Phylogenetic tree of *Arabidopsis lyrata* aquaporins (AQPs)


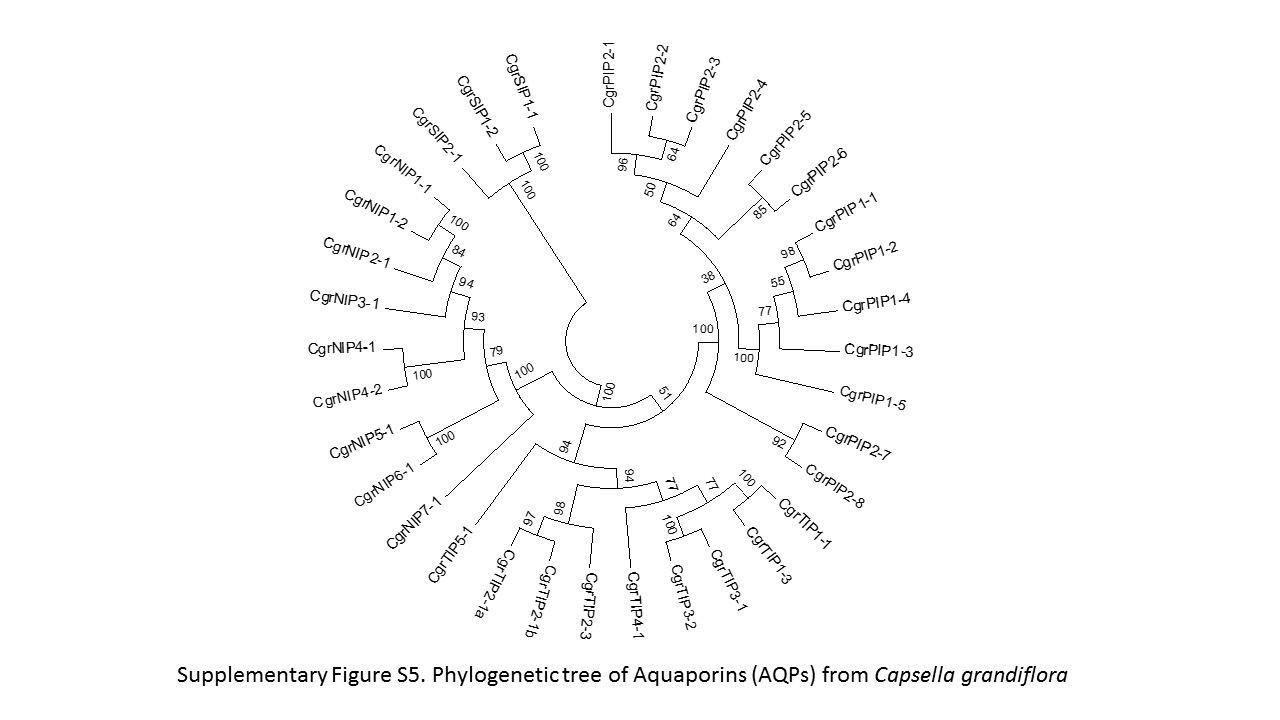


**Supplementary Figure 5** Phylogenetic tree of *Capsella grandiflora* aquaporins (AQPs)


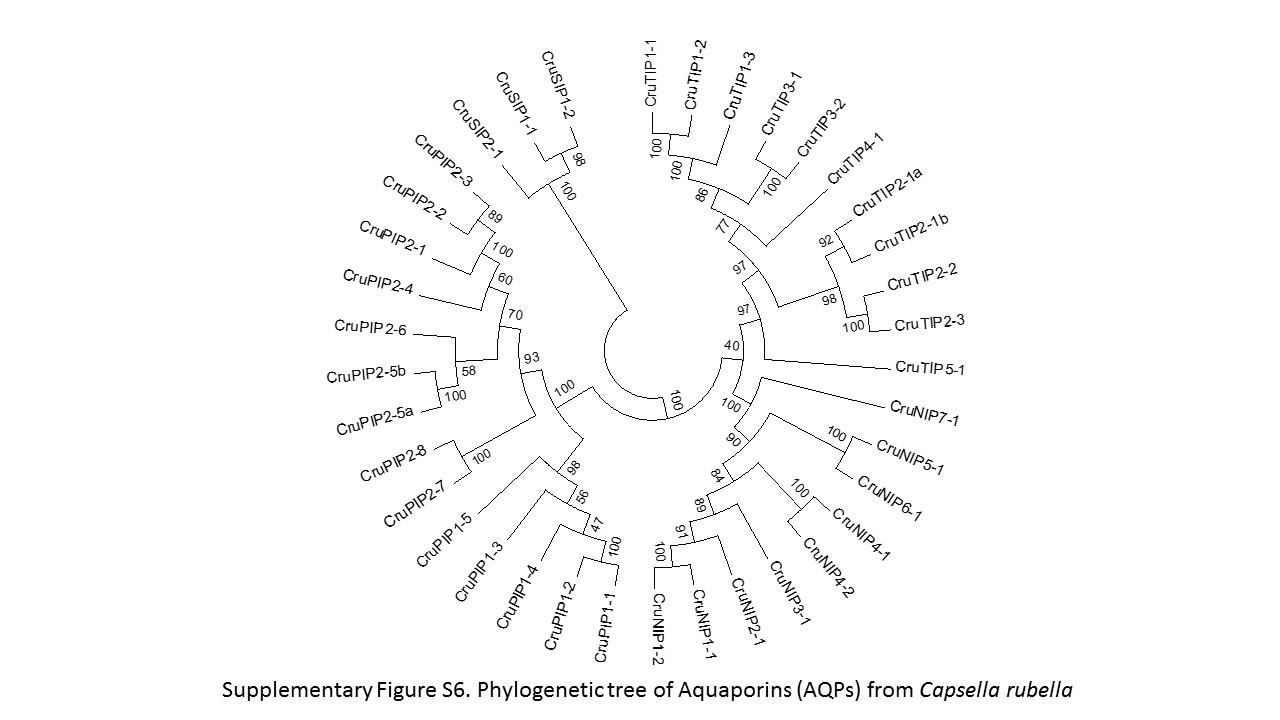


**Supplementary Figure 6** Phylogenetic tree of *Capsella rubella* aquaporins (AQPs)


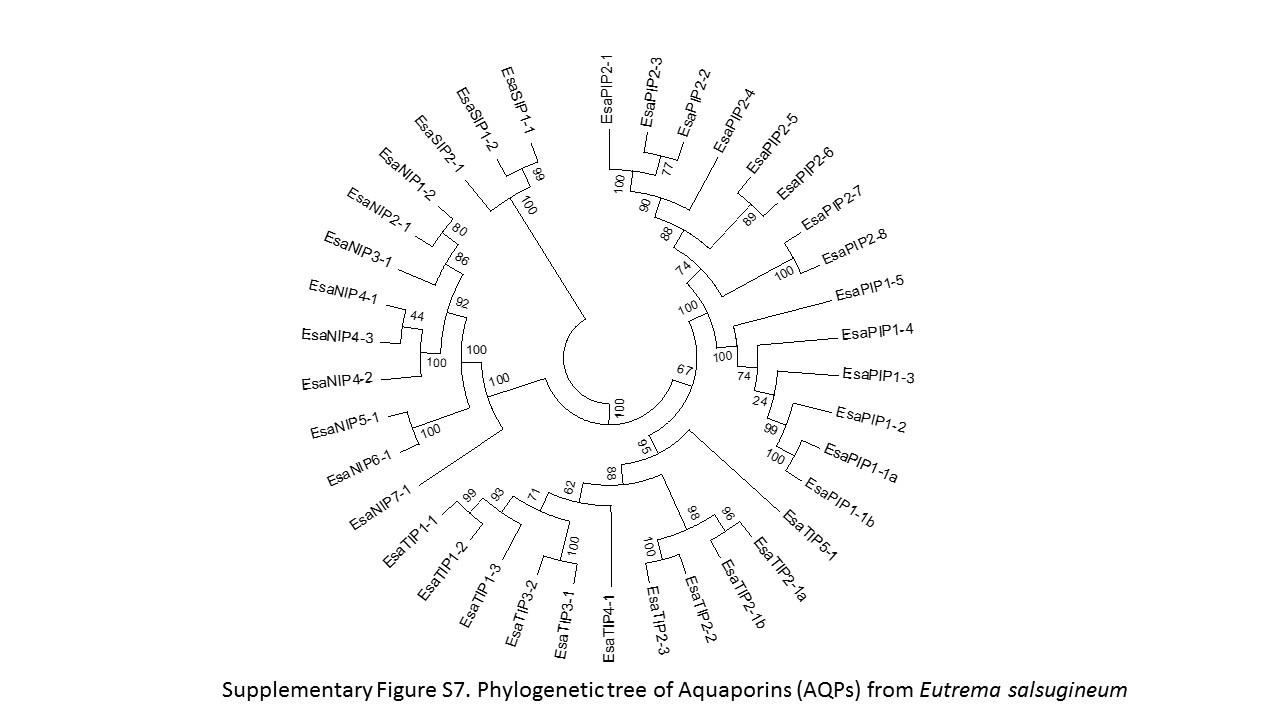


**Supplementary Figure 7** Phylogenetic tree of *Eutrema salsugineum* aquaporins (AQPs)

**Supplementary Figure 8** Distribution of aquaporins into subfamilies and groups based on a phylogenetic analysis performed in seven Brassicaceae species, and homology with known genes in Arabidopsis.


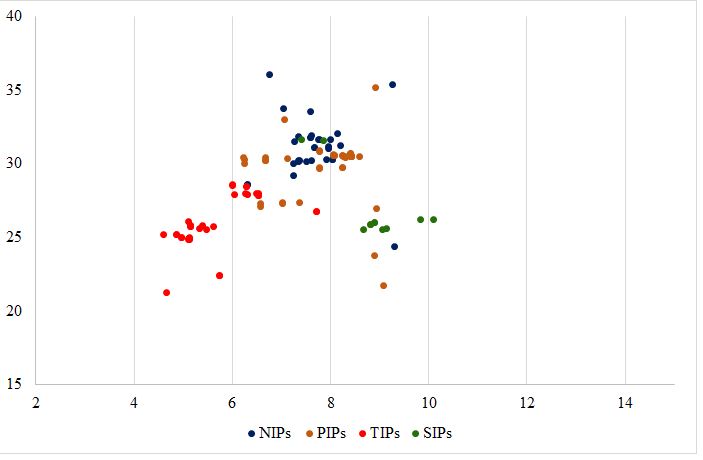


**Supplementary Figure 9.** Molecular weight and isoelectric point of *Brassica napus* aquaporins categorized into four sub-families namely plasma membrane intrinsic protein (PIPs); tonoplast intrinsic protein (TIPs); noduline-26 like intrinsic protein (NIPs) and; small intrinsic protein (SIPs).


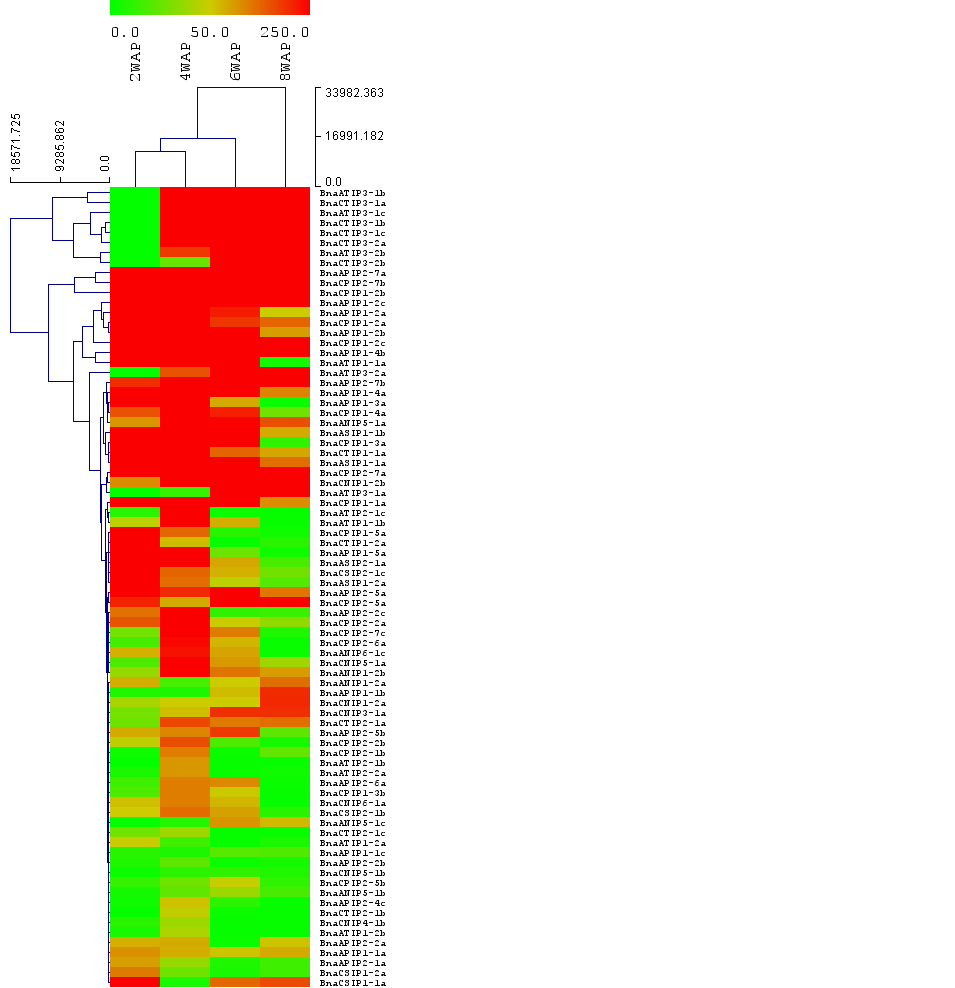


**Supplementary Figure 10.** Hierarchical clustering of aquaporins expressed during four seed developmental stages namely 2, 4, 6, and 8 weeks after pollination (WAP). RNA-seq expression data was obtained from SRA database with BioProject accession PRJNA311067 and the cluster analysis was performed using MeV [(http://www.tm4.org/mev.htm](http://www.tm4.org/mev.html)l).

**
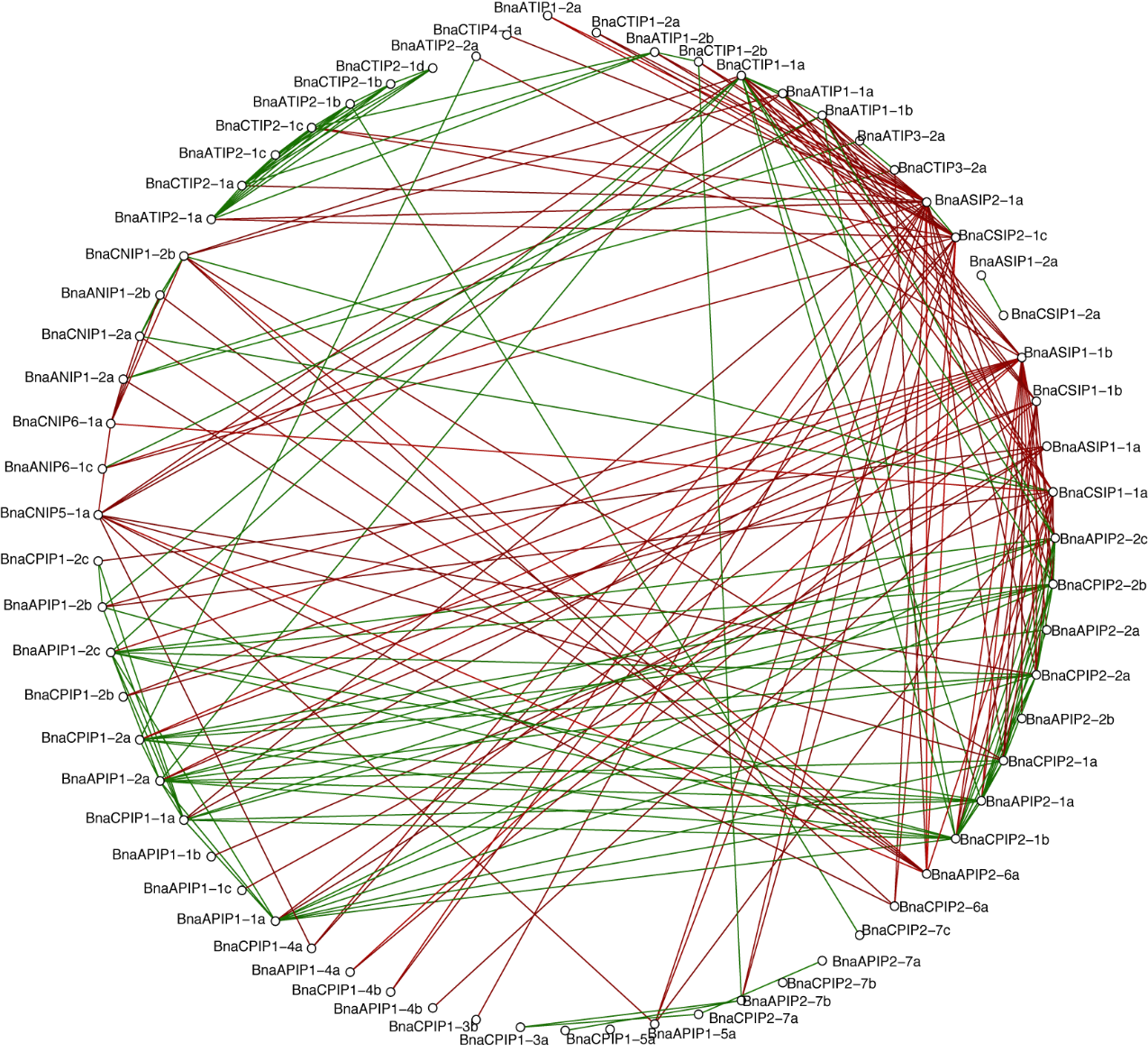
**

**Supplementary Figure 11.** Co-expression network of canola aquaporin genes constructed using the publically available RNA-seq data under the NCBI Bio-project accession PRJNA331148, PRJNA311316, PRJNA256233 and PRJNA311067. The network was obtained with Comparative Co-Expression Network Construction and Visualization tool (Tzfadia et al., 2015) and visualized by Cytoscape V. 3.3.0 (Shannon et al., 2003). Green and red lines denote positive correlation and negative correlation, respectively. Details of the expression data are provided in Supplementary Table 1

A)

B)


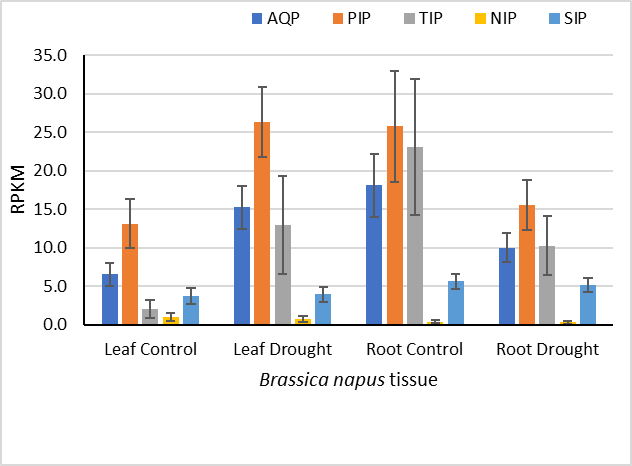

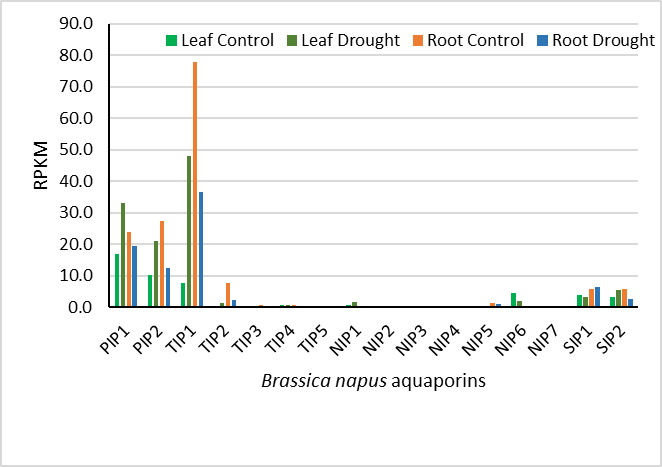


**Supplementary Figure 12** Expression of *Brassica napus* aquaporins (AQP) in leaf and root tissues under control and drought treatment. RNA-seq libraries were prepared with tissue isolated from three month-old plants. Graphs are showing expression levels in terms of average reads per kilobase of transcripts per million mapped reads (RPKM) for (A) four AQP subfamilies namely plasma membrane intrinsic protein (PIP); tonoplast intrinsic protein (TIP); noduline-26 like intrinsic protein (NIP) and; small intrinsic protein (SIP).; and (B) groups in each subfamily. Analysis of variance (ANOVA) showed significant differences among the conditions (p= 8.78 E-6) and among the genes (p= 5.7 E-42). Bars represent standard error from the mean for all values within a subfamily or group. Detailed data and results obtained with two-way ANOVA, without replication are provided in Supplementary Dataset 4.

Cluster 1

Cluster 2

Cluster 3

**Supplementary Figure 13.** Hierarchical clustering of aquaporins expressed in mature leaves inoculated with *Sclerotinia sclerotiorum* (Ssp), biocontrol agent *Pseudomonas chlororaphis* (PA23) and both combined together. RNA-seq expression data was obtained from SRA database with BioProject accession PRJNA331148 and the cluster analysis was performed using MeV [(http://www.tm4.org/mev.htm](http://www.tm4.org/mev.html)l).


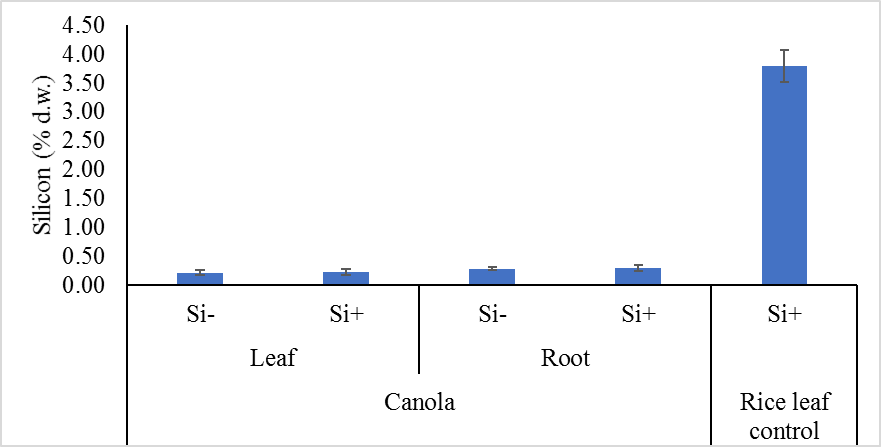


**Supplementary Figure 14.** Percent silicon (Si) content in leaves and roots of four week-old *Brassica* *napus* (canola) plants growing in a nutrient solution amended (Si+) or not (Si-) with 1.7 mM Si. Bars represent standard error from the mean (n = 5).

**Supplementary Information 2. Efflux Silicon Transporters in seven Brassicaceae species**

>Efflux Silicon Transporter of Arabidopsis lyrata-470187 pacid=16054829 transcript=470187 locus=470187 ID=470187.v1.107 annot-version=v1.0

MAMAPVIKLVLGSIAFAIFWVLAVFPSVPFLPIGRTAGSLFGAMLMVIFQ

VITPEQAYAAIDLPILGLLFGTMVVSIYLERADMFKYLGTLLSWKSKGPK

DLLCRVCLVSAVSSALFTNDTSCVVLTEFVLKIARQKNLPPHPFLLALAT

SANIGSSATPIGNPQNLVIAVQSKIPFWEFLLGVFPAMIVGITVNAMLLL

GMYWRLLSDHKEDEEEAGENADSEVLAVAEEDVTSHRFSPATFSPVASEE

SNFRMDPETLRNRAVSAGESELMSRDSNASREQTDAESQGESNVFHTKKW

RRVLWKSSVYLITLGMLISLVMGLNMSWTAITAALALVVLDFKDARPSLE

KVSYSLLIFFCGMFITVDGFNKTGIPTALWDLMEPYAKIDEAKGTAVLAV

VILVLSNVASNVPTVLLLGARVAASAMGREEEKKAWLLLAWVSTVAGNLT

LLGSAANLIVCEQARRAVSHGYTLTFTKHFKFGLPSTLIVTAIGLLLIK*

>Efflux Silicon Transporter of Brassica napus CDY55324 pep:annotated supercontig:AST_PRJEB5043_v1:LK033393:32600:37033:1 gene:GSBRNA2T00016675001 transcript:CDY55324 gene_biotype:protein_coding transcript_biotype:protein_coding gene_symbol:BnaC08g49750D

MNLNKFDKKNQSKASQEEEEGGSSSINGPYSSSSLHDSHIYFLPADTTKR

FQLWSVVVTKVFDSFYIVPTCERSDVIVQRLNSSLASSTSISCTTFNILA

PIYKRIDQLNQSNRESHSRDLWYTQYRPWDILELLLHQRSSVICLQEVWV

ANEELVNMYHDRLATAGYNIFQLPRTNRRGDGFRSTRILFLLNCSSGLAI

ETTGVTYAETSWVEAVFSIIKYQLQKASIAEDNKAFAFLGGNKQSDSLTY

SGFCQALQKANLTGIPHGLSFQETKELWVRADLARNGVFDYEKLKKTWNM

RTVDQSGKCKERVMESKKEEEAVGLKVKKAVLFPQETEKGLWPEDDYSLS

DHACLTHMAMAPVVKLVLGSIAFATFWILAVFPSVPFLPIGRTAGSLFGA

MLMVIFQVITPDQAYAAIDLPILGLLFGTMVVSIYLERADMFKYLGTLLS

WRSRGAKDLLCRVCLVSAVSSALFTNDTCCVVLTEFVLKIARQKNLPPHP

FLLALATSANIGSSATPIGNPQNLVIAVQSKISFWEFLRGVFPAMIVGIT

VNAVMLLAMYWRLLSDHKEEEEIEVSEGVVAVEEEDVMSHRFSPATLPHL

SSFRSEETSSYPTVNSNALLFQTKRWRRVLWKSSVYLITLGMLISLLMGL

NMSWTAITAALALVVLDFKDARPSLEKVSYSLLIFFCGMFITVDGFNKTG

IPTALWDLMEPYANIGEAKGTAVLAVVILVLSNVASNVPTVLLLGARVAA

SAAAGEEEKKAWLLLAWVSTVAGNLTLLGSAANLIVCEQARRAVSHGYTL

TFTKHLKFGLPSTLIVTAIGLYLIK

>Efflux Silicon Transporter of Brassica napus CDY32451 pep:annotated supercontig:AST_PRJEB5043_v1:LK032295:75206:76917:-1 gene:GSBRNA2T00052420001 transcript:CDY32451 gene_biotype:protein_coding transcript_biotype:protein_coding gene_symbol:BnaA09g51450D

MAMAPVVKLVLGSIAFAIFWILAVFPSVPFLPIGRTAGSLFGAMLMVIFQ

VITPDQAYAAIDLPILGLLFGTMVVSIYLERADMFKYLGTLLSWRSRGPK

DLLCRVCLVSAVSSALFTNDTCCVVLTEFVLKIARQKNLPPHPFLLALAT

SANIGSSATPIGNPQNLVIAVQSKISFWEFLRGVFPAMIVGITVNAVMLL

AMYWRLLSDHKEEEENEVSEGVVAVEEEDVTSHRFSPATNRGASSGESSA

SRDHQADAESQGESYPTTINSNVLLFQTKRWRRVLWKSSVYLITLGMLIS

LLMGLNMSWTAITAALALVVLDFKDARPSLEKVSYSLLIFFCGMFITVDG

FNKTGIPTALWDLMEPYANIDEAKGTAVLALVILVLSNVASNVPTVLLLG

ARVAASAAAGEEEKKAWLLLAWVSTVAGNLTLLGSAANLIVCEQARRAVS

HGYTLTFTKHLKFGLPSTLIVTAIGLYLIK

>Efflux Silicon Transporter of Brassica oleraceae Bo8g117550.1 pep:novel chromosome:v2.1:C8:41328198:41329795:1 gene:Bo8g117550 transcript:Bo8g117550.1 gene_biotype:protein_coding transcript_biotype:protein_coding description:Divalent ion symporter [Source:Projected from Arabidopsis thaliana (AT1G02260) TAIR;Acc:AT1G02260]

MAMAPVVKLVLGSIAFATFWILAVFPSVPFLPIGRTAGSLFGAMLMVIFQ

VITPDQAYAAIDLPILGLLFGTMVVSIYLERADMFKYLGTLLSWRSRGAK

DLLCRVCLVSAVSSALFTNDTCCVVLTEFVLKIARQKNLPPHPFLLALAT

SANIGSSATPIGNPQNLVIAVQSKISFWEFLRGVFPAMIVGITVNAVMLL

AMYWRLLSDHKDQEEEENEVSEGVVAVEEEDVTSHRFSPATLPHLSSFRS

EETNVRTDPETLRNRGASSGESSASRDHQADAESQGESYPTVNSNVLLFQ

TKRWRRVLWKSSVYLITLGMLISLLMGLNMSWTAITAALALVVLDFKDAR

PSLEKVSYSLLIFFCGMFITVDGFNKTGIPTALWDHMEPYANIGEAKGTA

VLAVVILVLSNVASNVPTVLLLGARVAASAAAGEEEKKAWLLLAWVSTVA

GNLTLLGSAANLIVCEQARRAVSHGYTLTFTKHLKFGLPSTLIVTAIGLY

LIK

>Efflux Silicon Transporter of Brassica rapa Bra032614.1-P pep:predicted chromosome:IVFCAASv1:A09:36843098:36844694:1 gene:Bra032614 transcript:Bra032614.1 gene_biotype:protein_coding transcript_biotype:protein_coding description:AT1G02260 (E=1e-213) | transmembrane protein, putative

MAMAPVVKLVLGSIAFAIFWILAVFPSVPFLPIGRTAGSLFGAMLMVIFQ

VITPDQAYAAIDLPILGLLFGTMVVSIYLERADMFKYLGTLLSWRSRGPK

DLLCRVCLVSAVSSALFTNDTCCVVLTEFVLKIARQKNLPPHPFLLALAT

SANIGSSATPIGNPQNLVIAVQSKISFWEFLRGVFPAMIVGIIVNAVMLL

AMYWRLLSDHKEEEENEVSEGVVAVEEEDVTSHRFSPATLPHLSSFRSEE

TNGRTDPETLRNRGASSGESSASRDHQADAESQGESYPTTINSNVLLFQT

KRWRRVLWKSSVYLITLGMLISLLMGLNMSWTAITAALALVGLDFKDARP

SLEKVSYSLLIFFCGMFITVDGFNKTGIPTALWDLMEPYANIDEAKGTAV

LALVILVLSNVASNVPTVLLLGARVAASAAAGEEEKKAWLLLAWVSTVAG

NLTLLGSAANLIVCEQARRAVSHGYTLTFTKHLKFGLPSTLIVTAIGLYL

IK

>Efflux Silicon Transporter of Capsella grandiflora Cagra.1968s0039.1.p pacid=28910152 transcript=Cagra.1968s0039.1 locus=Cagra.1968s0039 ID=Cagra.1968s0039.1.v1.1 annot-version=v1.1

MAMAPVIKLVLGSIAFAIFWVLAVFPSVPLLPIGRTAGSLFGAMLMVIFQ

VITPEQAYAAIDLPILGLLFGTMVVSIYLERADMFKYLGTLLSWKSRGPK

DLLCRVCLVSAVSSALFTNDTSCVVLTEFVLKIARQKNLPPHPFLLALAT

SANIGSSATPIGNPQNLVIAVQSKIPFWEFLLGVFPAMIVGVTVNALLLL

GMYWRLLSDHKEDEEKAGESADSEVVAVAEEDVTSHRFSPATFSPATSEE

SNLRMDPDTLRNRAGSAGEGDLISRDSNASREQQTDAESQGESNNNNNNM

FQTKKWRRVIWKSSVYLITLGMLISLLMGLNMSWTAITAALALVVLDFKD

ARPSLEKVSYSLLIFFCGMFITVDGFNKTGIPTALWDLMEPYAKIDQAKG

TAVLAVVILVLSNVASNVPTVLLLGARVAASAMGREEEKKAWLLLAWVST

VAGNLTLLGSAANLIVCEQARRAVSHGYTLTFTKHFKFGLPSTLIVTAIG

LFLIK*

>Efflux Silicon Transporter of Capsella rubella Carubv10008913m|PACid:20892720

MAMAPVIKLVLGSIAFAIFWVLAVFPSVPLLPIGRTAGSLFGAMLMVIFQ

VITPEQAYAAIDLPILGLLFGTMVVSIYLERADMFKYLGTLLSWKSRGPK

DLLCRVCLVSAVSSALFTNDTSCVVLTEFVLKIARQKNLPPHPFLLALAT

SANIGSSATPIGNPQNLVIAVQSKIPFWEFLLGVFPAMIVGVTVNALLLL

GMYWRLLSDHKEDEEKAGESADSEVVAVAEEDVTSHRFSPATFSPATSEE

SNLRMDPDTLRNRAGSAGEGDLISRDSNASREQQTDAESQGEINNNNNMF

QTKKWRRVLWKSSVYLITLGMLISLLMGLNMSWTAITAALALVVLDFKDA

RPSLEKVSYSLLIFFCGMFITVDGFNKTGIPTALWDLMEPYAKIDQAKGT

MVLAVVILVLSNVASNVPTVLLLGARVAASAMGREEEKKAWLLLAWVSTV

AGNLTLLGSAANLIVCEQARRAVSHGYTLTFTKHFKFGLPSTLIVTAIGL

FLIK*

>Efflux Silicon Transporter of Eutrema salsugineum Thhalv10007447m pacid=20187529 transcript=Thhalv10007447m locus=Thhalv10007447m.g ID=Thhalv10007447m.v1.0 annot-version=v1.0

MAMAPVIKLVLGSIAFAIFWVLAVFPSVPFLPIGRTAGSLFGAMLMVIFQ

VITPEQAYAAIDLPILGLLFGTMVVSIYLERADMFKYLGTLLSWRSRGPK

DLLCRVCLVSAVSSALFTNDTSCVVLTEFVLKIARQKNLPPHPFLLALAT

SANIGSSATPIGNPQNLVIAVQSKIPFWEFLLGVFPAMAVGITVNALILL

AMYWRLLSDHKDDEEEEEVEEENAVSEAVAEEDVTSHRFSPATLPHLSSF

RSQESNLRMDPETLRNRAVSAGESDIMNRDSNASNAESQGESFNTSKNWR

RVLWKSSVYLITLGMLISLLMGLNMSWTAITAALALVVLDFKDARPSLEK

VSYSLLIFFCGMFITVDGFNKTGIPTALWDLMEPYAKIDQAKGTAVLALV

ILVLSNVASNVPTVLLLGARVAASAAAGEEEKKAWLLLAWVSTVAGNLTL

LGSAANLIVCEQARRAVSHGYTLTFAKHFKFGLPSTLIVTAIGLLLIK*
